# Still very much dead and alive: Incoherence in conspiracism as a departure from Bayesian rationality

**DOI:** 10.1093/pnasnexus/pgag250

**Published:** 2026-08-18

**Authors:** Alessandro Miani, Nicole Cruz, Stephan Lewandowsky

**Affiliations:** Department of Psychology, University of Fribourg, Rue P.-A.-de-Faucigny 2, CH-1700 Fribourg, Switzerland; School of Psychological Science, University of Bristol, 12a Priory Road, Bristol BS8 1TU, United Kingdom; Department of Psychology, University of Potsdam, Karl-Liebknecht-Str. 24-25, 14476 Potsdam, Germany; School of Psychological Science, University of Bristol, 12a Priory Road, Bristol BS8 1TU, United Kingdom; Department of Psychology, University of Potsdam, Karl-Liebknecht-Str. 24-25, 14476 Potsdam, Germany

**Keywords:** conspiracy theories, conspiracism, probabilistic coherence, Bayesian rationality

## Abstract

Incoherence has long been considered a hallmark of belief in conspiracy theories, though recent research has cast doubt on this claim. We identify theoretical and methodological shortcomings in previous assessments of incoherence and connect this work to current research on reasoning by modeling conspiracism as a set of probabilistic beliefs and computing incoherence as the departure from Bayesian principles of rational belief. In study 1, reanalyzing data from eight studies involving 8,590 participants, we examined the relationship between conspiracism and belief in mutually incompatible conspiracy theories (e.g. Princess Diana “was murdered” and “faked her own death”). A robust association with conspiracism emerged, with the strongest conspiracy believers showing a 91% probability of being incoherent. In study 2, a preregistered experiment with 469 participants, we investigated whether incoherence is specific to conspiratorial contents related to a person’s worldview, or reflects a more general tendency toward inconsistency. We manipulated the content domain (conspiracy vs. nonconspiracy) and logical relation (contrary vs. contradictory) of incompatible explanations for fictitious vignettes and collected explicit probability ratings. We found evidence for a two-component effect of incoherence: a general component, associated with reduced logical monitoring, that emerges in contradictory pairs, and a content-specific component, associated with worldview-driven endorsement, that emerges in contrary conspiracy pairs. Together, these findings encourage a move from dichotomous judgments of dis/belief to a probabilistic modeling of conspiracy beliefs, grounded in a rigorous definition of logical incompatibility and a Bayesian framework for measuring incoherence.

Significance statement A recent series of studies questioned whether conspiratorial incoherence (the simultaneous belief in logically incompatible conspiracy theories such as “Princess Diana was murdered” and “she faked her own death”) is truly a hallmark of conspiracism. Reanalyzing data from eight studies and implementing a novel experimental design, we find robust associations between conspiracism and incoherence, and disentangle two underlying mechanisms: worldview-driven endorsement of incompatible claims and a reduced logical monitoring. Our findings resolve apparent contradictions in the literature, highlight the importance of measuring incoherence at the individual level, and offer more precise conceptual and statistical tools for understanding incoherence within belief systems.

## Introduction

Incoherence has been repeatedly identified as an attribute of conspiracy theories (CTs) (1–6). In a pioneering article, Wood et al. (1) showed that the more participants believed one conspiratorial account, namely that Princess Diana had faked her own death, the more they also believed the alternative—and mutually exclusive—conspiracy that she was murdered by MI6, the British secret service. These findings have been replicated repeatedly (7–10). The common denominator among those studies was the observation of a positive correlation between belief in incompatible CTs (expressed on Likert scales).

This research has however been met with some skepticism. One critique points out that in the presence of uncertainty, it can be reasonable to maintain several incompatible hypotheses. For example if we misplaced our key ring, it would not be unreasonable to suspect that we left it in the front door lock while also entertaining the possibility that we left it in the kitchen (11). An alternative critique targeted the empirical basis for the claimed incoherence, based on a reassessment of the observed positive correlations between responses to competing CTs. In a series of four preregistered studies, van Prooijen et al. (12) provided empirical evidence in support of this suspicion: the observed positive correlations were mostly driven by participants who were *skeptical* of (instead of *endorsing*) CTs. The authors found that on average, <3% of participants endorsed mutually incompatible CTs, concluding that “researchers have overestimated the predictability and prevalence of such inconsistencies in a conspiratorial mindset” (p. 680). The literature on conspiratorial incoherence thus appears to be contradictory itself.

Here, we reconcile the apparent contradictions in the literature, provide the strongest evidence to date for conspiratorial incoherence, and advance the theoretical understanding of incoherence associated with conspiracism. First, we start by demonstrating that previous assessments of incoherence, whether offered in support (1) or as criticism (12), were of limited diagnosticity. Second, we introduce a measure based on probabilistic coherence to quantify participants’ incoherence: instead of correlating responses (1, 12) or counting instances of mutual endorsement (8, 9), we interpret agreement ratings as subjective degrees of belief (13) expressed as probabilities within a Bayesian framework. We show that our measure has several advantages compared to existing methods. Finally, with two studies applying our measure to extant (study 1) and novel (study 2) data, we confirm a strong relationship between incoherence and conspiracism and propose that this incoherence reflects two components: a general component (reduced logical monitoring) and a content-specific component (worldview-driven endorsement).

### A framework for studying conspiratorial incoherence

Research in social psychology on stereotyping shows that people can simultaneously hold multiple, even incompatible, beliefs when these beliefs align with a strongly held worldview. For example, individuals high in antisemitism have been shown to endorse both the belief that “Jewish people are seclusive and clannish” and the incompatible one that “they are over-assimilative” (14). Similarly, people opposed to transgender-inclusive rights often support mutually inconsistent anti-transgender arguments (15). In each case, a strong ideological commitment provides a higher-order interpretive framework that accommodates logically incompatible claims.

Similar patterns emerge in conspiracism. One of the most reliable findings in the psychology of CTs is that belief in one CT predicts belief in other CTs. Researchers have reported that unrelated (16), fictitious^a^ (17), and even logically incompatible CTs correlate with each other. This observation has led researchers to describe conspiracism as a network of mutually supportive beliefs (1, 4, 16–18) wherein individual CTs support each other by reinforcing an overarching conspiratorial worldview. This worldview, characterized by a skepticism toward mainstream narratives, in turn, provides support for various CTs, regardless of their logical compatibility. In support, a large-scale network analysis of conspiratorial (vs. nonconspiratorial) narratives, has revealed that conspiracy texts are in fact highly interconnected by co-occurring but unrelated topics (4). This has been formalized as the *in/coherence* framework.

The in/coherence framework can explain the endorsement of logically incompatible CTs: in the mind of the conspiracy believer, the incoherence stemming from two incompatible CTs is resolved by their coherence within a higher-level conspiratorial worldview (1, 2). Believing that the world is dominated by conspiracies allows individuals to simultaneously accept that Princess Diana has been murdered and that she faked her own death. Despite being logically incompatible, both CTs are coherent with the overarching idea that *the official narrative is not true*.

Several studies indicate a broader, domain-general tendency toward incoherent thinking among individuals high in conspiracism (5, 6, 8, 9). This line of work has consistently reported a positive association between conspiracism and general incoherence. However, evidence for conspiratorial incoherence, namely the endorsement of logically incompatible CTs as a function of conspiracism, has recently been met with skepticism. In particular, van Prooijen et al. (12) correctly identified a methodological flaw in this research tradition but may have been too quick to encourage scholars to “reconsider the notion of systematic belief in contradictory conspiracy theories” (p. 670). Their work, unlike the research on domain-general proneness to incoherence, cast doubt on the magnitude of conspiratorial incoherence. In what follows, we lay out the conceptual meaning and criteria for incoherence and identify the terminological, methodological, and statistical limitations that have contributed to these inconsistent conclusions.

### Limitations in previous assessments of incoherence

Previous research has yielded estimates of incoherence that are at best inconsistent (ranging from 3% (12) to 15–48% (7–10)). Traditionally, conspiratorial incoherence has been evaluated via the correlation between responses to incompatible items, with a higher correlation being seen as indicating greater incoherence (1, 7, 10). At first glance, this seems entirely plausible because if people who are more willing to endorse Princess Diana’s murder by MI6 (by providing a higher numeric value on a Likert scale) are also more willing to endorse the idea that she faked her own death, then one should indeed observe a positive correlation between both response scales across participants. However, there are several problems with this operationalization.

First, a positive correlation between responses to two incompatible CTs can arise even when participants uniformly reject both.^b^ If individuals who strongly disbelieve one theory also strongly disbelieve the other, their responses will be positively correlated across participants, despite the fact that none (or very few) actually endorse either conspiracy. Indeed, van Prooijen et al. (12) demonstrated this pattern empirically. Second, even when a positive correlation reflects joint endorsement rather than joint rejection, the correlation coefficient itself does not reliably indicate the degree of incoherence. Depending on how participants’ responses are distributed along the response scale (e.g. clustered at either dis/agreement), a given magnitude of correlation is not indicative of a participant’s level of incoherence. A detailed illustration of these cases is shown in the Section SM1.

There is an additional limitation to the correlational approach. Even if correlation coefficients accurately gauged incoherence (which they do not), they describe only patterns at the sample level and cannot characterize individual participants. The correlation approach thus prevents researchers from associating an individual’s degree of incoherence with other potential explanatory variables, such as their level of conspiracism or other relevant psychological traits.

The issue of obtaining an individual-level measure of incoherence was addressed by Petrović and Žeželj (8, 9) in a study in which participants rated their agreement with pairs of logically incompatible statements on a Likert scale. Responses were then dichotomized into *disagreement* (0) and *agreement* (1), depending on whether they fell below or above the midpoint (*unsure*). Each participant’s incoherence score was calculated as the number of incompatible pairs endorsed simultaneously. This measure is diagnostic: incoherence stemming from incompatible CTs correlates with general proneness to incoherence, specific CTs, and conspiracy mentality (8, 9). This measure loses information at the item level through dichotomization, although it still allows for graded values in the aggregate.

The limitations of the common measures are accompanied by limitations of terminology. Scholars have frequently (if not always) used the term “contradictory” to refer to statements that are logically incompatible (1, 7–9, 12). While this has become conventional in social psychology, it is conceptually imprecise and methodologically problematic. Clarifying the distinction between different forms of logical incompatibility is crucial for a consistent interpretation of what counts as incoherence in conspiratorial thinking.

A first issue concerns the operationalization of logical incompatibility as a matter of degree. For example, Lukić et al. (7) introduced a distinction between *probable* and *necessary* mutual exclusion, based on the perceived likelihood that two events could co-occur. Similarly, Petrović and Žeželj (8) asked experts (postgraduate students in philosophy) to evaluate the degree of incompatibility between pairs of statements. The problem with these approaches is that logical incompatibility is not a matter of degree: two statements are either incompatible or they are not. Thus, attempting to quantify the extent to which two statements are logically incompatible is conceptually mistaken from a logical standpoint (even if such judgments may be psychologically interesting). These ratings reflect subjective impressions of compatibility, not logical relations. Logical in/compatibility is a logical concept that refers to structural properties of statements. Hence the concept holds or not for a pair of statements by definition, not by the personal opinion or subjective preference of a person rating it.

A second issue concerns the widespread misuse of the term “contradictory”, which represents only a specific subset of logical incompatibilities (see Ref. (19)). The use of the term in the literature to indicate a general incompatibility between statements diverges from its meaning according to the taxonomy of classical logic. In fact, two statements are *contradictory* only if they cannot both be true *and* cannot both be false at the same time: the truth of one necessarily implies the falsity of the other because they exhaust all logical possibilities regarding a specific event (e.g. *Diana is dead* and *Diana is not dead*). By contrast, two statements are *contrary* if they cannot both be true but can both be false. For instance, the claims *Diana faked her own death* and *Diana killed by MI6* are contrary: both cannot be true simultaneously, but both may be false (e.g. if she died in a car accident). Finally, two statements are *compatible* when both can (in principle) be true or false at the same time: the truth of one does not constrain the truth of the other. An example is the pair *Tito was an American spy* and *Tito was a Russian spy*. While this may seem implausible, both could be true (if the former Yugoslav leader Tito were a double agent) or both false (if he were not a spy at all).

A third issue concerns the fact that genuinely contradictory conspiratorial statements are, in principle, impossible. True contradictions require one statement to assert what the other denies. For example, consider the pair: A =  *Diana was killed by MI6* and B =  *Diana was **not** killed by MI6*. These two statements are truly contradictory, but statement B is not conspiratorial. To make it conspiratorial, one might modify it as C =  *Diana was not killed by MI6 but by the CIA*, but the relation between A and C is no longer one of contradiction but of contrariety (see Section SM2 for a more thorough discussion).

Accordingly, we reanalyzed the item pairs used in prior studies (7–9, 12, 20) following a taxonomy from classical logic (cf. (19)). We found that none represent true contradictions; instead, most pairs are actually contraries, and some are even compatible (see Section SM3). In what follows, we introduce a new measure based on a more rigorous definition of logical incompatibility that circumvents the problems of nondiagnosticity and lack of sensitivity.

## A diagnostically valid measure of incoherence

Conspiracy beliefs do not form a dichotomous system of dis/belief but rather a continuous, probabilistic construct (13) where uncertainty plays a central role (21). Experimental evidence shows that participants adjust their endorsement of conspiratorial explanations when the probability of an event is manipulated: an event described as less (vs. more) likely, receives higher (vs. lower) endorsement (22). Likewise, qualitative interviews indicate that individuals interpret conspiratorial explanations as graded possibilities, evaluating the likelihood of each scenario, even when they are mutually incompatible (7).

We therefore encourage a move from the dichotomous system of dis/belief (8, 9) to the probabilistic modeling of conspiracy beliefs (13, 22), which brings the research more in line with current practice in research on reasoning. While logical relations between statements are binary, the degrees of belief assigned to them are continuous and must satisfy the constraints determined by probability theory and by the logical structure of the statements involved. Contrary to the dichotomous interpretation of beliefs, in a Bayesian approach, individuals may rationally hold graded degrees of belief in multiple mutually exclusive hypotheses, provided that the total probability assigned to them does not exceed unity. Thus, it is entirely rational to entertain the hypothesis that a key ring may be in the door lock *or* in the kitchen (e.g. with P=0.50 for each; (11)). By contrast, it is not permissible to be nearly certain of two mutually exclusive hypotheses simultaneously—e.g. the key being in the lock *and* on the table (e.g. P=0.90 each; (23–25)).

Practically speaking, to measure violations of the basic constraints of probability theory, participants’ ordinal responses can be treated as coarse (depending on the number of Likert point chosen by researchers) yet informative approximations of subjective probabilities. This is achieved by mapping Likert-scale responses (from strongly disagree to strongly agree) onto a probabilistic scale (0–1) using a monotonic transformation that preserves ordinal relations and enables formal assessment of coherence within probability theory (26). This approach is grounded in the Bayesian interpretation of probability as the logic of partial belief (27, 28), which links probabilistic judgments to ordinal preferences and fair-bet intuitions (23, 29, 30). Empirical research consistently demonstrates a close correspondence between Likert ratings and probabilistic assessments (31–38). For example, in studies that explored the conjunction fallacy, when neutral scenarios were used in which no stereotypical cues push participants toward the fallacy, coherence rates were found to be virtually identical whether responses were collected with ordinal scales (such as Likert-type ratings) or with continuous probability estimates (37, 38).

We illustrate this idea by considering three mutually exclusive (contrary) explanations for Princess Diana’s death: A =  *Diana faked her own death*, B =  *Diana was murdered by MI6*, C =  *Diana died in a car accident in Paris* (the official narrative). Suppose we assign an 80% subjective probability to A ($P_{A}=0.80$). This leaves P=0.20 to be distributed among B and C or residual uncertainty. Coherence requires that $P_{A}+P_{B}+P_{C}\leq 1$. Therefore, assigning $P_{A}=0.90$, $P_{B}=0.40$, and $P_{C}=0.20$ would yield $P_{\mathrm{total}}=1.50$, violating probabilistic coherence. To put it with a more concrete analogy, a single *unit* of pizza can be divided into any number of slices, but the total quantity cannot exceed the whole pizza. One cannot eat 1.50 pizza when only one is available, unfortunately. But note, one may choose not to take any slice at all (or a slice of size zero); this is possible because in a set of contrary statements, all can be false.^c^

**Figure 1:**
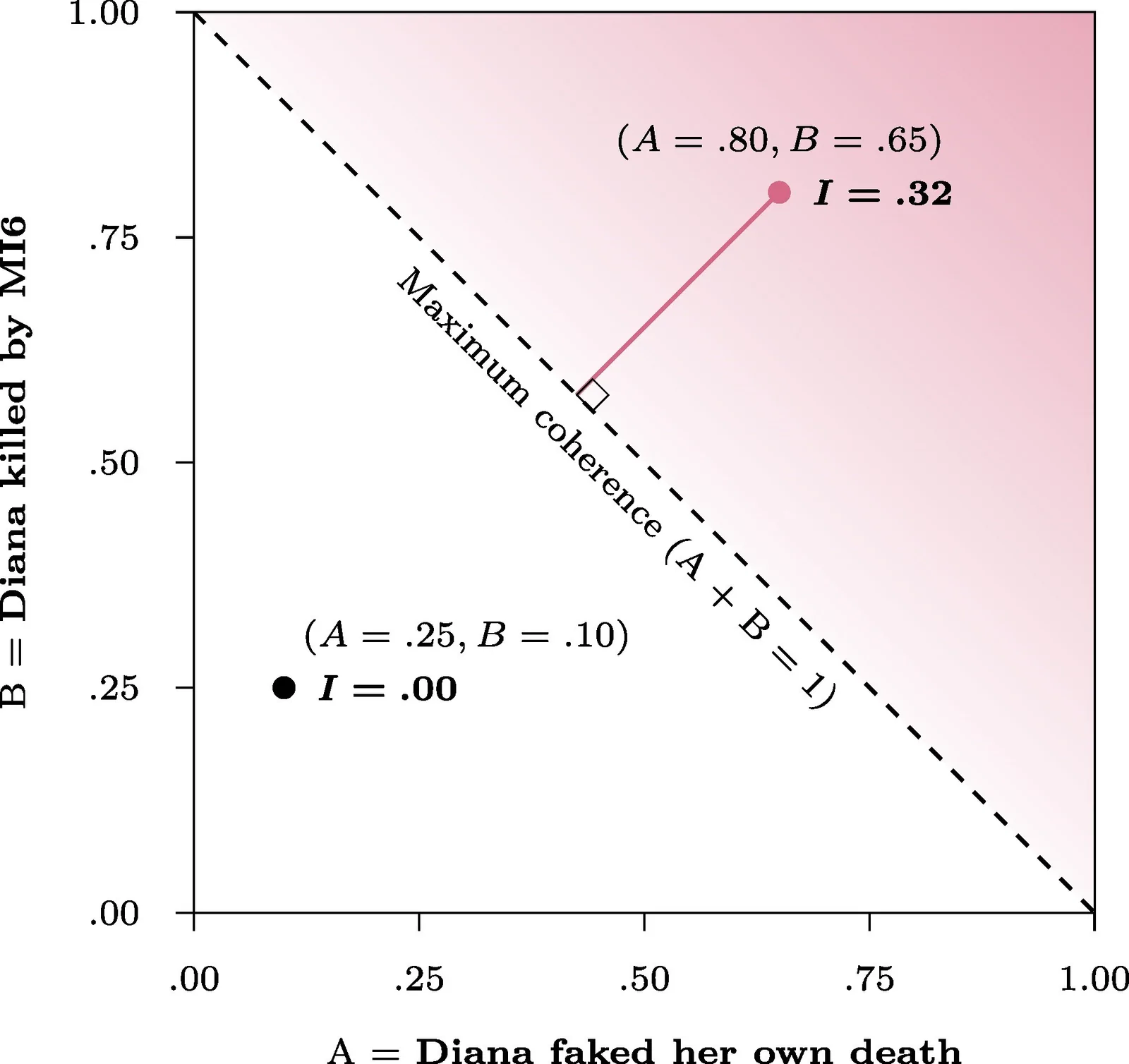
The Incoherence Score *I*. The dashed diagonal line represents the coherence boundary calculated as the sum of subjective probabilities of A and B equal to 1. The top-right (vs. bottom-left) point illustrates a participant’s incoherent (vs. coherent) response, with the corresponding value of *I* (see text for details). The perpendicular solid line to the dashed diagonal line indicates the Euclidean distance from the point representing a participant’s response to items A and B, to the coherence boundary, measured perpendicularly, the length of which defines our incoherence score *I*.

As illustrated in Figure 1, incoherence arises when responses to contrary statements fall beyond the coherence boundary, which occurs when the sum of probabilities exceeds unity. We quantify this deviation using an incoherence score, *I*, defined as the Euclidean distance between the observed response pair and the nearest point on the coherence boundary (A+B=1). This distance measure, commonly used in research fields concerned with the study of uncertain beliefs (24, 39, 40), returns I=0 for coherent responses ($P_{A}+P_{B}\leq 1$) and increases proportionally with incoherence ($P_{A}+P_{B}>1$; see Section SM4). This approach allows us to both detect the *presence* of incoherence (dichotomized as I>0) and to *quantify* the distance from a coherent response (with a continuous score ranging from I>0 to I≤0.71).

Compared to previous assessments, the *I* score offers several important advantages (see Section SM5). It successfully discriminates between groups theoretically expected to differ in incoherence where correlations fail. Compared to the binary score (8, 9), it shows greater sensitivity, higher internal consistency, and improved predictive validity. Note that although the *I* score outperforms binary scoring, the latter is still useful to estimate the proportion of individuals who accept (or reject) specific beliefs. Those estimates are impossible without a dichotomization (10, 41).

The *I* score can incorporate more than two contrary statements. This multidimensional extension is well established in coherence research (24, 30, 42), where coherence requires that A+B+⋯+n≤1. As more contrary statements are added, the range of probabilities available for coherent responses shrinks, decreasing the chance rate of being coherent (43).

While we use probabilistic coherence as a normative benchmark, we do not assume that respondents behave as perfectly rational agents. Rather, empirical evidence shows that individuals tend to approximate the rules of probability theory while exhibiting small and symmetric deviations; in other words, people are frequently rational (36, 44), but fluctuate around the normative constraints in predictable ways. Such fluctuations reflect natural variability and well-known biases in human probabilistic reasoning, and are expected to average out across individuals (44). Departures from coherence become interesting in new ways when they show a systematic association with a psychological construct like conspiracism. We next examine this possibility in a reanalysis of relevant existing studies.

## Study 1

Our aim is to reconcile the apparent inconsistencies in the literature and provide a reliable estimate for conspiratorial incoherence through a reanalysis of existing data. We seek to show that (a) the propensity of incoherence increases with people’s propensity to endorse CTs, for example as reflected in their level of conspiracism, and, (b) more generally, that incoherence is a notable attribute of believers in CTs. These goals require two inclusion criteria for previous studies. First, studies are relevant only if they presented participants with a series of incompatible pairs of CTs. Second, because incoherence is theorized to be an attribute of conspiratorial beliefs, studies were included only if they independently assessed participants’ level of conspiracism. That is, studies had to assess conspiracism using a measure other than the responses that are to be examined for incoherence. This permits a noncircular, independent identification of those participants for whom we expect to observe a particularly high (or low) degree of departure from coherence.

### Methods

Four published articles (with a total of eight studies) satisfied the inclusion criteria, namely those of Lukić *et al.* (7, LK19 from here on), Petrović and Žeželj (9, PT21), (8, PT22), and van Prooijen et al. (12, VP23).^d^ We combined the data into a single dataset, totaling 8,590 participants from eight separate studies answering pairs of contrary CTs. A summary description of participants and crucial variables for each study is provided in Table 1.

**Table 1.** Overview of samples and measures used in study 1

		Incoherence		Conspiracism	
Study (place)	*N*	Pairs	Likert	Scale	Likert
LK19s1 (Serbia)	252	5	1–4	CMQ	1–5
PT21s1 (Serbia)	240	3	1–4	CMQ	0–100
PT21s2 (Serbia)	167	3	1–4	CMQ	1–101
PT22s1 (Serbia)	290	7	1–4	CMQ	1–101
VP23s1 (United Kingdom)	796	8	1–5	REJ	8
VP23s2 (Netherlands)	5,261	4	1–5	REJ	4
VP23s3 (United States)	796	8	1–5	REJ	8
VP23s4 (United States)	788	8	1–5	REJ	8
All studies	8,590				

Study (place): Publication and study examined (marked by s1 to s4) with sample description. LK19: (7), PT21: (9), PT22: (8), and VP23: (12). N: sample size. Incoherence: number of incompatible pairs and their Likert range. Conspiracism: the conspiracy scale used, assessed on different Likert response format and operationalized via the CMQ (45), or the proportion of rejections of the official statements (REJ, (12)).

All studies included pairs of contrary CTs, for which participants expressed their agreement using different Likert response formats. For each pair, we rescaled responses from 0 to 1 to express ratings as subjective probabilities (0 =  *strongly disagree*; 1 =  *strongly agree*). Rescaling was applied using the formula $z=\frac{x-\min(L)}{\max(L)-\min(L)}$, where *z* and *x* represent, respectively, the rescaled and original values in the scale *L*, while min(L) and max(L) represent the range of the particular Likert scale *L*. Individual incoherence scores were then computed by averaging the *I* scores across all contrary pairs for each participant, following the equation described in Section SM4. Because we averaged (rather than summed) the scores within participants, the theoretical range of the incoherence index remains constant (0–0.71), thus allowing direct comparisons across studies regardless of the number of pairs administered.

All studies included at least one measure of conspiracism such as the conspiracy mentality questionnaire (CMQ, (45)) or the rejection of the official narrative; see Table 1. We rescaled responses to items on the CMQ to 0–1. The studies in VP23 did not contain an explicit measure of conspiracism (except study 1, which used the conspiracy mentality scale, CMS, (46)). Instead, VP23 asked participants to indicate, for each pair, whether or not they accepted the official version of events after having read a brief description of the official version (e.g. “Do you think that the official reading (i.e. Diana’s death was a tragic accident) is true or false?”). We used the proportion of rejections of the official narrative as a measure of conspiracism (ranging from 0 =  *all official stories accepted* to 1 =  *all official stories rejected*), which correlates positively with the CMS ($r_{794}=0.58,[0.53,0.63]$, P<0.001, in VP23’s study 1, when both measures were available). Note that such a way of operationalizing conspiracism via rejection of the official narrative mirrors the single-item conspiracy scale (47) in which participants, after having read an introductory text about CTs, were asked to express their agreement (on a 9-point Likert) with the statement *the official version of the events given by the authorities very often hides the truth*. Nonetheless, because the average rejection of the official narrative is not an explicit independent assay of conspiratorial thinking, we replicate the analyses dropping these studies—results remain largely unchanged (see Section SM6).

### Results

**Figure 2:**
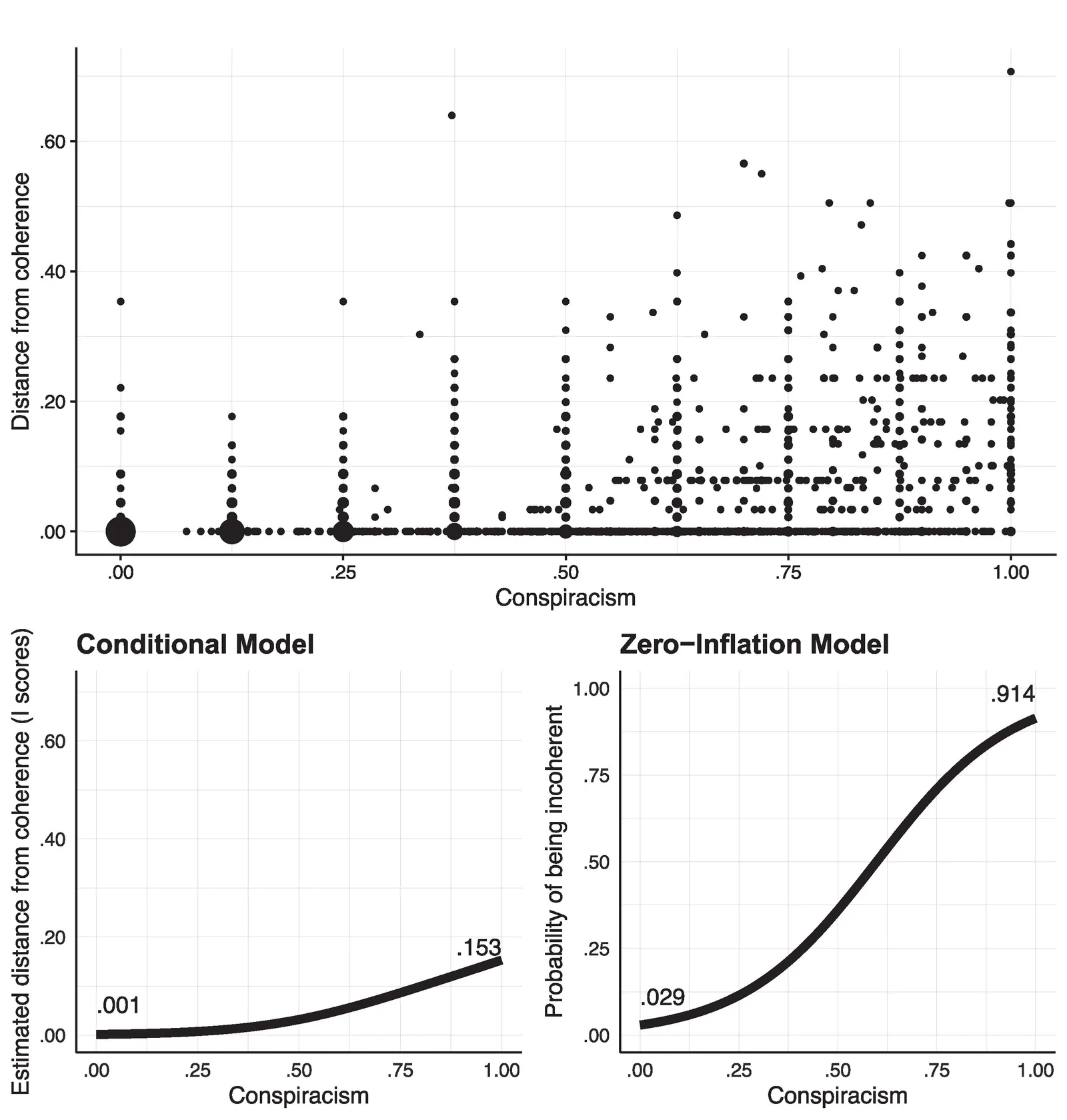
Conspiratorial incoherence by conspiracism. Top panel, size of plotting symbol is proportional to the number of responses. Bottom panel left, fitted regression line from the conditional part of the model predicting the average distance from the coherence boundaries by conspiracism (note the maximum distance from coherence is 0.71). Bottom panel right, fitted regression line from the zero-inflation part of the model predicting the probability of being incoherent (i.e. I>0) by conspiracism.

In Fig. 2, top panel, we show a scatter plot of participants’ distance from the coherence boundary (*I* scores) as a function of conspiracism. We addressed two primary questions: (i) To what extent are incoherence and conspiracism associated? and (ii) What is the prevalence of incoherence among individuals high in conspiracism? To examine these relationships across the 8 studies, we fit a multilevel zero-inflated (ZI) Gamma regression model using the *glmmTMB* package in R (48). ZI models are well-suited for fitting data with a large proportion of zeros (as in our case; 78.7%), allowing for simultaneous inferences about two components: the probability of zero values (the zero-inflation component) and the distribution of nonzero values (the conditional component). We used conspiracism as a predictor in both components of the model to estimate the likelihood of observing excess zeros (i.e. the absence of incoherence; I=0), and to model the departure from the coherence boundary among nonzero cases (i.e. I>0). In both components, we included random intercepts and random slopes by study (see model specifications in Section SM7).

In the conditional part of the model (I>0), conspiracism was associated with incoherence (b=1.28, SE=0.10, z=13.43, P<0.001, OR=3.60), showing that for one unit increase in conspiracism (i.e. from 0 = the strongest nonbelievers to 1 = the strongest believers), the odds of incoherence increase threefold. To facilitate comparison between this odds ratio (OR) and more commonly used effect size measures, we converted the OR to both Pearson’s *r* and Cohen’s *d* (using the R package *effectsize* (49)). The converted OR yielded r=0.33 and d=0.71. Figure 2, panel bottom left, illustrates this effect, showing a rise in predicted incoherence as a function of conspiracism.

The zero-inflation component (probability of I=0, i.e. being coherent) revealed that conspiracism strongly reduced the likelihood of excess zeros—that is coherence—in the data (b=−5.88, SE=0.52, z=−11.40, P<0.001, OR=0.003), indicating that greater conspiracism drastically decreases the odds of coherence. Conversely, the odds of observing incoherence increased sharply, with an OR of 356.91 for a one-unit increase in conspiracism (r=0.85, d=3.24). As shown in Fig. 2, panel bottom right, the predicted probability of being incoherent rose from 3% at the lowest levels of conspiracism to 91% at the highest levels.

For comparability with simpler, more commonly used methods, we also report the model fitted with a Gaussian distribution, which showed that conspiracism predicted incoherence (β=0.65, SE=0.08, P<0.001). Also note that including item pairs from previous research that are not logically incompatible (e.g. those from (20)) produces larger effect sizes, indicating an artificial inflation of the incoherence estimate (see Section SM8).

We estimated the prevalence of incoherence by counting how many participants with high (vs. low; split at the *unsure* midpoint) levels of conspiracism were classified as incoherent (see Section SM9). We found that 58.43% of participants high (vs. low) in conspiracism were incoherent in at least one pair (vs. 11.45%). A similar pattern emerged when subsetting the dataset to VP23’s studies, in which participants indicated, for each event, whether or not they believed the official story (see Table S5). On average, 27.86% (depending on topic, from 5.8 to 66.5%) of participants who rejected the official story endorsed two incompatible CTs exceeding the probability constraints, compared to 9.48% (from 0.71 to 46.34%) of those who accepted the official story. Participants rejecting the official story also showed higher *I* scores (with Cohen’s *d* ranging from 0.26 to 3.16).

Finally, we tested whether the observed incoherence could be attributed to random or careless responding rather than systematic endorsement of incompatible CTs. We conducted a simulation-based test in which, for each item pair, we generated independent responses drawn from the observed response distributions. If incoherence were merely a product of chance, simulated and observed incoherence should not differ. Observed incoherence was significantly higher than simulated incoherence both in the full sample (β=0.39, SE=0.09, t=4.17, P<0.001) and among participants high in conspiracism (β=0.21, SE=0.09, t=2.42, P=0.02). These findings indicate that incoherence reflects systematic above-chance co-endorsement of incompatible CTs rather than noise (see Section SM10 for details).

### Discussion

With our reanalysis of existing data on conspiratorial incoherence, we provided a reliable estimate of incoherence associated with conspiracism, and confirmed that conspiratorial incoherence is a hallmark of conspiracism (1–3).

To our knowledge, only two prior studies have moved beyond correlations between items and have assessed conspiratorial incoherence at the individual level (8, 9), reporting correlations with conspiracy mentality in the range of r=0.33 to r=0.59. Our effect sizes are comparable to those prior findings, although they surpass the strongest known predictors of conspiratorial belief (e.g. social threat: r=0.56, (50); dangerous worldview: r=0.39, (51)), and fall within the upper range of observed correlations between specific CTs and general conspiracy mentality (r=0.37 to r=0.76; (45)). In terms of prevalence, our findings reveal a staggering portion of incoherent conspiracy believers (58.43% vs. nonbelievers 11.45%): the strongest believers have a 91% probability of being incoherent, compared to 3% for the weakest believers.

Given the strength and prevalence of the association between conspiracism and incoherence, an important question is why such incoherence arises. The in/coherence framework proposes that beliefs are endorsed when they align with an overarching stance that *the official narrative is not true* (1, 2, 4). This framework extends beyond conspiratorial thinking to stereotypes (from antisemitism (14) to anti-transgender rhetoric (15)), and mirrors the compartmentalized thinking observed in authoritarian personalities (52). In each case, strong ideological commitment facilitates the acceptance of logically incompatible statements by organizing them under a unifying higher-order belief system. Qualitative evidence suggests that this coexistence of incompatible ideas is maintained by rationalizing strategies (7): participants often focus on the shared thematic core of the two incompatible claims such as distrust of authority while minimizing mutually exclusive details. This aligns with experimental findings that conspiracism reflects a broad openness to alternative explanations rejected by authorities, rather than a commitment to any single narrative (53).

However, an alternative explanation for the observed incoherence has been suggested. A few studies show that conspiracism also correlates with the endorsement of neutral, nonconspiratorial incompatible statements (5, 6, 8, 9), suggesting that the phenomenon may reflect a broader tolerance for inconsistency and reliance on gut feelings rather than purely a worldview-based construction of an illusory coherence (54). Moreover, conspiracy believers are more susceptible to probabilistic reasoning errors (55–57) and have a biased conception of randomness in which coincidences do not exist (58). Thus, when thinking in probabilistic terms during the evaluation of alternative explanatory scenarios for a given event (7, 13), believers may overestimate the likelihood of multiple, mutually incompatible hypotheses, thereby exceeding the coherence boundary. It seems that although higher-order beliefs can account for worldview-specific incoherence, reduced sensitivity to logical incompatibility (54) may explain a more general proneness toward endorsing incompatible statements.

Disentangling these effects, i.e. understanding whether incoherence reflects a domain-specific form of biased reasoning or a broader form of incoherent thinking (54), requires targeted experimental designs. In study 2, we disentangle these effects by manipulating the content domain (worldview-related vs. worldview-neutral) and logical relation (contrary vs. contradictory) of pairs of incompatible statements matched by topic. This design can determine whether incoherence arises primarily from worldview-based incoherent reasoning or from a more general tendency toward incoherence.

## Study 2

In study 2, we test whether incoherence arises primarily from worldview-based incoherent reasoning or from a more general tendency toward incoherence. To do so, we introduce a novel design that addresses several limitations of the studies reanalyzed in study 1.

First, one could argue that participants are incoherent only because they accept (vs. reject) the incompatible claims: the coherence range remaining after committing to one CT is reduced, making it easier for a believer (vs. nonbeliever) to become incoherent. We therefore need to allow incoherence to arise independently of whether participants accept or reject the claims. A potential solution is to introduce contradictory, instead of contrary, pairs. With contradictory pairs, the coherent space is reduced from an area (A+B≤1) to a precise line (A+B=1), so it is no longer relevant whether the departure from coherence stems from acceptance or rejection of both claims. As we explained in Section SM2, the negation of a conspiracy turns the proposition into a nonconspiracy. Therefore, a conspiracy and its negation are incompatible not only in their content (e.g. Princess Diana murdered vs. not murdered) but also in their worldview alignment (conspiracy vs. nonconspiracy). Because these two contradictory statements pull in opposing worldview directions, exceeding the coherence boundary cannot be attributed to worldview-consistent endorsement of both claims, as it does with contrary pairs. Introducing contradictory pairs thus removes the artifact of incoherence arising only from joint acceptance and allows incoherence to be measured when claims are rejected as well.

Second, we introduce incompatible pairs that offer nonconspiratorial (vs. conspiratorial) explanations for socially relevant situations similar to those that have generated CTs. This allows us to test whether conspiracism predicts both conspiratorial and nonconspiratorial incoherence within the same explanatory framework, and it equates the range of incoherent response options for believers and nonbelievers.

Third, endorsement of incompatible claims may also reflect prior exposure and knowledge rather than reasoning. It is very likely that believers have been exposed to a wide range of CTs, including mutually incompatible ones for the same event (2). Repeated exposure to such claims can increase familiarity and enhance perceived accuracy (59, 60). Participants may therefore agree with what they find familiar rather than with what they believe to be the most likely explanation for a given event (e.g. the death of Princess Diana, 9/11, COVID). We therefore designed fictitious events along with incompatible explanations, exposing participants to claims they could not possibly have encountered previously. This allows us to extract incoherent reasoning disentangled from knowledge and prior exposure.

Finally, in study 1, it was necessary to convert participants’ Likert-scale agreements into subjective probabilities in order to address probabilistic coherence within the Bayesian framework. To exclude the possibility that our results are an artifact of this conversion from coarse approximations to probabilities, we pursued a more direct approach by explicitly asking participants to provide probability estimates of each claim being true or false.

In summary, we created vignettes depicting fictitious events and provided potential explanations that vary by type (conspiracy vs. nonconspiracy) and logical relation (contrary vs. contradictory). We then measured participants’ explicit probability judgments on each claim.

### Methods

The study obtained ethical approval from the University of Bristol (ref: 29828) and the survey was preregistered (https://aspredicted.org/uy4ms3.pdf). A total of 525 US-based, English-fluent Prolific users provided informed consent and initiated the survey (see Section SM16). We excluded 5 participants who failed an attention check, 38 who left blank three or more incompatible items, and 13 who were flagged as potential bots. The final sample included 469 participants (age: M=43.05, SD=13.52, range: 19–84; 242 women, 219 men, and 8 nonbinary/undisclosed).

To measure incoherence, we created 16 vignettes describing fictitious but realistic events set in Europe (see list of vignettes and pairs in Section SM11). Europe was chosen to make the vignettes realistic while situating them in a setting sufficiently unfamiliar to US-American participants to limit the influence of prior knowledge. We avoided potential political influence beyond claims’ non/conspiracy content, e.g. by excluding mentions of climate change (61).

For each vignette, we created four pairs of incompatible statements by crossing explanation type (conspiracy vs. nonconspiracy) and logical relation (contrary vs. contradictory), obtaining four conditions: conspiracy contrary, conspiracy contradictory, nonconspiracy contrary, and nonconspiracy contradictory. To make the distinction between conspiracy and nonconspiracy explanations more salient, conspiracy explanations involved socially relevant deception, whereas nonconspiracy explanations described accidental causes. We ensured that the propositional content and its negation (for contradictory claims) were unambiguous and salient by prefacing each claim with its true/false status (see example in Table 2). Each participant saw four randomized vignettes per condition, with only one incompatible pair presented as explanations for each vignette; the order of claims within each pair was also randomized (an example of how participants viewed the vignette is shown in Fig. S4).

**Table 2.** Example of vignette and incompatible pairs used in study 2.

*Chloe is an 8-year-old girl and a promising young skier. Her father, an influential member of the European Parliament, often takes her skiing in the Swiss Alps. One day, Chloe separated from her group during ski school and did not return home. Her father asked the European Union for funds to support a search for her, triggering negative reactions from colleagues and opponents. After three days, Chloe returned home.*	
CTCD	It is true that Chloe has been secretly held by her father in order to misuse public funds It is false that Chloe has been secretly held by her father in order to misuse public funds
CTCR	It is true that Chloe has been secretly held by her father in order to misuse public funds It is true that Chloe escaped an environment in which she was secretly forced into child labor
NCCD	It is true that Chloe accidentally fell from a nearby cliff It is false that Chloe accidentally fell from a nearby cliff
NCCR	It is true that Chloe accidentally fell from a nearby cliff It is true that Chloe was caught in a sudden avalanche

CTCD, conspiracy contradictory; CTCR, conspiracy contrary; NCCD, nonconspiracy contradictory; NCCR, nonconspiracy contrary.

For each vignette, participants rated the likelihood of the two logically incompatible explanations, which were presented together below the vignette, using a 0–100 probability scale (0 = certainly false, 100 = certainly true). The chosen probability value was always visible so that participants could calibrate their judgments. Incoherence was computed as the Euclidean distance to the coherence boundary. Given two incompatible statements (A and B), contrary incoherence is computed as the positive distance from 1 (see Section SM4), whereas contradictory incoherence is computed as any deviation from A+B=1, as shown in Equation S3. Conspiracism was measured with the five-item CMQ (45).

### Results

We computed 7,479 *I* scores (25 pairs were excluded because only one estimate was provided) from 469 participants. On average, endorsement of explanations was mostly worldview-consistent: conspiracism correlated positively with conspiracy explanations framed as true and negatively with nonconspiracy explanations framed as true; the reverse pattern was found when explanations were framed as false (see Section SM12).

We preregistered two research questions: RQ1: Does incoherence generalize to nonconspiracy content? RQ2: Does incoherence generalize to contradictory pairs? To address each question, we fitted two separate ZI Gamma models predicting incoherence from conspiracism (CMQ, (45)), with a random intercept for participants, restricting the data to the relevant subset (nonconspiracy pairs for RQ1; contradictory pairs for RQ2). Conspiracism predicted the likelihood of being incoherent in nonconspiracy pairs (b=0.93[0.32,1.54], P=0.003), but not its degree (b=−0.22[−0.54,0.10], P=0.185). The same pattern emerged for contradictory pairs: conspiracism predicted the likelihood of incoherence (b=2.77 [1.46, 4.08], P<0.001), but not its degree (b=0.16[−0.23,0.54], P=0.418). These results indicate that the association between conspiracism and incoherence generalizes both to nonconspiracy content and to contradictory pairs, but is confined to whether incoherence occurs rather than to its magnitude once it does.

To examine whether conspiracism was differently associated with incoherence depending on explanation type (conspiracy vs. nonconspiracy) and logical relation (contrary vs. contradictory), we fitted two additional models on the full dataset, each including an interaction between conspiracism and the relevant factor. The interaction between conspiracism and explanation type was significant in both model components (conditional: b=0.56[0.22,0.91], P=0.001; zero-inflation: b=1.28 [0.75, 1.80], P<0.001): conspiracism predicted both the degree and the likelihood of incoherence for conspiracy pairs (conditional: b=0.37 [0.01, 0.73], *P* = 0.047; zero-inflation: b=2.27 [1.59, 2.94], P<0.001), whereas for nonconspiracy pairs it predicted only the likelihood (zero-inflation: b=0.99[0.33,1.65], P=0.003) but not the degree (conditional: b=−0.20[−0.53,0.14], P=0.25). By contrast, the interaction between conspiracism and logical relation was not significant in either component (conditional: b=0.26[−0.13,0.65], P=0.195; zero-inflation: b=0.26[−0.36,0.88], P=0.415): conspiracism predicted the likelihood of incoherence for both contrary (b=1.83 [0.99, 2.67], P<0.001) and contradictory (b=2.09 [1.28, 2.89], P<0.001) pairs, but did not predict its degree in either case (contrary: b=−0.15[−0.56,0.26], P=0.466; contradictory: b=0.11[−0.23,0.44], *P* = 0.529). Taken together, these results suggest that the pattern of incoherence depends more on explanation type than on logical relation.

**Figure 3:**
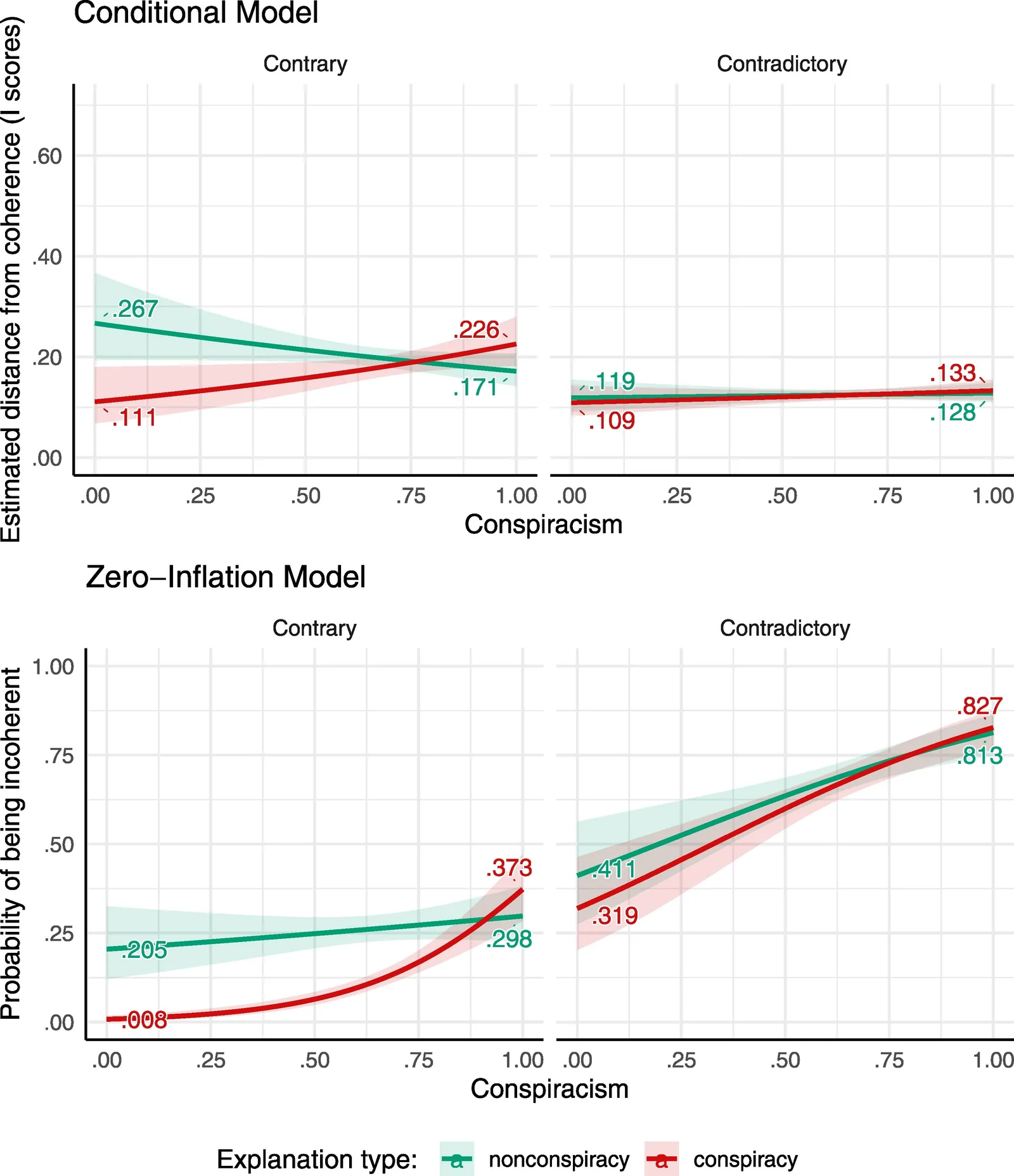
Incoherence by conspiracism (CMQ (45)) by logical relations (contrary vs. contradictory) and explanation types (conspiracy vs. nonconspiracy). Top panels: fitted regression lines from the conditional part of the model predicting the average distance from the coherence boundaries by conspiracism. Note the maximum distance from coherence is 0.71. Bottom panels: fitted regression lines from the zero-inflation part of the model predicting the probability of being incoherent by conspiracism.

However, the theoretical distinction between contrary and contradictory pairs also concerns worldview content: contrary pairs allow worldview-consistent joint endorsement of both claims, whereas contradictory pairs neutralize the worldview against itself. To fully disentangle these mechanisms and obtain condition-specific estimates, we fitted a single model including a three-way interaction between conspiracism, explanation type, and logical relation. Figure 3 displays the fitted lines for the estimated magnitude of incoherence (conditional component) and the probability of being incoherent (zero-inflation component, sign-reversed) as a function of conspiracism, separately for each combination of explanation type and logical relation (full model details and individual slopes for each condition are reported in Table S9; results do not diverge from the preregistered two-model specification, reported in Table S8 for transparency; see also Section SM13).

Conspiracism predicted incoherence most widely in the conspiracy-contrary condition, where it was associated with both the degree (b=0.71[0.05,1.37], P=0.034) and the likelihood (b=4.32[3.22,5.41], P<0.001) of incoherence, replicating the core finding of study 1. In the conspiracy-contradictory condition, conspiracism predicted the likelihood of incoherence (b=2.32[1.42,3.23], P<0.001) but not its degree (b=0.20[−0.20,0.59], P=0.323). The same pattern held for the nonconspiracy-contradictory condition: conspiracism predicted the likelihood (b=1.83 [0.92, 2.73], P<0.001) but not the degree (b=0.07[−0.31,0.45], P=0.704) of incoherence. Conspiracism did not predict incoherence in the nonconspiracy-contrary condition, neither in terms of likelihood (b=0.50[−0.42,1.42], P=0.288) nor degree (b=−0.44[−0.91,0.02], P=0.063).

To rule out the possibility that contradictory incoherence is merely an artifact of response noise—whereby participants fail to compute the sum of contradictory pairs as exactly 1—we recomputed contradictory incoherence by widening the coherence band by ±5%, treating as coherent any response within 0.95<A+B<1.05. Results were virtually unchanged (see Table S10).

To contextualize these results within previous investigations, we analyzed the subset of contrary-conspiracy pairs from study 2 and compared effect sizes and distributions with those from study 1 via a mini meta-analysis across all nine samples. Study 2 exhibited the largest conditional effect among all samples (b=1.00 vs. pooled effect: *b* = 0.36 [0.22, 0.49], z=5.10, *P* < 0.001), yet a comparatively moderate zero-inflation effect (b=4.35 vs. pooled effect: b=5.17, [4.38, 5.96], z=12.80, P<0.001). In other words, many participants were just barely incoherent, but those high (vs. low) in conspiracism showed larger deviations from coherence. Several design features of study 2—a continuous probability scale, an explanation-based vignette format, fictitious scenarios, and explicit logical framing—likely raised sensitivity to detect any departure from coherence irrespective of conspiracism level, while allowing conspiracism to emerge as a stronger driver of the magnitude of incoherence (see Section SM15 for details).

### Discussion

Study 2 replicated and extended the findings of study 1 using an explanation-based vignette format with fictitious scenarios, explicit logical framing, and subjective probability responses on a continuous scale. In general, conspiracism predicted incoherence for both nonconspiracy and contradictory pairs, but with condition-specific patterns. Specifically, conspiracism predicted both the likelihood and the degree of incoherence for conspiracy-contrary pairs, but neither for nonconspiracy-contrary pairs. For contradictory pairs, regardless of explanation type, conspiracism predicted the likelihood of incoherence but not its degree.

To account for this pattern, we propose that the link found between incoherence and conspiracism reflects two components: a general component (reduced logical monitoring which is revealed by the contradictory pairs) and a content-specific component (worldview-driven endorsement which is observable with the contrary pairs).

Contradictory pairs impose a strict constraint (A+B=1). Satisfying this constraint is a metacognitive task that requires recognizing that the two statements are logically linked and that responses must be calibrated accordingly. Our results show that the likelihood of violating this constraint (even when it is relaxed by ±5%) increased with conspiracism regardless of explanation type, suggesting that individuals high in conspiracism may be less likely to engage in such coordination because of a broader tendency toward less analytically vigilant thinking (51, 62). Once someone has crossed the incoherence threshold, failing to strictly enforce A+B=1, the magnitude of this deviation was not systematically driven by conspiracism. Although puzzling at first glance, this is readily explainable if one recalls that contradictory pairs neutralize the worldview against itself: for a conspiracy believer, endorsing the conspiratorial claim means rejecting its nonconspiratorial negation, and vice versa. Unlike contrary pairs, where the worldview pushes endorsement of both claims in the same direction, contradictory pairs offer no motivational basis for the degree of deviation to increase with conspiracism, consistent with the nonsignificant conditional slopes for both conspiracy and nonconspiracy contradictory pairs.

The content-specific component, by contrast, emerges with contrary pairs that are conspiratorial. Unlike contradictory pairs, which make monitoring failure detectable without motivational factors, for contrary pairs, both explanations are consistent with the conspiratorial worldview, which pushes endorsement of both claims simultaneously (instead of neutralizing each other). This unidirectional worldview-driven push inflates both probabilities and drives their sum beyond the coherence boundary. However, this component operates only when the incoherent content aligns with the believer’s conspiratorial worldview. When the statements are nonconspiratorial, there is no motivation to engage in incoherent responding.

## General discussion

In this article, we identified the conceptual, methodological, and statistical limitations that have led to incompatible interpretations in the existing literature on conspiratorial incoherence. We demonstrated that previous assessments based on correlations between responses to incompatible items are inadequate for determining the presence (or absence) of incoherence, a conclusion that supports a previous critique (12).

We introduced a framework from a probabilistic extension of classical logic to resolve terminological and conceptual ambiguities surrounding logical incompatibility: unless each pair is first verified as truly incompatible, estimates of incoherence risk being inflated by responses to pairs that are compatible, or only superficially opposed. We therefore introduced our *I* score based on probabilistic coherence and showed that, compared to previous approaches, it is more sensitive, shows better psychometric properties, and provides superior predictive validity.

In study 1, reanalyzing previous data, we provided a reliable estimate of conspiratorial incoherence associated with conspiracism, and confirmed that conspiratorial incoherence is a hallmark of conspiracism (1–3). Recent conclusions to the contrary (12) were based on analyses that we have shown to be nondiagnostic. With the simulation-based test (see Section SM10), we show that incoherence reflects systematic above-chance co-endorsement of incompatible CTs rather than noise. In study 2, we extended this work using a novel design to disentangle whether incoherence arises primarily from worldview-based incoherent reasoning or from a more general tendency toward incoherence. We found evidence for both, and propose a two-component account: a general component, associated with reduced logical monitoring, that emerges in contradictory pairs and supports a *general proneness to incoherence*; and a content-specific component, associated with worldview-driven endorsement, that emerges in contrary conspiracy pairs and supports *selective conspiratorial incoherence*.

A related two-component dissociation was reported by Pennycook and Rand (63) in the domain of fake news and partisan misinformation: susceptibility to fake news was better explained by a failure to engage analytical thinking than by the use of analytical thinking in service of partisan goals. While the content domain and task differ, the conceptual architecture is parallel. Our results, however, diverge somewhat: we found evidence for both components, and, if anything, the worldview-driven content-specific component (contrary-conspiracy condition) yielded on average stronger effects than the general reduced-logical-monitoring component (contradictory-conspiracy condition).

We need to stress the broader relevance of this line of research. An accumulation of mutually reinforcing conspiratorial ideas may have cumulative and cascading psychological effects (64). Longitudinal evidence shows not only that conspiracism predicts belief in specific CTs (65, 66) but also that the reverse is true: endorsing specific CTs can increase general conspiracism (65, 67) and belief in additional CTs over time (67–69). In the minds of believers, multiple alternative explanations for major events compete against a single official narrative, so they cannot all be false (70), particularly when they align with the believer’s worldview (71). These narratives provide a shared set of assumptions (72) and can create an inflated sense of global coherence (73), thereby reinforcing the overarching conspiratorial worldview.

Recent work provides some guidance for developing interventions. Petrović and Žeželj (54) experimentally tested several approaches to reduce endorsement of incompatible statements. These included training participants to detect inconsistencies, prompting them to reconcile incompatible claims, and explicitly confronting them with their own inconsistent beliefs. None of these reduced endorsement of incompatible views, largely because participants rationalized or reframed their incompatibility rather than rejecting them. Inoculation interventions that explicitly train recognition of incoherent arguments as a manipulation technique reliably increase people’s ability to detect incoherence (74), indicating that the problem is not a basic inability to grasp inconsistency. Nevertheless, Morgenroth et al. (15) found that opponents of trans-inclusive policies continued to endorse incompatible anti-transgender arguments even when the incompatibility was made salient, although incoherence was slightly reduced if participants had first rated the level of incompatibility of the two statements. Together, these findings suggest that simply highlighting mutual incompatibility, or improving people’s ability to detect it, may not suffice to reduce endorsement.

Increasing saliency of incompatibility, such as asking agreement with a single statement providing two incompatible arguments even if participants endorse both statements when presented separately (e.g. asking “Do you believe Osama bin Laden is both dead and alive?” (11), p. 65) could not only inform about the limits of compartmentalization (52), but could also represent a potential intervention aimed at reducing their mutual endorsement by making the incoherence more blatantly explicit. Alternatively, artificial intelligence, which has shown encouraging results in reducing belief in CTs (75), could be used to deliver scalable and tailored interventions that train users in probability theory and recognition of incoherence, and that explore whether worldview-related incompatible beliefs can be reconciled once the underlying incompatibility is made explicit.

In study 1, when we estimated the prevalence of incoherence in VP23’s studies across specific events, we found that, on average, 9.48% of participants endorsed two incompatible CTs *and* the official story. Although they represent a minority, this finding warrants further scrutiny. What are the implications of incoherence arising from the simultaneous endorsement of these opposing views? Does this challenge the idea that CTs are typically endorsed in opposition to official accounts (53)? In our study 2, endorsement of explanations was largely worldview-consistent: conspiracism was associated with acceptance of conspiratorial explanations *and*, to a lesser extent, with rejection of mundane ones. This asymmetry shows that even among individuals high in conspiracism, the official story is not necessarily rejected outright; instead, the probability space is redistributed so that conspiratorial possibilities receive more weight than coherence permits.

The key insight from our framework is that incoherence is not an all-or-nothing phenomenon. Modeling conspiracy beliefs as probabilities makes it possible to represent the coexistence of opposing (world)views within rationality boundaries with far greater precision than an atomic true/false framework. A person may assign 80% probability to the official story and distribute the remaining 20% uncertainty across alternative scenarios, including contrary and contradictory views, without violating coherence. Within the probability framework, the simultaneous endorsement of CTs and the official account may reflect residual uncertainty allocated to unlikely but psychologically salient alternatives. Motivated conspiratorial incoherence emerges gradually as the motivational component leads believers to overweight worldview-consistent hypotheses, and it becomes detectable precisely because the probabilistic framework provides a principled threshold—the coherence boundary—against which to evaluate belief distributions.

## Limitations and future work

Study 2 introduced several methodological innovations and conservative design features to increase methodological rigor and facilitate cognitive monitoring. However, because these features were introduced simultaneously, their individual contributions to the observed effects remain to be disentangled. We did not systematically pretest whether each design feature, taken individually, affected mutual endorsement and thus incoherence. Paired experimental tests isolating each factor are a fruitful avenue for further research.

We introduced an explanatory framing: rather than rating isolated “factual” conspiratorial claims as in prior studies, participants evaluated explanations embedded within vignettes. This mode is arguably better aligned with the epistemic function of conspiracist cognition, which focuses on providing causal accounts for ambiguous events (76, 77). Consistent with this reasoning, our contrary conspiracy pairs yielded the strongest conditional relationship with conspiracism in the mini meta-analysis. However, because this framing was not tested in isolation, it remains unclear whether the explanatory mode itself facilitates endorsement or whether this effect is driven by other design features. Manipulating explanatory frameworks against factual claims would help clarify the epistemic motives driving reliance on CTs, and extending this comparison to other worldviews would clarify whether the framing advantage is specific to conspiracist cognition or generalizes to other forms of worldview-motivated incoherence (e.g. anti-transgender rhetoric, antisemitism, political polarization, pseudoscience).

A second limitation concerns the ecological validity of our contrary pairs. For each vignette, we tested contrary explanations that were either both conspiratorial or both nonconspiratorial. Although we documented a degree of rejection of nonconspiratorial explanations, we did not present conspiratorial and nonconspiratorial explanations concurrently for the same scenario. Future work could administer all four explanations—two conspiratorial and two nonconspiratorial—simultaneously, which would better mimic real-world exposure to competing worldview-aligned accounts of the same event. Although we expect a clear preference for conspiratorial explanations as a function of conspiracism, a more interesting question is whether the number of available explanations affects incoherence: do conspiracy believers endorse all of them, and does this pattern persist when participants are explicitly asked for probability ratings?

Third, by using fictitious scenarios, we isolated the contribution of conspiracist mentality from that of prior exposure and familiarity with circulating CTs (e.g. death of Princess Diana, 9/11). This separation is a strength, but it also leaves open the question of how mentality and knowledge interact. Pairing fictitious with real-world CTs within the same design could help disentangle these contributions. Further experimental work could also manipulate exposure to fictitious CTs—for instance, through repeated presentation—to test whether the familiarity effect (59, 60) generalizes to novel conspiratorial content.

Finally, we introduced several conservative design features intended to facilitate cognitive monitoring and so reduce the probability of incoherent responses. These included explicitly and unambiguously prefacing claims with “It is true/false that…”, collecting probability ratings and thus activating a numerical mode of responding, keeping the chosen probability value visible to facilitate calibration, and displaying both incompatible claims simultaneously below the vignette. Despite these safeguards, we found a robust association between incoherence and conspiracism, strengthening our core finding. However, testing each feature individually would help identify precisely where monitoring failure occurs in association with conspiracism, whether at the level of recognizing logical incompatibility, calibrating numerical estimates, or integrating information across competing claims.

## Conclusions

We conclude by encouraging researchers to rely on diagnostic, valid, and sensitive measures if their goal is to study departures from normative reasoning. Extending this framework across ideological domains will help clarify whether incoherence arises from motivated reasoning, faulty cognition, or both and whether interventions can reduce incoherence in both worldview-related domains and in general. If incoherence is not merely irrational cognition but a functional feature of ideological worldviews, then understanding it can shed light on how belief systems are maintained and protected from revision. A better understanding of these mechanisms is essential for addressing epistemic fragmentation and strengthening public resilience to misinformation, particularly when ideological commitments override rational epistemic considerations and democratic processes are at stake (78, 79).

## Supplementary Material

pgag250_Supplementary_Data

## Data Availability

The dataset and the R scripts for reproducing the analyses is available at https://osf.io/rtw7b/.

## Notes

a Note that unless proven true or false, CTs are all fictitious. By *fictitious* the authors of these studies mean that CTs were fabricated by researchers to expose participants to novel CTs they could not possibly have encountered previously.

b This point was raised by van Prooijen et al. (12), but note that a single observation cannot establish a positive correlation, since there is no variance in the two variables and at least two different data points for each variable are needed to compute a correlation.

c By contrast, for contradictory statements, a pizza unit can be divided only into two slices (although one of the two slices can be zero), because one of the two statements must be true and the other false.

d Note that in a previous preprinted version of this manuscript, we also included the study by Imhoff and Lamberty (20). However, none of the item pairs in that study represent actual incompatibilities, and thus it has been excluded from the present analysis. For completeness, the item pairs from that publication are still listed in Table S1, and results from analyses using those items are reported in Table S3.

## Previous presentation

These results were previously presented in June 2024 at the CONSPIRACY_FX conference on the consequences of conspiracy theories (Kent, United Kingdom).

## Preprints

A preprint of this article is published at https://osf.io/preprints/psyarxiv/b9er2_v1.
