# Supplementary material for: Still very much dead and alive: Incoherence in conspiracism as a departure from Bayesian rationality
Source: PNAS Nexus. 2026 Aug 18;5(8):pgag250. doi: 10.1093/pnasnexus/pgag250 (PMC13482986; doi:10.1093/pnasnexus/pgag250)
Supplement: Supplementary file 1 [file pgag250_supplementary_data.pdf]

SUPPLEMENTAL MATERIAL (SM)

Still Very Much Dead and Alive: Incoherence in Conspiracism as a Departure from Bayesian Rationality

Alessandro Miani, Nicole Cruz, and Stephan Lewandowsky

SM1. Correlation is not diagnostic of incoherence

Figure S1 (panels A to C) shows hypothetical responses to two incompatible CTs that exhibit different correlation coefficients and their relationship with incoherence. In each panel, the red region identifies unambiguously incoherent responses; that is those that represent endorsement of both incompatible items (*Diana faked her own death* and *Diana killed by MI6*). In **panel A**, when responses are equally spread across the n-point Likert scale, incoherent responses are more likely when correlations are positive (as opposed to negative). It is this observation that justified the operationalization by Wood et al. [1]. In **panel B**, a correlation coefficient of +1 could indicate very high or very low incoherence, depending on whether participants' responses tend to concentrate at the agreement or disagreement endpoint of the scale for both incompatible items. It is this observation that supported the argument by van Prooijen et al. [2] against the presence of incoherence. In **panel C**, a coefficient of around zero can yield different levels of incoherence, depending on whether or not participants overwhelmingly agree with one of the two incompatible items.

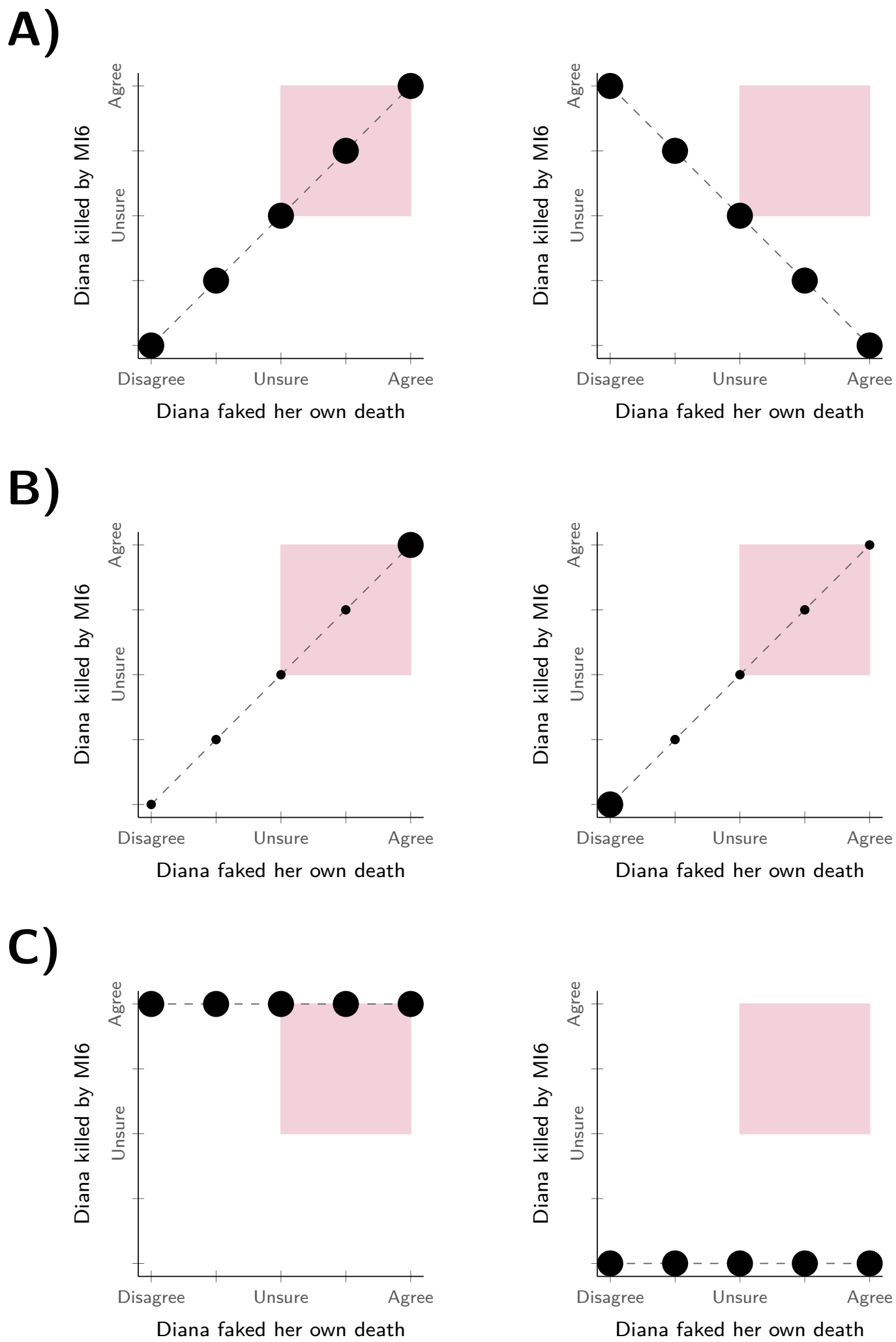

Figure S1. Associations between participants' hypothetical responses and incoherence. Incoherent responses, that is, responses representing agreement to two logically incompatible conspiracy theories, are color-coded in red. In each panel, the dots represent responses to the incompatible items across participants (each dot represents observations from several participants). In panel B, dots' size is proportional to the number of responses.

SM2. Why not contradictory conspiracy theories

As we outlined in the main text, it is not possible to formulate statements that are both genuinely contradictory and conspiratorial in nature. To further clarify the distinction between contradiction and contrariety, let us go beyond CTs. Two statements are contradictory only if they cannot both be true and cannot both be false. Unlike contraries (which can both be false), contradictions are exhaustive because they include all possibilities. For example, the statements *x is a triangle* vs *x is a square* are contraries because they cannot both be true, but they can both be false (e.g., *x is a circle*). Only when one of them is paired with its explicit negation do we obtain a genuine contradiction, as in *x is a triangle* vs *x is not a triangle*. These logical relations are further shown in Figure S2. Note that the case of *dead/alive* works because we explicitly define *alive* as the logical complement of *dead*, that is, *x is alive* ↔ *x is not dead*. In theory, if one day a new state of existence were to be discovered, *dead* vs *alive* would cease to be contradictory; but *dead* vs *not dead* would clearly remain so.

As a result, conspiratorial claims cannot form contradictory pairs. Either we create a negation of a conspiracy (*Diana killed by MI6* vs *Diana* ***not*** *killed by MI6*), but the result is not a conspiracy, or we create an alternative conspiratorial version of the statement (*Diana killed by CIA*), but the result is a contrary statement.

SM3. List of in/compatible pairs used in Study 1

In Table S1, we provide an assessment of the pairs used in the literature [2–6], following the distinction outlined in the main text between *compatible*, *contrary*, and *contradictory* types of statements. We show that none of the pairs represent true contradictions. Most pairs are in fact contraries, and some are even compatible. For instance, a pair from van Prooijen et al. [2], appears at first glance to be contradictory (A = *Although Al Qaeda carried out the attacks, the US government knew they were coming and deliberately did nothing to prevent them* and B = *Al Qaeda did NOT carry out the attacks; the US government planned and executed the attacks entirely, and then blamed them on Al Qaeda*) if one considers only the initial clauses (*Al Qaeda carried out the attacks* vs *Al Qaeda did NOT carry out the attacks*). However, when the full content of both statements is taken into account, it becomes clear that both can be false (e.g., if Al Qaeda carried out the attacks and the US government had no prior knowledge of them).

Table S1: List of pairs assessed on their type of incompatibility, namely **Contrary** and **Compatible**

**Pair**	**Study**	**Classification**	**Our reasoning**
– Although Al Qaeda carried out the attacks, the US government knew they were coming and deliberately did nothing to prevent them<br>– Al Qaeda did NOT carry out the attacks; the US government planned and carried out the attacks entirely, and then blamed them on Al Qaeda	VP23s1<br>VP23s3<br>VP23s4	**Contrary**	It cannot both be true that (a) Al Qaeda carried out the attacks with no resistance from the US government, and that (b) the US government carried out the attacks themselves. But both could be false, e.g. if the attacks were carried out by Al Qaeda despite resistance from the US government.
– America planted the coronavirus in China to weaken their economic growth, but that backfired on them<br>– China clearly made the virus in order to establish world domination	PT22s1<br>PT22s2	**Contrary**	It cannot both be true that (a) America brought the virus to China and that (b) the virus was made in China. But both could be false if the virus originated in some other place.
– Climate change exists, but it is due to the earth's natural cycle and people have very little influence on this. Science has been exaggerating the extremity of climate change and is profiting off of government investments and subsidies for climate change prevention<br>– Climate change doesn't exist, the earth has been getting colder, not warmer. The government is pretending [are lying] climate change exists, in order to get money from consumers through taxes	VP23s1<br>VP23s2<br>VP23s3	**Contrary**	It cannot both be true that (a) climate change exists in the form of earth's natural temperature cycles and (b) climate change does not exist and the temperature is getting colder. But both could be false if anthropogenic climate change exists that is making the climate warmer.
– Climate change is really happening, but people have very little influence on this. Scientists and governments deliberately exaggerate the role of human activity as cause of climate change<br>– Climate change is NOT really happening. Scientists and governments are lying that climate change exists	VP23s4	**Contrary**	It cannot both be true that (a) climate change is real but humans play little role and that (b) climate change is not real. But both could be false if climate change is real and human-driven
– The coronavirus is a dangerous bioweapon created by humans in a laboratory<br>– The coronavirus is not dangerous or man-made; it is a harmless flu virus heavily exaggerated by powerful elites to oppress the people	VP23s2	**Contrary**	It cannot both be true that coronavirus is (a) dangerous and human-made and (b) harmless and not human-made. But both could be false if coronavirus is dangerous and with zoonothic origins
– COVID-19 vaccines contain only saline solution<br>– The pharmaceutical industry is well aware of the dangers of COVID-19 vaccines and yet insists on distributing them	PT22s1<br>PT22s2	**Contrary**	It cannot both be true that (a) Covid vaccines contain only saline and therefore have no eect on health, and that (b) Covid vaccines are dangerous to health. But both could be false if Covid vaccines contain active ingredients and are of net benet to health.
– Diana faked her own death so that she and Dodi could retreat in isolation<br>– Diana was [had to be] killed because the British government could not accept that the mother of the future king was involved with a Muslim Arab	VP23s1<br>VP23s4	**Contrary**	That (a) Diana faked her own death (at some time x) implies she was still alive afterwards. This is incompatible with (b) Diana having been killed (at time x). But both could be false if e.g. she died (at time x) by accident.
– Doctors are paid to supposedly treat the infected, and yet all the hospitals are empty<br>– The fact that the hospitals are so overcrowded that patients sleep in toilets is being hidden from the public	PT22s1<br>PT22s2	**Contrary**	It cannot both be true that the hospitals are (a) empty and (b) overcrowded. But both could be false if the hospitals are at medium capacity.
– Elvis Presley did not die in 1977. He faked his own death to escape his life of fame<br>– Elvis Presley did die in 1977, but not of a heart attack. He was murdered	VP23s3	**Contrary**	It cannot both be true that (a) Elvis did not die in 1977 and that (b) Elvis died by murder in 1977. But both could be false if Elvis died from some other cause in 1977.

(continued)

Pair	Study	Classification	Our reasoning
– Epstein is not dead but bought his way out of prison. His alleged suicide was staged, allowing him to avoid legal prosecution and gain back his freedom<br>– Epstein was murdered to prevent him from testifying against important and influential people who participated in his sex crimes	VP23s1<br>VP23s3<br>VP23s4	**Contrary**	It cannot both be true that (a) Epstein is still alive and that (b) Epstein was murdered. But both could be false if Epstein died from some other cause.
– Hitler didn't die, he fled to Argentina with his wife Eva Braun. The remains found by the Russians were not those of Hitler and Braun<br>– Hitler died, but he did not commit suicide. He was killed by the Russians, who covered up the murder as a suicide	VP23s1<br>VP23s2<br>VP23s3<br>VP23s4	**Contrary**	It cannot both be true that (a) Hitler didn't die at the end of WWII and that (b) he was killed by the Russians at the end of WWII. But both could be false if Hitler died of other causes at the end of WWII.
– HIV is deliberately made in laboratories of Central Africa<br>– HIV is deliberately made in the USA laboratories to reduce the percentage of black population in Africa	LK19s1<br>PT21s1<br>PT21s2	**Contrary**	Assuming the statements presuppose a single origin, it cannot both be true that HIV was (a) made in the USA and (b) made in Africa. But both can be false if HIV originated somewhere else.
– HIV is deliberately made in the laboratories of European pharmaceutical corporations to reduce African population and thus to control the flow of immigrants from Africa into Europe<br>– HIV is deliberately made in laboratories of Central Africa	LK19s1	**Contrary**	Assuming the statements presuppose a single origin, it cannot both be true that HIV was (a) made in Europe and (b) made in Africa. But both can be false if HIV originated somewhere else.
– HIV is deliberately made in the USA laboratories to reduce the percentage of black population in Africa<br>– HIV is deliberately made in the laboratories of European pharmaceutical corporations to reduce African population and thus to control the flow of immigrants from Africa into Europe	LK19s1	**Contrary**	Assuming the statements presuppose a single origin, it cannot both be true that HIV was (a) made in the USA and (b) made in Europe. But both can be false if HIV originated somewhere else.
– It is clear that masks have no effect on health whatsoever, but are still imposed on us for profit<br>– Wearing a mask leads to carbon dioxide intake, which damages the lungs – more work for the pharmaceutical industry!	PT22s1<br>PT22s2	**Contrary**	It cannot both be true that (a) masks have no effect on health and (b) masks are damaging to health. But both could be false if masks are of net benefit to health.
– It's no wonder that after decades of dangerous experiments the virus finally escaped from the lab in 2019 – no one believes that it was accidentally transferred from animals<br>– This coronavirus has been circling in the human population for years, but was just now conveniently revealed	PT22s1<br>PT22s2	**Compatible**	It can (in principle) both be true that (a) the virus was created in a lab then escaped in 2019, and that (b) the virus was circulating in humans for years before it was revealed. The two statements do not constrain one another's truth values.
– Josip Broz Tito was an American spy<br>– Tito was a Russian spy	LK19s1<br>PT21s1<br>PT21s2	**Compatible**	It can (in principle) both be true that (a) Tito was an American spy and that (b) he was a Russian spy namely if he was a double agent. The statements do not constrain one another's truth values. But one could argue that double agents are rare compared to single agents, rendering the joint truth of the two statements unlikely.
– Pharmaceutical companies already have found a cure for cancer years ago; they are deliberately withholding it from the public in order to continue selling the more profitable chemo therapies [Various pharmaceutical companies have found a cure for cancer years ago, but they are deliberately withholding it from the public]<br>– Pharmaceutical companies are still nowhere near finding a cure for cancer; they are deliberately obstructing and slowing down medical research on this, in order to continue selling the more profitable chemo therapies [No pharmaceutical company has ever found a cure for cancer because they never actually tried to find one]	VP23s1<br>VP23s3<br>VP23s4	**Contrary**	It cannot both be true that pharmaceutical companies (a) found a cure for cancer long ago, and (b) have not yet found a cure for cancer and are still far from that goal. But both could be false if the companies are making signicant progress towards nding a cure and are now close to reaching that goal.
– Slobodan Miloevi did not die by natural death, but was murdered in the Hague<br>– Slobodan Miloevi did not die in the Hague, but faked his death	LK19s1<br>PT21s1<br>PT21s2	**Contrary**	It cannot both be true that (a) Miloevi was murdered at time x and (b) faked his death at time x. But both could be false if Miloevi's life took some other path at time x.
– The coronavirus is a powerful bioweapon made for the mass depopulation of the planet<br>– COVID-19 is nothing more than a common cold whose consequences are intentionally exaggerated	PT22s1<br>PT22s2	**Contrary**	It cannot both be true that Covid is (a) an engineered dangerous bioweapon and (b) a common cold. But both could be false if Covid is non-engineered and more dangerous than the common cold.

(continued)

Pair	Study	Classification	Our reasoning
– The coronavirus SARS-Cov-2 truly exists but did not emerge naturally; it was made in a laboratory as a bioweapon<br>– The coronavirus SARS-Cov-2 does not really exist; it is a hoax [was made up] by powerful governments to suppress the people	VP23s1<br>VP23s3<br>VP23s4	**Contrary**	If (a) Covid was made in a laboratory, then it exists. That is incompatible with (b) Covid not existing. But both could be false if Covid exists and originated in non-human animals.
– The people in the media who are claiming they are getting COVID-19 jabs are actually all paid actors – no one was even vaccinated against the virus<br>– The members of the elite are all getting vaccinated far from the public's eye	PT22s1<br>PT22s2	**Contrary**	It cannot both be true that (a) no one is vaccinated and that (b) members of the elite are getting vaccinated. But both could be false if many people - whether elite or not - are getting vaccinated.
– The police took a bribe to cover up the fact that Madeleine McCann was abducted by a sex trafficking gang<br>– The police took a bribe to cover up the fact that Madeleine McCann was killed by her parents	VP23s1	**Contrary**	It cannot both be true that (a) McCann was abducted by a gang and (b) McCann was killed by her parents at the same time. But both could be false if something else happened to McCann at that time.
– The virus is intentionally presented as dangerous<br>– Dark forces want to use the virus to rule the world	IH20s1<br>IH20s2<br>IH20s3	**Compatible**	It can (in principle) both be true that the virus (a) was presented as dangerous and (b) was of interest to authoritarian dark forces. The two statements do not constrain one another's truth values.
– The virus is intentionally presented as dangerous<br>– The virus is made to reduce the population	IH20s1<br>IH20s2<br>IH20s3	**Compatible**	It can (in principle) both be true that the virus (a) was presented as dangerous and (b) was made to reduce the population. The two statements do not constrain one another's truth values.
– The virus is intentionally presented as dangerous<br>– The virus was not created in a laboratory (r)	IH20s1<br>IH20s2<br>IH20s3	**Compatible**	It can (in principle) both be true that the virus (a) was presented as dangerous and (b) was not created in a lab. The two statements do not constrain one another's truth values.
– The virus is not worse than a flu<br>– Dark forces want to use the virus to rule the world	IH20s1<br>IH20s2<br>IH20s3	**Compatible**	It can (in principle) both be true that the virus (a) is not worse than the flu and (b) is of interest to authoritarian dark forces. The two statements do not constrain one another's truth values.
– The virus is not worse than a flu<br>– The virus is made to reduce the population	IH20s1<br>IH20s2<br>IH20s3	**Compatible**	It can (in principle) both be true that the virus (a) is not worse than the flu and (b) was made to reduce the population. The two statements do not constrain one another's truth values. But if both statements are true, the virus' death rate would be at most as high as that of the flu. Depending on the extent of the intended reduction, this could be viewed as inefficient, which could in turn suggest that the co-occurrence of the two statements is unlikely.
– The virus is not worse than a flu<br>– The virus was not created in a laboratory (r)	IH20s1<br>IH20s2<br>IH20s3	**Compatible**	It can (in principle) both be true that the virus (a) is not worse than the u and (b) was not created in a lab. The two statements do not constrain one another's truth values.
– There's now plenty of evidence that the coronavirus tests are rigged to show positive results<br>– The officials are intentionally hiding the fact that the numbers of infected are much higher than those reported	PT22s1<br>PT22s2	**Contrary**	It cannot both be true that the rate of covid infections is being both (a) exaggerated and (b) downplayed. But both could be false if infection rates are being estimated accurately.
– Al Qaeda fully prepared and carried out the attacks, but the U.S. government knew about them and deliberately did nothing to prevent them<br>– Al Qaeda had absolutely NOTHING to do with preparing and carrying out the attacks. The U.S. government completely prepared and carried out the attacks, and then blamed Al Qaeda	VP23s2	**Contrary**	It cannot both be true that (a) US government let Al Qaeda attack (passive complicity) and that (b) it directly carried out the attacks (active perpetration). But both could be false if neither scenario occurred.
– We should believe experts saying the virus is dangerous (r)<br>– Dark forces want to use the virus to rule the world	IH20s1<br>IH20s2<br>IH20s3	**Compatible**	It can (in principle) both be true that (a) it is good to believe the experts who pointed out that the virus is dangerous and that (b) the virus is of interest to authoritarian dark forces. The two statements do not constrain one another's truth values.
– We should believe experts saying the virus is dangerous (r)<br>– The virus is made to reduce the population	IH20s1<br>IH20s2<br>IH20s3	**Compatible**	It can (in principle) both be true that (a) it is good to believe the experts who pointed out that the virus is dangerous and that (b) the virus was made to reduce the population. The statements refer to different aspects of the topic (dangerousness, origin story) and do not constrain one another's truth values.

(continued)

Pair	Study	Classification	Our reasoning
– We should believe experts saying the virus is dangerous (r)<br>– The virus was not created in a laboratory	IH20s1<br>IH20s2<br>IH20s3	**Compatible**	It can (in principle) both be true that (a) it is good to believe the experts who pointed out that the virus is dangerous and that (b) the virus was not created in a lab. The two statements do not constrain one another's truth values.
– When the raid took place, Osama bin Laden was already dead<br>– Osama bin Laden is still alive	VP23s3<br>VP23s4	**Contrary**	It cannot both be true that (a) Bin Laden was already dead at the time of a raid and that (b) Bin Laden is still alive. But both could be false if Bin Laden died at some other time point.
– eljko Ranatovi Arkan faked his death<br>– eljko Ranatovi Arkan was killed by the secret security service	LK19s1<br>PT21s1<br>PT21s2	**Contrary**	That (a) Arkan faked his death at time x implies that he was still alive afterwards. This is incompatible with (b) Arkan having been killed by the secrete security service at time x. But both could be false if Arkan's life took some other course at time x.

Note. Publication and study examined (marked by s1 to s4) with sample description: LK19: [3], IH20: [4], PT21: [5], PT22: [6], and VP23: [2]. Items' different wording are reported in brackets. Items in IH20 including (r) are reversed. Items from VP23s2 were originally written in Dutch (and translated in English using Deepl); Items from LK19s1, PT21s1, PT21s2, PT22s1, PT22s2 were originally written in Serbian.

SM4. Implementation of the Incoherence score

The judgments participants gave to each pair of contrary statements can be thought of as a point in two-dimensional space. The axes of this space represent the two statements, each of which can in principle be judged to have a probability between 0 and 1. Because the statements are contrary, it would be incoherent to judge that both have probability 1. Further, to be coherent the joint probability of the two judgments must not exceed 1: $P(A) + P(B) \leq 1$. For each point representing incoherent judgments to the pair of statements, we can calculate the Cartesian distance to the nearest point that would have been coherent. For contrary statements, that nearest point lies on the diagonal line formed by the points (0,1) and (1,0), as shown in the middle panel of Figure Figure S2.

The Euclidean distance from a point (x_0, y_0) to a line with general equation $ax + by + c = 0$ is shown to the right side of Equation S1. Because for two contrary statements the Equation for the line is simply $x + y - 1 = 0$, the Equation to calculate the distance can be simplified as shown in the left side of Equation S1.

$$d = \frac{|ax_0 + by_0 + c|}{\sqrt{a^2 + b^2}} = \frac{|x_0 + y_0 - 1|}{\sqrt{2}} \tag{S1}$$

If instead of (x_0, y_0) we write (A,B) to represent participants' judgments for a pair of contrary statements A and B, and if we distinguish incoherent judgments for which we calculate the distance, from coherent judgments for which the distance is simply zero, we obtain Equation S2 as incoherence score I:

$$I(A, B) = \begin{cases} \frac{|A+B-1|}{\sqrt{2}}, & if\ A + B > 1 \\ 0, & if\ A + B \leq 1 \end{cases} \tag{S2}$$

An operationalization of Equation S2 as a function in R is shown in Code 1 below.

Code 1. R

```r
f <- function(A, B) {
  if (is.na(A) || is.na(B)) { return(NA) }
  s <- (A + B)
  r <- ifelse(s > 1, abs(s - 1) / sqrt(2), 0)
  return(r)
}
A  <- B <- seq(0, 1, 0.01)
AB <- expand.grid(A = A, B = B)
d  <- data.frame(AB, I = mapply(f, A, B))
```

Note that the coherence boundary defined by Equation S2 applies specifically to *contrary* pairs of statements. Calculation of incoherence is different for contradictory pairs because the coherent and incoherent regions are different, as illustrated in the left panel of Figure S2. For contradictory statements the only coherent response is on the $A + B = 1$ line, because B is the complement of A, and A + not-A = 1 (see Section SM2). For *compatible* pairs, shown in right panel of Figure S2, there is no coherence boundary in the sense that the entire probability space is coherent.

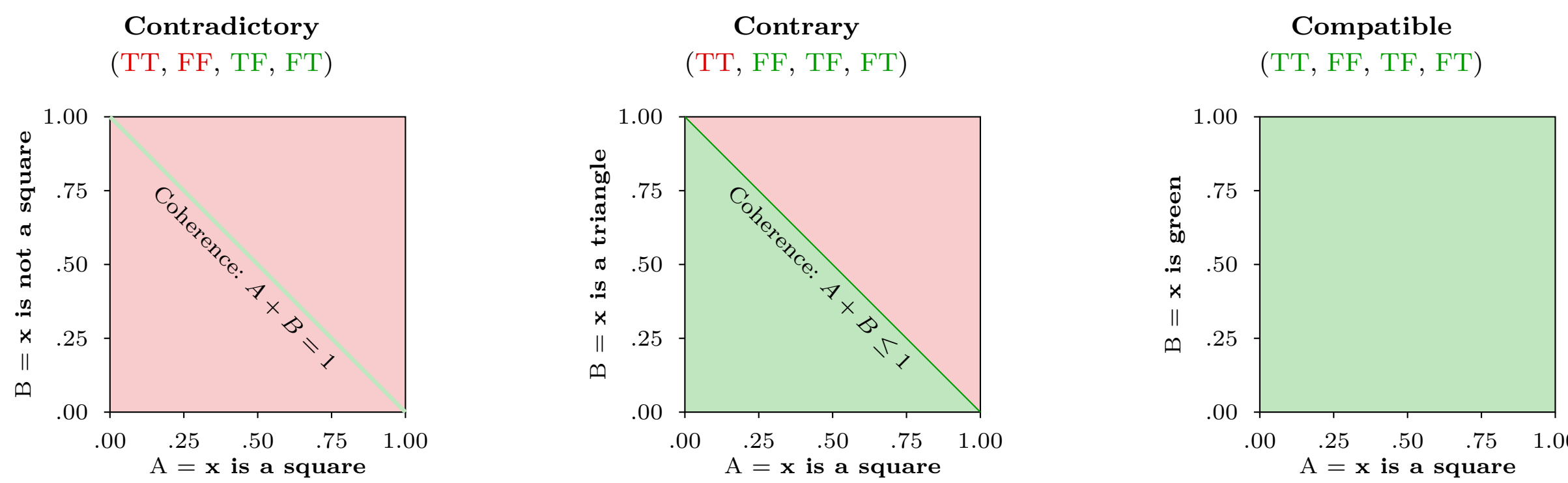

Figure S2. Logical relations between two statements Illustrations of three logical relations between statements: contradictory, contrary, and compatible. In each panel, the green regions indicate combinations of values that are logically coherent, while the red regions indicate combinations that are incoherent. The labels (TT, FF, TF, FT) represent the possible true/false combinations of the two statements (A and B).

SM5. Evaluation of the incoherence score based on the Euclidean distance

In the following, we provide a series of tests aimed at showing the goodness of our I score based on the Euclidean distance (see Section SM4). Specifically, we test whether the I score is superior to other incoherence measures, namely the item by item correlation (e.g., [1]), and the binary score based on the dichotomization of the agreements (from here on *binary calculation*) [5, 6]. We show that the I score is more sensitive in detecting small departures from coherence (Section SM5.1), has increased predictive validity (Section SM5.2), with higher internal reliability (Section SM5.3). The I score can be applied to any type of world-view related incoherence (beyond conspiratorial incoherence) to support theoretically grounded hypotheses where the correlation coefficient fails (Section SM5.4).

SM5.1. Sensitivity

Here, we illustrate the sensitivity of the I score (based on the probabilistic model of belief) in detecting fine-grained distinctions in incoherence, compared to the binary calculation (based on the dichotomous model of dis/belief) proposed by Petrović and Žeželj [5, 6] and later used by van Prooijen et al. [2]. Note that the binary calculation correlates highly with the I score in our sample ($r_{8869} = .87$, [.87, .88], $p < .001$), indicating that both indices—unlike correlations between responses—capture the same underlying construct of incoherence.

In Figure S3, the dashed red quadrant in the top-right represents the region of incoherent responses according to the binary measure, where simultaneous endorsement of both statements yields an incoherence score of 1. Outside this region, the binary calculation assigns an incoherence score of 0. The I score aligns with the binary calculation when evaluating pairs that lie within the coherence region (defined by $P_A + P_B \leq 1$) or when evaluating pairs at the point of maximum distance from the coherence region (Pairs 1, 2, and 3 in the figure). Note that the Euclidean distance underlying I ranges from 0 to .71, unlike the dichotomous 0-1 scale. However, the I score captures more nuanced variations outside the coherence region. Pair 4, for example, lies only slightly beyond the boundary and therefore receives a small I value of .04, a degree of incoherence overlooked by the binary measure. Likewise, although the binary measure assigns the same score (0) to both Pair 4 and Pair 5, the I score differentiates them meaningfully (Pair 4 = .04; Pair 5 = .28). Conversely, Pairs 5 and 6 receive very similar binary scores (0 and 1, respectively), yet their I scores (.28 and .35) indicate only a modest difference in degree of incoherence. The binary measure also fails to distinguish Pair 6 ($I = .35$) from Pair 3 ($I = .71$), as both receive a score of 1.

The sensitivity of I is especially valuable in aggregated measures of incoherence. Consider a respondent who is coherent on seven pairs ($A = 0$, $B = 0$, $I = 0$) but slightly incoherent on one pair ($A = .51$, $B = .51$, $I = .014$). Their aggregated incoherence is minimal ($\frac{7\times0+1\times.014}{8} = .00175$) and represents a very small value relative to the theoretical maximum ($.00175/.71 = .0025$), appropriately reflecting slight inconsistency. By comparison, the binary measure assigns a larger aggregated score ($\frac{7\times0+1\times1}{8} = .125$), a value that is much larger relative to its respective maximum ($.125/1 = .125$) when dichotomous scores are averaged. Thus, even a minimal departure from coherence is weighted appropriately by the I score. Such sensitivity is particularly advantageous when aggregated incoherence scores are regressed on other variables, which increases predictive validity (see Section SM5.2).

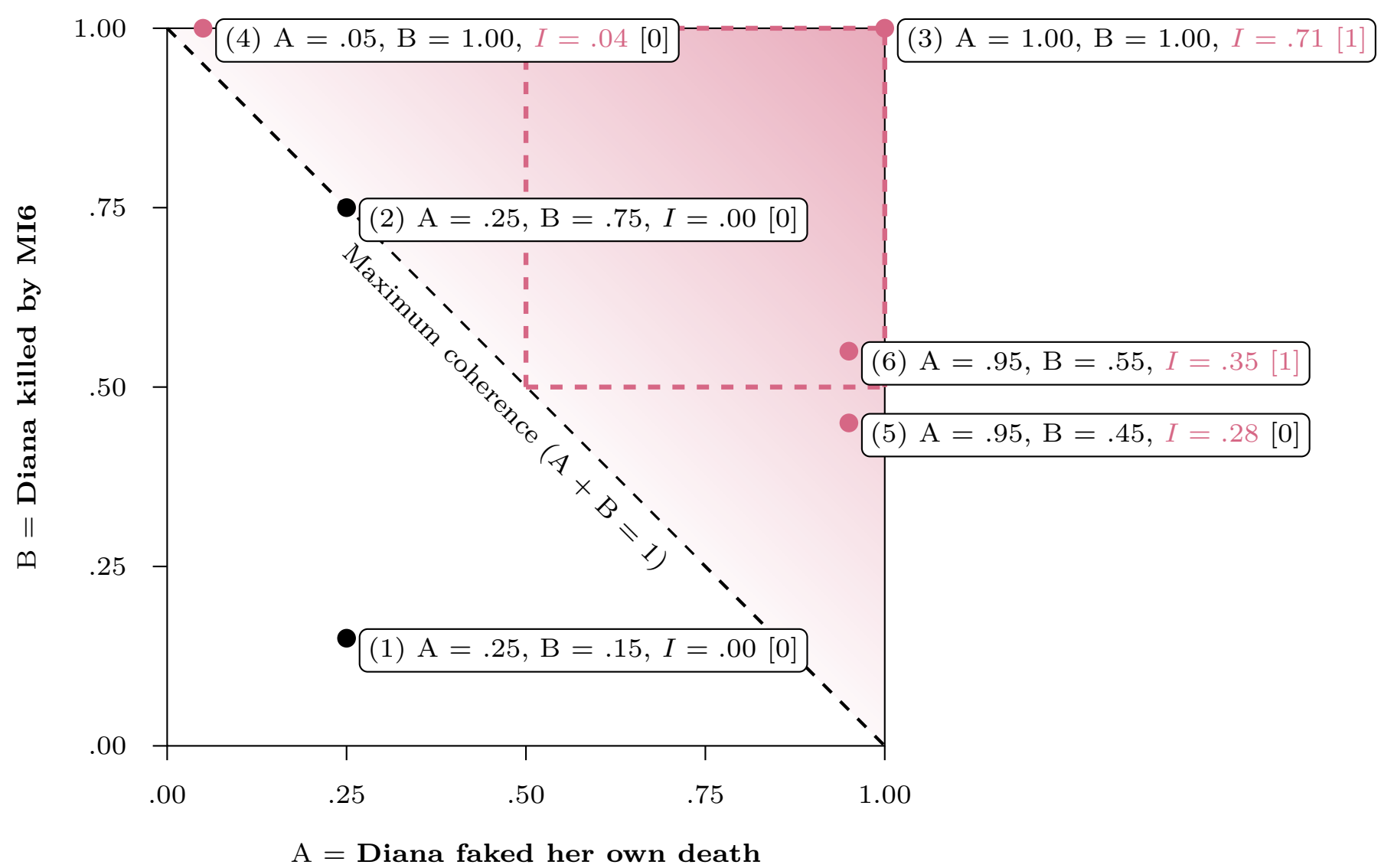

Figure S3. Sensitivity of the I score vs binary calculation. The black dashed diagonal line represents the coherence boundary calculated as the sum of subjective probabilities of A and B equal to 1 from which the I is calculated via Euclidean distance (see Section SM4). The top-right red dashed quadrant represents the binary calculation of incoherence [2, 5, 6]. The points illustrate different participants' responses with their values of I [and the binary calculation in square brackets]; see text for details.

SM5.2. Predictive validity

To assess predictive validity, we compared the two scoring methods, namely the continuous I score and the binary measure, in terms of the effect sizes they produce when correlated with a related construct. Specifically, we examined two indicators of incoherence: conspiratorial

incoherence and the *doublethink* (DT) scale introduced by Petrović and Žeželj [5]. Both scales capture incoherence stemming from mutually incompatible statements. DT differs from conspiratorial incoherence in that it comprises a set of incompatible, non-conspiratorial statements (e.g., *Some people are just born evil* vs *All children are innocent by nature*), intended to measure a general tendency toward incoherence rather than content-specific conspiratorial incoherence. Given that both constructs tap into the same underlying disposition (i.e., incoherence), they are expected to correlate positively. Because the item-level responses are identical across scoring methods and only the scoring procedure differs, a larger effect size for one method indicates superior predictive validity. To this end, we selected the studies that measured both DT and conspiratorial incoherence [5, 6]. For each scale in each study, we computed incoherence using both the continuous I score and the binary method, and we computed the correlation between DT and conspiratorial incoherence. Across all studies, the I score yielded larger effect sizes than the binary calculation (originally reported effect sizes are shown in parentheses): **PT21s1**: $r_{238} = .32$, $[.20, .43]$, $p < .001$ ($r = .25$, $p < .01$); **PT21s2**: $r_{165} = .25$, $[.10, .39]$, $p = .001$ ($r = .04$, $p = ns$); **PT22s1**: $r_{288} = .58$, $[.50, .65]$, $p < .001$ ($r = .44$, $p < .001$); **PT22s2**: $r_{279} = .57$, $[.48, .64]$, $p < .001$ ($r = .36$, $p < .001$).

SM5.3. Internal consistency

Because the continuous I score increases variability in the degree of incoherence compared to the collapsed responses into dichotomies, it should naturally improve internal consistency. We therefore examined whether the continuous scoring method produces a scale with higher internal consistency, as assessed by Cronbach's α, compared to the binary scoring method. Higher internal consistency indicates that the pairs function more uniformly as indicators of the same latent construct, incoherence. For each study listed in Table 1, we computed conspiratorial incoherence for every pair using both the continuous I score and the binary method. We then calculated Cronbach's α on each resulting scale. Across all datasets, the continuous scoring method consistently yielded higher α values (with an average improvement of $M = 0.12$, range: 0.06–0.23): LK19s1: .60/.66 ($\Delta = .06$); PT21s1: .21/.30 ($\Delta = .09$); PT21s2: .35/.53 ($\Delta = .18$); PT22s1: .72/.80 ($\Delta = .08$); PT22s2: .61/.73 ($\Delta = .12$); VP23s1: .41/.56 ($\Delta = .15$); VP23s2: .38/.47 ($\Delta = .09$); VP23s3: .61/.72 ($\Delta = .11$); VP23s4: .45/.69 ($\Delta = .23$).

SM5.4. Applied to other worldviews

To examine whether our framework generalizes beyond the conspiratorial domain, we reanalyzed data from Morgenroth et al. [7], who investigated the coexistence of incompatible beliefs in the context of anti-transgender rhetoric. As in similar work [1, 8], the authors hypothesized a positive relationship between the endorsement of incompatible anti-transgender arguments that both sustain a higher-order ideological worldview, in this case opposition to trans-inclusive policies, characterized by the rejection of diverse gender identities (i.e., gender-affirming beliefs) and the view that gender is innate and informative (i.e., gender essentialism). As a case study, we focused on one pair of incompatible statements: *Trans people are a small minority* and *Trans people are everywhere*. Each construct was measured with three items, which we averaged and rescaled from the 7-point Likert to the 0–1 range. We then computed our incoherence index, I, for each participant.

In their Study 1, the I score derived from endorsing both anti-transgender arguments was positively correlated with gender essentialism ($r_{265} = .43$, $[.33, .53]$, $p < .001$) and negatively correlated with gender-affirming beliefs ($r_{265} = -.53$, $[-.61, -.44]$, $p < .001$). In Study 2, the authors additionally categorized participants as either supporters or opponents of trans-inclusive policies (similar to the binary operationalization of rejection of the official story in van Prooijen et al. [2]). Across the entire sample, the correlation between the two incompatible statements was positive ($r_{690} = .66$, $[.62, .70]$, $p < .001$), and this relationship did not differ substantially when the sample was subset in opponents ($r_{345} = .52$, $[.44, .59]$, $p < .001$) and supporters ($r_{343} = .55$, $[.47, .62]$, $p < .001$). However, when applying our I score, we found that opponents exhibited significantly higher incoherence ($M = 0.150$, $SD = 0.191$, $N = 347$) than supporters ($M = 0.015$, $SD = 0.071$, $N = 345$; $t_{440.65} = 12.39$, $p < .001$, $d = .94$). In other words, we provided evidence for what we believe was the main research question of Morgenroth et al. [7], namely higher worldview-related incoherence in opponents (something that was not answered by the correlation coefficients alone).

To complement and enrich our assessment of predictive validity (see Section SM5.2), we also compared effect sizes obtained using both the binary and the I measure (which again were highly correlated: $r_{690} = .84$, $[.82, .86]$, $p < .001$). The difference between opponents and supporters remained large and significant under both scoring methods, but the effect size was smaller when using the binary measure ($t_{452.82} = 10.24$, $p < .001$, $d = .78$).

Taken together, these findings demonstrate that our framework extends beyond conspiratorial domains and applies to ideological worldviews more broadly. Crucially, this reanalysis shows empirically that simple correlations between agreement with incompatible statements are not, by themselves, diagnostic of incoherence.

SM6. Replicating the analyses without VP23

In VP23's studies [2], rejection of the official narrative correlates with conspiracy mentality ($r_{794} = .58$, [.53, .63], $p < .001$, in Study 1 where both measures were available) and is systematically associated with higher incoherence (see Table S5). However, our operationalization of conspiracism based on the average of rejection of the official story could be criticized for not representing an explicit measure of conspiratorial thinking. To address this concern, we run our analyses after excluding the VP23 studies that did not include an explicit measure of conspiracism. Specifically, we removed Studies 2, 3, and 4, retaining only Study 1, which included the Conspiracy Mentality Scale [9]. The resulting sample size for this analysis is $N = 1,745$.

We replicated the analyses reported in the main text and estimated a zero-inflated (ZI) model. In the conditional component, conspiracism remained positively associated with incoherence ($b = 1.24$, $SE = .25$, $z = 4.97$, $p < .001$, $OR = 3.45$); the corresponding odds ratios translated to $r = .32$ and $d = .68$. In the zero-inflation component, conspiracism substantially reduced the likelihood of excess zeros ($b = 5.47$, $SE = .52$, $z = 10.55$, $p < .001$, $OR = 236.448$); the odds of observing incoherence increased sharply, with an odds ratio of .004 for a one-unit increase in conspiracism ($r = .83$, $d = 3.01$). The predicted probability of incoherence rose from 1% at the lowest levels of conspiracism to 91% at the highest. Taken together, these analyses show that the results remain largely unchanged when VP23's Studies 2 to 4 are excluded.

SM7. Statistical modelling

We compared candidate models using beta and gamma distributions, both suitable for modeling continuous data with a lower bound at zero. Other options—such as the t, binomial, or Poisson distributions—were not considered, as they are inappropriate for continuous, bounded data like our I scores. For comparability with simpler and more commonly used methods, we also report a model fitted with a Gaussian distribution. We fitted three mixed-effects models predicting `incoherence` from `conspiracy`, including `study` as a random grouping factor with both a random intercept and a random slope for the predictor. The models were specified in R as follows: **Gaussian:** `lmer(scale(incoherence) ~ scale(conspiracy) + (1 + scale(conspiracy) | study), data)`; **ZI-Beta:** `glmmTMB(incoherence ~ conspiracy + (1 + conspiracy | study), ziformula = ~ conspiracy + (1 + conspiracy | study), family = beta_family(), data)`; **ZI-Gamma:** `glmmTMB(incoherence ~ conspiracy + (1 + conspiracy | study), ziformula = ~ conspiracy + (1 + conspiracy | study), family = ziGamma(link = 'log'), data)`. The gamma model outperformed the beta model, yielding lower AIC, BIC, and a slightly better overdispersion rate (see Table S2).

Table S2. Model comparison

	Gaussian	ZI-Beta	ZI-Gamma
(Intercept)	-0.30		
	$[-0.60, 0.00]$		
conspiracism	0.65^{*}		
	$[0.49, 0.81]$		
Conditional part: (Intercept)		-2.91^{*}	-3.07^{*}
		$[-3.05, -2.77]$	$[-3.27, -2.87]$
Conditional part: conspiracism		1.22^{*}	1.28^{*}
		$[1.08, 1.36]$	$[1.09, 1.47]$
Zero-inflation component: (Intercept)		3.52^{*}	3.51^{*}
		$[2.70, 4.33]$	$[2.72, 4.31]$
Zero-inflation component: conspiracism		-5.88^{*}	-5.88^{*}
		$[-6.90, -4.85]$	$[-6.89, -4.87]$
Marginal R^2	0.31	0.01	0.23
Conditional R^2	0.48	0.01	0.32
Overdispersion	0.9, $p = .256$	1.12, $p = .416$	1.02, $p = .824$
AIC	21464	376	97
BIC	21506	440	175
AIC	21463.96	376.46	96.93
Num. obs.	8590	8590	8590
Num. groups: study	8	8	8
Var: study (Intercept)	0.18		
Var: study conspiracism	0.05		
Cov: study (Intercept) conspiracism	-0.05		
Var: Residual	0.71		
Var (count model): study (Intercept)		0.02	0.06
Var (count model): study conspiracism		0.00	0.02
Var (zero model): study (Intercept)		1.08	1.03
Var (zero model): study conspiracism		1.58	1.54

* Null hypothesis value outside the confidence interval.

SM8. Results including compatible items

In Table S3, we report the results of rerunning the analyses while including item pairs from previous research that are not logically incompatible (e.g., *Josip Tito was an American spy* and *Josip Tito was a Russian spy*; see Table S1). We also included the studies from Imhoff and Lamberty [4], obtaining a total sample size of 9,396. The results do not change substantially, but the effect sizes are somewhat larger. Because these additional items are logically compatible (having a larger coherence space, see Figure S2), this pattern indicates an artificial inflation of the incoherence estimate.

Table S3. Model Comparison including compatible items

	Gaussian	ZI-Gamma
(Intercept)	-0.34^*	
	$[-0.57, -0.11]$	
conspiracism	0.68^*	
	$[0.50, 0.85]$	
Conditional part: (Intercept)		-3.32^*
		$[-3.56, -3.08]$
Conditional part: conspiracism		1.62^*
		$[1.26, 1.99]$
Zero-inflation component: (Intercept)		3.62^*
		$[2.99, 4.25]$
Zero-inflation component: conspiracism		-5.71^*
		$[-6.53, -4.89]$
Marginal R^2	0.33	0.31
Conditional R^2	0.49	0.41
Overdispersion	0.94, $p = .632$	1.01, $p = .824$
AIC	23489	330
BIC	23532	409
AIC	23489.33	329.96
Num. obs.	9396	9396
Num. groups: study	11	11
Var: study (Intercept)	0.14	
Var: study conspiracism	0.08	
Cov: study (Intercept) conspiracism	-0.05	
Var: Residual	0.71	
Var (count model): study (Intercept)		0.10
Var (count model): study conspiracism		0.25
Var (zero model): study (Intercept)		0.84
Var (zero model): study conspiracism		1.22

* Null hypothesis value outside the confidence interval.

SM9. Counting frequency of incoherent participants

In order to connect our results to existing approaches for analyzing conspiratorial incoherence data [2, 3, 5, 6], we counted how many participants with a high conspiracism endorsed at least one pair of incompatible CTs. Note that here, because incoherence is theorized to be an attribute of conspiracism, the relevant estimates of incoherence are for people high in conspiracism—contrary to the previous estimates of the prevalence of incoherence across the whole population surveyed [see e.g., 2, 3, 5, 6]; it is inappropriate to include the entire sample because non-believers, theoretically, are not expected to exhibit incoherence.

We dichotomized endorsement of incompatible pairs and conspiracism to create a contingency table counting participants high and low in conspiracism × high and low in incoherence. For conspiracism, high and low groups were created using a cutoff of .50 on the rescaled conspiracism measure, excluding the midpoint (high $> .50$, $N = 1{,}347$; low $< .50$, $N = 6{,}742$). Note that imposing a threshold on a continuous variable becomes unavoidably arbitrary; a limitation that is not specifically tied to our variables. We have chosen the aggregated midpoint (corresponding to *unsure*) so to split a general dis/agreement (or endorsement) of conspiracism items. Incoherence was calculated using both the binary approach and the one based on probabilistic coherence. In the binary approach, high incoherence was operationalized as endorsing at least one pair of incompatible CTs via the binary operationalization of dis/belief (for similar operationalizations, see 3, 5, 6 for incoherence and 10–12 for conspiracy beliefs), while low incoherence was defined as not endorsing any incompatible pairs. In the probabilistic approach, high incoherence was calculated as exceeding the probabilistic constraint in at least one pair, whereas low incoherence was defined as not exceeding the constraint in all incompatible pairs.

In Table S4, we report the contingency table of conspiracism × coherence using both the binary and the probabilistic calculation. As for the binary calculation, we found that 39.64% of participants high in conspiracism endorsed at least one pair of incompatible CTs, compared to 3.62% of participants low in conspiracism ($OR = 17.48$, $[14.74, 20.77]$, $p < .001$, $d = 1.58$, $r = .62$). As for incoherence computed using the probabilistic framework, we found that 58.43% of participants high in conspiracism resulted incoherent in at least one pair of incompatible CTs, compared to 11.45% of participants low in conspiracism ($OR = 10.86$, $[9.5, 12.42]$, $p < .001$, $d = 1.32$, $r = .55$).

We report an additional subset analysis of VP23's studies [2], which do not include an explicit measure of conspiracism. Instead, the authors asked participants, for each event, whether or not they believed the official story. In Table S5, we report, separately for participants who accepted and rejected the official story, the frequency and percentage of responses in which two incompatible CTs were endorsed exceeding the probabilistic constraints, along with the corresponding mean I scores and the mean of the difference.

Table S4. Contingency table of conspiracism × coherence calculated via the the binary and probabilistic method

	**Binary agreement**		**Probabilistic incoherence**		
	incoherent	coherent	incoherent	coherent	Total N
High conspiracism	534 (39.64%)	813 (60.36%)	787 (58.43%)	560 (41.57%)	1,347 (16.65%)
Low conspiracism	244 (3.62%)	6,498 (96.38%)	772 (11.45%)	5,970 (88.55%)	6,742 (83.35%)
Total N	778 (9.62%)	7,311 (90.38%)	1,559 (19.27%)	6,530 (80.73%)	8,089 (100.00%)

Table S5. VP23's differences in incoherence split by acceptance/rejection of the official story

	N_{total}	# incoherent responses		Mean of I scores		
Study, *Topic*	acc/rej	$N_{acc/rej}$	$\%_{acc/rej}$	$M_{acc/rej}$	Δ [95% CIs]	d
s1, *Covid*	573 / 223	7 / 53	1.22% / 23.77%	.005 / .073	.07 [.05, .09]	.76
s1, *Climate*	735 / 61	18 / 32	2.45% / 52.46%	.006 / .156	.15 [.10, .20]	2.24
s1, *911*	637 / 159	14 / 53	2.2% / 33.33%	.008 / .105	.10 [.07, .12]	1.05
s1, *Maddie*	615 / 181	20 / 56	3.25% / 30.94%	.008 / .095	.09 [.06, .11]	.96
s1, *Cancer*	633 / 163	33 / 63	5.21% / 38.65%	.015 / .140	.13 [.09, .16]	1.08
s1, *Hitler*	657 / 139	21 / 37	3.2% / 26.62%	.006 / .062	.06 [.04, .08]	.94
s1, *Diana*	572 / 224	4 / 13	0.7% / 5.8%	.002 / .013	.01 [.00, .02]	.26
s1, *Epstein*	383 / 413	8 / 88	2.09% / 21.31%	.007 / .057	.05 [.04, .06]	.50
s2, *Covid*	3970 / 345	33 / 122	0.83% / 35.36%	.002 / .117	.12 [.10, .13]	1.91
s2, *Climate*	4082 / 394	59 / 262	1.45% / 66.5%	.004 / .252	.25 [.22, .27]	3.16
s2, *911*	3636 / 387	9 / 84	0.25% / 21.71%	.001 / .058	.06 [.04, .07]	1.35
s2, *Hitler*	3678 / 168	12 / 26	0.33% / 15.48%	.001 / .036	.03 [.02, .05]	1.35
s3, *Covid*	534 / 262	12 / 74	2.25% / 28.24%	.009 / .090	.08 [.06, .10]	.73
s3, *Climate*	707 / 89	14 / 55	1.98% / 61.8%	.006 / .203	.20 [.15, .24]	2.35
s3, *911*	638 / 158	10 / 49	1.57% / 31.01%	.006 / .101	.09 [.07, .12]	1.05
s3, *Cancer*	586 / 210	15 / 17	2.56% / 8.1%	.007 / .025	.02 [.00, .03]	.29
s3, *Hitler*	693 / 103	81 / 28	11.69% / 27.18%	.036 / .088	.05 [.02, .09]	.43
s3, *Epstein*	287 / 509	8 / 133	2.79% / 26.13%	.006 / .075	.07 [.06, .08]	.59
s3, *Osama*	750 / 39	3 / 8	0.4% / 20.51%	.001 / .073	.07 [.02, .13]	1.84
s3, *Elvis*	742 / 29	1 / 7	0.13% / 24.14%	.000 / .073	.07 [.02, .13]	2.56
s4, *Covid*	477 / 311	15 / 60	3.14% / 19.29%	.009 / .055	.05 [.03, .06]	.50
s4, *Climate*	662 / 126	12 / 54	1.81% / 42.86%	.007 / .119	.11 [.08, .14]	1.34
s4, *911*	552 / 236	11 / 62	1.99% / 26.27%	.005 / .075	.07 [.05, .09]	.80
s4, *Cancer*	518 / 270	15 / 75	2.9% / 27.78%	.008 / .084	.08 [.06, .10]	.76
s4, *Hitler*	638 / 150	10 / 24	1.57% / 16%	.003 / .034	.03 [.02, .05]	.72
s4, *Epstein*	285 / 503	20 / 116	7.02% / 23.06%	.017 / .062	.04 [.03, .06]	.39
s4, *Osama*	710 / 78	7 / 11	0.99% / 14.1%	.003 / .041	.04 [.01, .06]	.76
s4, *Diana*	582 / 206	9 / 24	1.55% / 11.65%	.003 / .027	.02 [.01, .04]	.50

Note. # of incoherent responses is expressed as count ($N_{acc/rej}$) and percentage ($\%_{acc/rej}$) relative to the group of participants who either accepted or rejected the official story. The I scores are reported as means, as well as the mean difference in incoherence (rejection − acceptance; Δ) [with 95% confidence intervals] and Cohen's d.

SM10. Random responses

Do incoherent responses reflect random responding? If incoherent responses arise from careless or chance responding, then randomly generated responses preserving the observed marginal distributions for each item should exhibit the same level of incoherence as the actual data. To test this, we simulated a null model in which responses to each item within a pair were generated independently.

For each item in each incompatible pair ($N = 46$) across 8 studies, we drew independent responses from a truncated normal distribution parameterized by the observed mean and standard deviation, bounded within the 0–1 probability range. Simulated values were then mapped to the nearest observed response category (e.g., 0, .25, .50, .75, and 1.00 on a 5-point Likert scale). This yielded, for each pair, two datasets: one observed and one simulated. We computed average incoherence (Euclidean distance from the coherence boundary) for each dataset and tested for differences using a multilevel regression predicting average incoherence from dataset type (observed vs simulated), with pair as a grouping factor.

Incoherence was significantly higher in the observed dataset ($M_{OBS} = 0.04$, $SD = 0.03$, range: 0.00–0.14) than in the simulated dataset ($M_{SIM} = 0.03$, $SD = 0.02$, range: 0.00–0.09; $\beta = .39$, $SE = .09$, $t = 4.17$, $p < .001$). This pattern held when restricting the analysis to participants high in conspiracism (i.e., $> .50$ on the rescaled measure; observed $M_{OBS} = 0.08$, $SD = 0.05$, range: 0.01–0.22 vs simulated $M_{SIM} = 0.07$, $SD = 0.04$, range: 0.01–0.16; $\beta = .21$, $SE = .09$, $t = 2.42$, $p = .02$). Because observed incoherence exceeded that generated under the null model of independent responding, the findings indicate systematic above-chance co-endorsement of incompatible CTs rather than random or careless responding.

SM11. List of incompatible pairs used in Study 2

A screenshot example of how participants viewed the vignette and the incompatible pair is shown in Figure S4. The 16 vignettes used in Study 2 along with the four incompatible pairs are listed in Table S6.

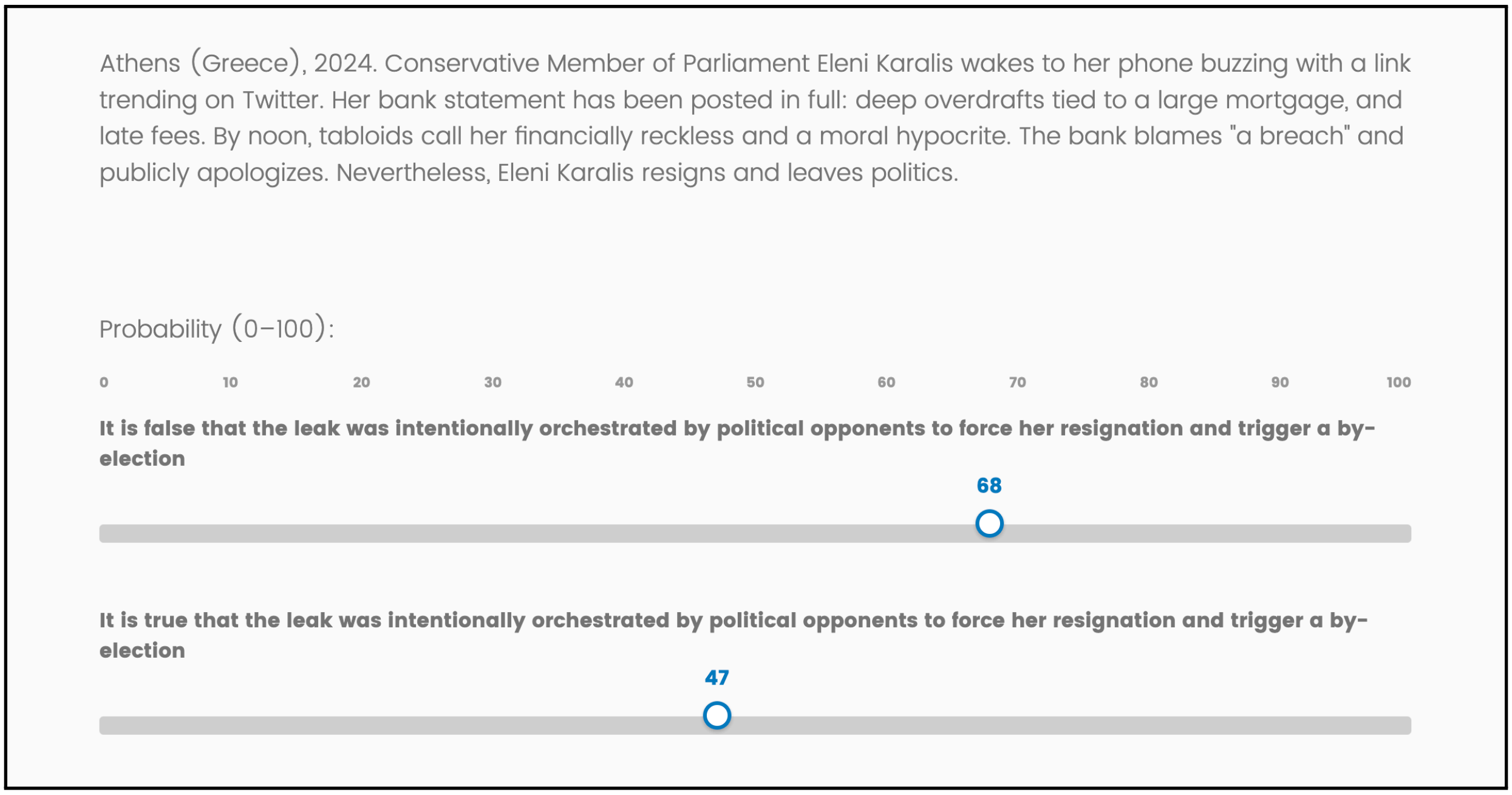

Figure S4. Screenshot of vignette presentation in Study 2. Participants read a fictitious vignette followed by two incompatible explanations (here, a conspiracy contradictory pair). For each explanation, participants rated the likelihood of incompatible explanations being true on a 0–100 probability scale. The chosen value was always visible to facilitate calibration.

Table S6: Vignettes and incompatible pairs used in Study 2.

**[1]**	*Chloe is an 8-year-old girl and a promising young skier. Her father, an influential member of the European Parliament, often takes her skiing in the Swiss Alps. One day, Chloe separated from her group during ski school and did not return home. Her father asked the European Union for funds to support a search for her, triggering negative reactions from colleagues and opponents. After three days, Chloe returned home.*
**CTCD**	It is true that Chloe has been secretly held by her father in order to misuse public funds
	It is false that Chloe has been secretly held by her father in order to misuse public funds
**CTCR**	It is true that Chloe has been secretly held by her father in order to misuse public funds
	It is true that Chloe escaped an environment in which she was secretly forced into child labor
**NCCD**	It is true that Chloe accidentally fell from a nearby cliff
	It is false that Chloe accidentally fell from a nearby cliff
**NCCR**	It is true that Chloe accidentally fell from a nearby cliff
	It is true that Chloe was caught in a sudden avalanche
**[2]**	*A few days before his fourth birthday in July 2021, Lucas was kept home from kindergarten because he had a high fever. On the day of his birthday, the fever had not gone down, and he was rushed to the hospital. Later that day, doctors had to place Lucas in a medically induced coma to help him recover from severe brain inflammation (encephalitis).*
**CTCD**	It is true that the encephalitis was caused mainly by the COVID-19 vaccine administered a month earlier
	It is false that the encephalitis was caused mainly by the COVID-19 vaccine administered a month earlier
**CTCR**	It is true that the encephalitis was caused mainly by the COVID-19 vaccine administered a month earlier
	It is true that the encephalitis was caused mainly by the MMR vaccine administered a month earlier
**NCCD**	It is true that the encephalitis was caused mainly by accidental food poisoning after eating his mum's tiramisu
	It is false that the encephalitis was caused mainly by accidental food poisoning after eating his mum's tiramisu
**NCCR**	It is true that the encephalitis was caused mainly by accidental food poisoning after eating his mum's tiramisu
	It is true that the encephalitis was caused mainly by an infected cut Lucas got accidentally in his granddad's garage

Continued on next page

Table S6 – continued from previous page

[3] *John, a longtime heavy smoker, drinker, and recreational drug user, owns a multinational chain of fine-dining restaurants that caters to high society, including politicians and other influential people. He planned to launch a political career, claiming he knew many secrets. At the public presentation of his new party, an attendee shouted "They also know your secrets!". A few days later, he was found unconscious in the basement of one of his restaurants in London. He died in the hospital a few days after that.*

**CTCD**	It is true that John was murdered by political opponents
	It is false that John was murdered by political opponents
**CTCR**	It is true that John was murdered by political opponents
	It is true that John attempted suicide and died
**NCCD**	It is true that John died of an accidental heart attack caused mainly by physical exertion while carrying heavy food
	It is false that John died of an accidental heart attack caused mainly by physical exertion while carrying heavy food
**NCCR**	It is true that John died of an accidental heart attack caused mainly by an overdose of cocaine
	It is true that John died of an accidental heart attack caused mainly by physical exertion while carrying heavy food

[4] *Aksel, 27, was a guitarist in a popular heavy metal band influenced by Norwegian black and death metal. On the morning of March 28, 2008, he arrived at his university in Oslo carrying two 9mm pistols. He entered the aula magna and opened fire, but two professors subdued him within moments. No one was killed, though Aksel served two years in prison. Today, he deeply regrets what he did.*

**CTCD**	It is true that Aksel received a message on his phone with clear instructions to kill (so to distract the public from an unrelated political scandal involving the prime minister)
	It is false that Aksel received a message on his phone with clear instructions to kill (so to distract the public from an unrelated political scandal involving the prime minister)
**CTCR**	It is true that Aksel received a message on his phone with clear instructions to kill (so to distract the public from an unrelated political scandal involving the prime minister)
	It is true that Aksel was instructed to kill by hidden subliminal messages intentionally embedded in heavy metal music
**NCCD**	It is true that Aksel acted mainly because of a severe mental health crisis linked to long-term social isolation, bullying, and accumulated resentment
	It is false that Aksel acted mainly because of a severe mental health crisis linked to long-term social isolation, bullying, and accumulated resentment
**NCCR**	It is true that Aksel acted mainly because of a severe mental health crisis linked to long-term social isolation and accumulated resentment
	It is true that Aksel acted mainly to gain notoriety for his band and become famous by dying at age 27, like Jimi Hendrix or Kurt Cobain

[5] *As of today, pancreatic cancer remains difficult to cure, and survival rates remain low overall. In 2012, "Di Bella therapy", based on high-dose vitamins, dietary changes, and specific pancreatic cleanse protocols, began attracting attention in Italy. By mid-2013, more than one thousand patients had enrolled. However, after about a year of clinical evaluation, the Italian government abruptly halted the program.*

**CTCD**	It is true that the program was halted mainly because it was cheap and pharmaceutical companies could not profit from it
	It is false that the program was halted mainly because it was cheap and pharmaceutical companies could not profit from it
**CTCR**	It is true that the program was halted mainly because it was cheap and pharmaceutical companies could not profit from it
	It is true that the program was halted mainly because scientists and medical authorities wanted to protect their status and dismissed it as pseudoscience
**NCCD**	It is true that the program was halted mainly because the clinical trials failed to show therapeutic benefit
	It is false that the program was halted mainly because the clinical trials failed to show therapeutic benefit
**NCCR**	It is true that the program was halted mainly because the clinical trials failed to show therapeutic benefit
	It is true that the program was halted mainly because safety concerns emerged during the clinical trials

[6] *Elena, the Duchess of Valdoria, was known for her charity work and for clashing with the royal family. At the annual Independence Day celebration in 1994, she spoke publicly about the systematic persecution of Valdoria's largest minority community and alleged that members of the royal family helped conceal it. Later that night, her vehicle crashed on a coastal road, leaving Elena in a coma. A week later, her family decided to withdraw life support.*

**CTCD**	It is true that Elena was killed by Valdoria's royal family
	It is false that Elena was killed by Valdoria's royal family
**CTCR**	It is true that Elena was killed by Valdoria's royal family
	It is true that Elena staged her death with help from insiders to escape the royal family and later bring evidence to an international court
**NCCD**	It is true that the crash happened mainly due to an accidental mechanical failure in the car's braking system
	It is false that the crash happened mainly due to an accidental mechanical failure in the car's braking system
**NCCR**	It is true that the crash happened mainly due to an accidental mechanical failure in the car's braking system
	It is true that the crash happened mainly because the driver accidentally lost control in heavy rain after taking the coastal road too fast

[7] *Armand Leclair was a French singer-songwriter who became "the voice" of France's student demonstrations in May 1968. His last song, "Les Dossiers", accused the French government of secret blacklists, police brutality, and silencing dissidents. Days after a radio performance that drew massive attention, Armand said in his final interview that he felt he was being followed and that "someone wants my lyrics buried". Two weeks later, he was found dead in his apartment.*

**CTCD**	It is true that Armand was killed by French state authorities to silence his accusations
	It is false that Armand was killed by French state authorities to silence his accusations
**CTCR**	It is true that Armand was killed by French state authorities to silence his accusations
	It is true that Armand was killed by an independent anti-protest extremist
**NCCD**	It is true that Armand died by suicide after escalating pressure, threats, and exhaustion
	It is false that Armand died by suicide after escalating pressure, threats, and exhaustion

Continued on next page

Table S6 – continued from previous page

**NCCR**	It is true that Armand died by suicide after escalating pressure, threats, and exhaustion
	It is true that Armand died in an accidental overdose after mixing alcohol with prescription sedatives
**[8]** *HIV/AIDS has killed millions of people since the early 1980s. Early reports often stigmatized HIV as a "gay virus", even though it spreads through many communities worldwide. In the US, HIV has disproportionately affected Black/African American communities, and it has continued to devastate populations in parts of Africa. Today, there is still no definitive cure and no widely available vaccine, though one vaccine candidate is being tested in clinical trials.*	
**CTCD**	It is true that the vaccine is life saving and is deliberately being withheld to eliminate gay and Black communities
	It is false that the vaccine is life saving and is deliberately being withheld to eliminate gay and Black communities
**CTCR**	It is true that the vaccine is life saving and is deliberately being withheld to eliminate gay and Black communities
	It is true that the vaccine is dangerous and is deliberately distributed on the black market to harm gay and Black communities
**NCCD**	It is true that the main challenge to developing a widely available vaccine is lack of funding
	It is false that the main challenge to developing a widely available vaccine is lack of funding
**NCCR**	It is true that the main challenge to developing a widely available vaccine is lack of funding
	It is true that the main challenge to developing a widely available vaccine is technological limitations
**[9]** *Thomas is a young lawyer from Zurich (Switzerland). Since being diagnosed with ADHD in his teens, Thomas has been taking stimulant medication (Concerta, by Janssen Pharmaceuticals). Over the last two months, however, he has started taking extra doses on stressful days, feels unusually irritable when he skips a dose, and has begun "counting pills" anxiously. After a weekend without his medication, he experienced tremors, insomnia, and a racing heartbeat.*	
**CTCD**	It is true that Janssen Pharmaceuticals adds hidden additives to Concerta mainly to increase dependence and increase profit
	It is false that Janssen Pharmaceuticals adds hidden additives to Concerta mainly to increase dependence and increase profit
**CTCR**	It is true that Janssen Pharmaceuticals adds hidden additives to Concerta mainly to increase dependence and increase profit
	It is true that Janssen Pharmaceuticals adds hidden additives to Concerta mainly to increase side-effects and drive repeat medical visits
**NCCD**	It is true that Thomas' symptoms are mainly explained by medication side effects triggered by dose escalation without medical supervision
	It is false that Thomas' symptoms are mainly explained by medication side effects triggered by dose escalation without medical supervision
**NCCR**	It is true that Thomas' symptoms are mainly explained by medication side effects triggered by dose escalation without medical supervision
	It is true that Thomas' symptoms are mainly explained by an emerging anxiety disorder/panic episodes triggered by job pressure
**[10]** *In 2010, in the suburbs of Sofia (Bulgaria), several people noticed a silent oval craft hovering above a field at dusk. Viktoria Nikolova, a journalism student, filmed it on her early smartphone and uploaded the clip to Facebook, which was still new among her friends. According to Viktoria, the post vanished overnight, and none of her friends saw it. Yet Viktoria was sure she had uploaded it.*	
**CTCD**	It is true that Facebook removed the video because it has a secret agreement with the government to hide evidence of alien contact
	It is false that Facebook removed the video because it has a secret agreement with the government to hide evidence of alien contact
**CTCR**	It is true that Facebook removed the video because it has a secret agreement with the government to hide evidence of alien contact.
	It is true that the alien ship's magnetic field erased nearby mobile phone data, including Viktoria's video
**NCCD**	It is true that the video disappeared because of an accidental technical problem typical of early smartphones
	It is false that the video disappeared because of an accidental technical problem typical of early smartphones
**NCCR**	It is true that the video disappeared because of an accidental technical problem typical of early smartphones
	It is true that the video disappeared because Viktoria accidentally deleted it before uploading it
**[11]** *Rosa develops a sore throat, fatigue, and a cough. Her doctor explains it is likely an infection that usually resolves on its own and says homeopathic remedies have not shown effects beyond placebo. Rosa takes a homeopathic preparation anyway because her best friend strongly recommended it. Three days later, she feels significantly better and tells her doctor that homeopathy "worked instantly".*	
**CTCD**	It is true that doctors deny that homeopathy works mainly because they are coerced by large pharmaceutical companies
	It is false that doctors deny that homeopathy works mainly because they are coerced by large pharmaceutical companies
**CTCR**	It is true that doctors deny that homeopathy works mainly because they are coerced by large pharmaceutical companies
	It is true that doctors deny that homeopathy works mainly because it threatens their profit
**NCCD**	It is true that Rosa improved mainly because these infections commonly resolve with time, without medication
	It is false that Rosa improved mainly because these infections commonly resolve with time, without medication
**NCCR**	It is true that Rosa improved mainly because these infections commonly resolve with time, without medication
	It is true that Rosa improved mainly because she took antibiotics that addressed the cause
**[12]** *Both Yasmine and Nazim migrated to France as children to escape the Algerian civil war in the 1990s. After ten years of dating, they decided to try for a baby. They've been trying for nearly five years and have made regular visits to a specialized fertility clinic. Despite this, Yasmine's cycles are irregular and painful, and Nazim's semen analysis showed a low sperm count and reduced motility.*	
**CTCD**	It is true that there is a covert French government program aimed at sterilizing targeted populations via chemtrails (aircraft condensation trails)
	It is false that there is a covert French government program aimed at sterilizing targeted populations via chemtrails (aircraft condensation trails)
**CTCR**	It is true that there is a covert French government program aimed at sterilizing targeted populations via chemtrails (aircraft condensation trails)
	It is true that there is a covert French government program aimed at sterilizing targeted populations via medications secretly spiked to reduce fertility over time
**NCCD**	It is true that their fertility problems are mainly caused by their older age and early-life stress related to the war
	It is false that their fertility problems are mainly caused by their older age and early-life stress related to the war

Continued on next page

Table S6 – continued from previous page

**NCCR**	It is true that their fertility problems are mainly caused by their older age and early-life stress related to the war
	It is true that their fertility problems are mainly caused by war-related toxin exposure in childhood that later affected reproductive health
**[13]** *Emma is an exceptionally precise and meticulous top-tier scientist at CERN in Geneva (Switzerland). Her job is to run quality controls, ensuring the infrastructure stays safe and highly precise. During a nighttime checkup, Emma reported an unusual low-frequency "hum", but the instruments did not register anything. The next day, Emma had a mild headache and forgot that her partner was scheduled to pick up the kids from school, and she showed up as well.*	
**CTCD**	It is true that the hum was mainly caused by CERN running a human-influence experiment
	It is false that the hum was mainly caused by CERN running a human-influence experiment
**CTCR**	It is true that the hum was mainly caused by CERN running a human-influence experiment
	It is true that the hum was mainly caused by a French drone overflight on an undisclosed mission
**NCCD**	It is true that the hum was mainly caused by a planned routine maintenance running at night
	It is false that the hum was mainly caused by a planned routine maintenance running at night
**NCCR**	It is true that the hum was mainly caused by a planned routine maintenance running at night
	It is true that the hum was mainly caused by an accidental anomaly in the power-grid
**[14]** *In 2022, Professor Jonas Leitner's team at the University of Innsbruck (Austria) discovered a superconducting material that reduces energy dispersion to near zero. In their paper, published in the prestigious Physical Review Letters, the team reported a consumption-to-energy efficiency of 96.8%, far above the previous best results of around 57.2%. A year after publication, the paper was retracted.*	
**CTCD**	It is true that the data were genuine and accurate, but energy-industry lobbyists forced the journal to retract the article
	It is false that the data were genuine and accurate, but energy-industry lobbyists forced the journal to retract the article
**CTCR**	It is true that the data were genuine and accurate, but energy-industry lobbyists forced the journal to retract the article
	It is true that, like many scientists, Professor Leitner fabricated data to attract large amounts of public funding
**NCCD**	It is true that the retraction was mainly due to an accidental decimal-point error by a student, who reported 96.8% instead of 9.68%
	It is false that the retraction was mainly due to an accidental decimal-point error by a student, who reported 96.8% instead of 9.68%
**NCCR**	It is true that the retraction was mainly due to an accidental decimal-point error by a student, who reported 96.8% instead of 9.68%
	It is true that the retraction was mainly due to an accidental calibration error during data collection that inflated the efficiency estimate
**[15]** *Athens (Greece), 2024. Conservative Member of Parliament Eleni Karalis wakes to her phone buzzing with a link trending on Twitter. Her bank statement has been posted in full: deep overdrafts tied to a large mortgage, and late fees. By noon, tabloids call her financially reckless and a moral hypocrite. The bank blames "a breach" and publicly apologizes. Nevertheless, Eleni Karalis resigns and leaves politics.*	
**CTCD**	It is true that the leak was intentionally orchestrated by political opponents to force her resignation and trigger a by-election
	It is false that the leak was intentionally orchestrated by political opponents to force her resignation and trigger a by-election
**CTCR**	It is true that the leak was intentionally orchestrated by political opponents to force her resignation and trigger a by-election
	It is true that the leak was intentionally orchestrated by the bank, as usually done, to force rapid repayment via public stigma
**NCCD**	It is true that the leak was mainly the result of ordinary criminal hacking and data trading, unrelated to Eleni's politics
	It is false that the leak was mainly the result of ordinary criminal hacking and data trading, unrelated to Eleni's politics
**NCCR**	It is true that the leak was mainly the result of ordinary criminal hacking and data trading, unrelated to Eleni's politics
	It is true that the leak was mainly the result of an internal accidental mistake by a bank intern, unrelated to Eleni's politics
**[16]** *Denmark has more than 400 islands. Before 2010, only about 10% were linked by bridges, so the government launched a fast-track infrastructure program: in just two years, new bridges connected roughly 35%. The spending was massive (3% of GDP), but it became a point of national pride. In 2013, one of these new bridges collapsed during morning traffic, sending vehicles into the water and injuring 23 people.*	
**CTCD**	It is true that the collapse was engineered mainly to create a public crisis and unlock emergency infrastructure funds
	It is false that the collapse was engineered mainly to create a public crisis and unlock emergency infrastructure funds
**CTCR**	It is true that the collapse was engineered mainly to create a public crisis and unlock emergency infrastructure funds
	It is true that the collapse was engineered mainly to isolate low-income public-housing residents on the island and cut welfare costs
**NCCD**	It is true that the collapse was mainly caused by an engineering failure that accidentally went unnoticed during inspections
	It is false that the collapse was mainly caused by an engineering failure that accidentally went unnoticed during inspections
**NCCR**	It is true that the collapse was mainly caused by an engineering failure that accidentally went unnoticed during inspections
	It is true that the collapse was mainly caused by soil erosion that accidentally went unnoticed during project planning

Note. **CTCD**: conspiracy contradictory, **CTCR**: conspiracy contrary, **NCCD**: nonconspiracy contradictory, **NCCR**: nonconspiracy contrary.

SM12. Endorsement of items in Study 2

SM12.1. Endorsement of true/false non/conspiratorial explanations

To better understand the incoherence patterns we observed in the main analysis, we explored the endorsement of individual items (instead of pairs). In Figure S5, we show endorsement of items as a function of conspiracism split by explanation type (conspiracy [CT] vs nonconspiracy [NC]), logical relation (contrary [CR] vs contradictory [CD]), and whether they were framed as true or false. On average, endorsement of explanations was worldview-consistent: conspiracism correlated positively with conspiracy explanations framed as true (true CTCD $r = .44$, $p < .001$, true CTCR $r = .37$, $p < .001$) and negatively—although non-significantly—with nonconspiracy mundane explanations framed as true (true NCCD $r = -.09$, $p = .054$, true NCCR $r = -.07$, $p = .141$); the reverse pattern was found when explanations were framed as false (false CTCD $r = -.35$, $p < .001$, false NCCD $r = .20$, $p < .001$; see Figure S5). These patterns validate that participants responded to the content of the explanations in a manner consistent with their conspiratorial worldview, rather than responding randomly or uniformly (see also Section SM10).

SM12.2. Endorsement of conspiracy vs rejection of nonconspiracy explanations

To what extent is conspiracism associated with the rejection of mundane explanations versus the acceptance of conspiratorial explanations? Our contradictory pairs provide the opportunity to simultaneously test the endorsement of a statement framed either as true or false. Here, we recoded contradictory items' endorsement to align with the conspiratorial view. Endorsement was flipped ($1 -$ endorsement) when conspiracy explanations were framed as false and when nonconspiracy explanations were framed as true. In Figure S6, we show the fitted lines of items' endorsement by conspiracism (models estimated using the following formula: `lmer(endorsement ~ CMQ + explanation_type + (1 | PID), data = only_contradictory_pairs)`) for both raw (left panel) and recoded (right panel) endorsement scores.

When the endorsement of a conspiracy explanation framed as false is flipped to reflect endorsement when true, the slope of conspiratorial explanations moves from $\beta = .12$ [-.04, .28] to $\beta = 1.39$ [1.19, 1.59]. Likewise, if conspiracism is associated with the rejection of mundane and accidental causes, recoding the endorsement of nonconspiracy explanations framed as true should capture their rejection. The slope of nonconspiratorial explanations increased from $\beta = .14$ [-.02, .30] to $\beta = .40$ [.20, .59], indicating that conspiracism is also associated with the rejection of mundane causes. In the recoded model, the interaction effect was significant ($\beta = .20$, $SE = .02$, $t = 9.58$, $p < .001$), indicating that the slope of endorsement by conspiracism was steeper for conspiratorial than for nonconspiracy explanations. At the same time, the intercept was lower for conspiracy than for nonconspiracy explanations ($\beta = -.23$, $SE = .02$, $t = -10.85$, $p < .001$), suggesting that rejection of mundane explanations was relatively widespread even among participants low in conspiracism, whereas endorsement of conspiratorial explanations was more selectively tied to higher levels of conspiracism. In other words, conspiracism differentiates people more sharply in what they accept than in what they reject.

Overall, this recoding reveals an asymmetry between a strong endorsement of conspiratorial explanations and a weaker rejection of mundane ones (somewhat paralleled in the non-significant negative correlations for nonconspiracy explanations shown in Figure S5). Conspiracism appears primarily concerned with accepting alternative conspiratorial accounts, or possibly raising their perceived likelihood, rather than with dismissing mundane or accidental explanations.

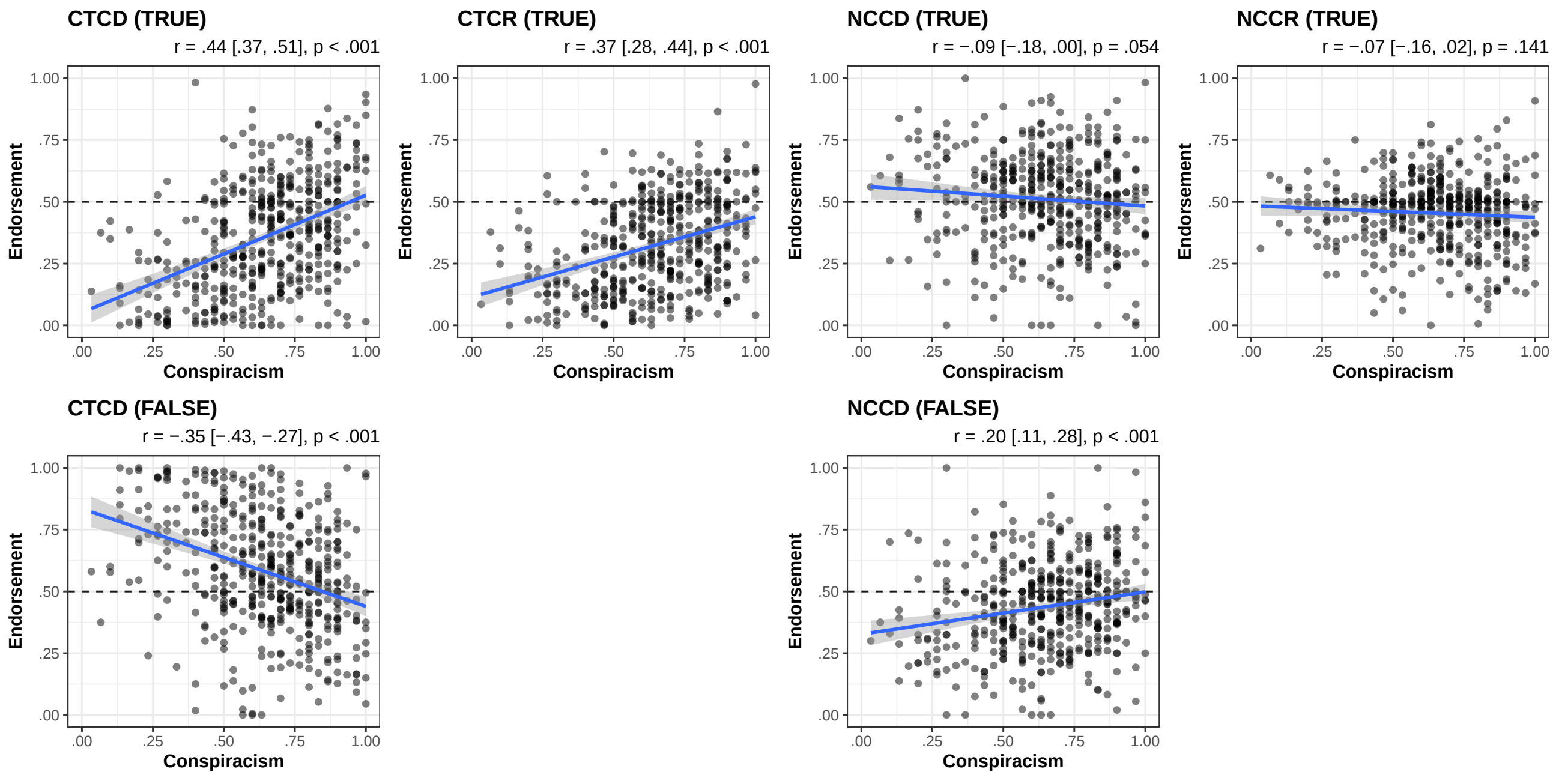

Figure S5. Endorsement of individual items as a function of conspiracism (CMQ), split by condition and true/false framing. Top row: items framed as true across all four conditions. Bottom row: items framed as false (available only for contradictory pairs). Each dot represents a participant's averaged endorsement across items of the corresponding type. The dashed line indicates the midpoint (.50) endorsement. CTCD: conspiracy contradictory; CTCR: conspiracy contrary; NCCD: nonconspiracy contradictory; NCCR: nonconspiracy contrary.

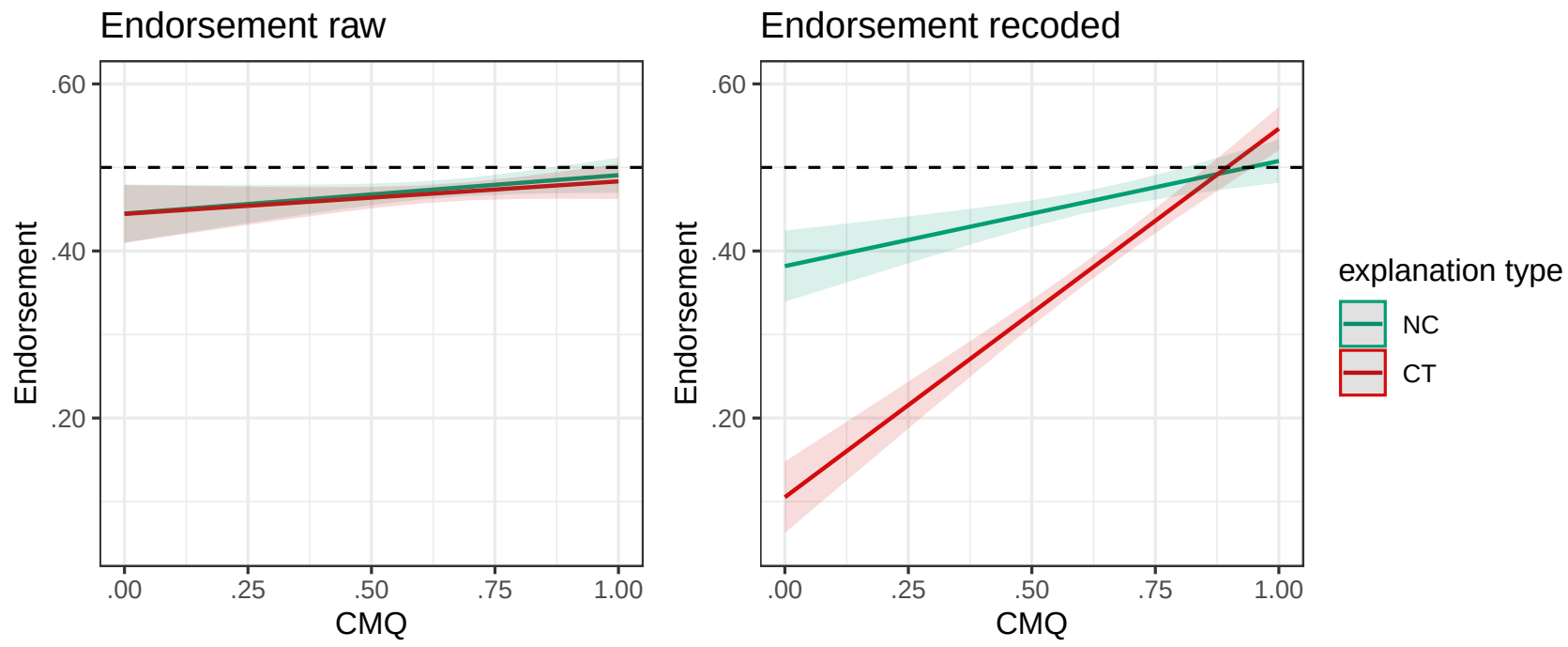

Figure S6. Raw (left) and recoded (right) endorsement by conspiracism (CMQ) for conspiratorial (CT) and nonconspiratorial (NC) explanations in contradictory pairs. Recoded endorsement was flipped (1 − endorsement) when conspiracy explanations were framed as false and when nonconspiracy explanations were framed as true, so that higher values consistently reflect worldview-consistent responding. The dashed line indicates the midpoint (.50) endorsement. Shaded ribbons represent 95% confidence intervals.

SM13. Analytical strategy and deviation from preregistration in Study 2

Incoherence was computed as the Euclidean distance to the coherence boundary. Given two incompatible statements (A and B), contrary incoherence is computed as the positive distance from 1 (see Section SM4), whereas contradictory incoherence is computed as any deviation from $A + B = 1$, as shown in Equation S3.

$$I(A, B) = \frac{|A + B - 1|}{\sqrt{2}} \tag{S3}$$

We preregistered two research questions: RQ1: Does incoherence generalize to nonconspiracy content? RQ2: Does incoherence generalize to contradictory pairs? To address RQ1, we planned to fit a linear mixed-effects regression (Model 1) in which incoherence scores are predicted by CMQ, and to test whether this association differs by explanation type by including the interaction between CMQ and explanation type, with a random intercept for participants and a random slope for CMQ. To address RQ2, we specified the same model (Model 2), restricting the analysis to responses to contradictory pairs.

We deviated from this preregistered model specification by dropping the random slope for CMQ. This was necessary because CMQ is a between-participant predictor and therefore does not vary within participants; consistently, models including both a random intercept and a random slope for CMQ failed to converge. We therefore simplified the random-effects structure to include only a random intercept for participants.

We also recognized that the preregistered model specifications—which included an interaction with explanation type—do not directly address the research questions stated in the preregistration (i.e., generalization to nonconspiracy content and to contradictory pairs). These research questions were instead addressed using two simpler models discussed in the main text: we fitted two separate zero-inflated Gamma models predicting incoherence from conspiracism, with a random intercept for participants, restricting the data to the relevant subset (nonconspiracy pairs for RQ1; contradictory pairs for RQ2). To examine whether the effect of conspiracism on incoherence differed by explanation type and by logical relation, we fitted two additional models on the full dataset (also discussed in the main text), each including an interaction between conspiracism and the relevant factor (model 1: explanation type; model 2: logical relation). Because these models diverge from the preregistered ones, we additionally report the coefficients from the two preregistered models in Table S8 for transparency.

As in Study 1, because the dependent variable (incoherence) contained a large proportion of zeros (54.67%), we compared a Gaussian mixed model with a zero-inflated Gamma mixed model (see Table S7). Residual diagnostics indicated substantial misfit for the Gaussian specification, including strong zero-inflation, non-uniform residuals, and many outliers. In contrast, the zero-inflated Gamma model showed no evidence of residual zero-inflation, acceptable residual uniformity, and no outlier problems, suggesting a substantially better fit to the observed outcome distribution. We therefore retained the zero-inflated Gamma model, fitted using the *glmmTMB* package in R [13] and specified in Code 2.

Code 2. R

```
m <- glmmTMB(i ~ CMQ * explanation_type * logical_relation + (1 | PID),
             ziformula = ~ CMQ * explanation_type * logical_relation + (1 | PID),
             family = ziGamma(link = 'log'),
             data)
```

Table S7. Model comparison: Gaussian vs zero-inflated Gamma mixed models predicting incoherence in Study 2.

	Gaussian	ZI-Gamma
Marginal R^2	.034	.056
Conditional R^2	.211	.372
Dispersion (ratio)	1.00, p = .936	.62, p < .001
testZeroInflation (ratioObsSim)	Inf, p < .001	.99, p = .732
Outliers	N = 206, p < .001	N = 11, p = .365
Uniformity	D = .24, p < .001	D = .01, p = .265
Num. obs.	7479	7479
Num. groups: PID	469	469
Var: PID (Intercept)	0.00	
Var: Residual	0.02	
Var (count model): PID (Intercept)		0.29
Var (zero model): PID (Intercept)		2.61

Table S8. Two zero-inflated Gamma models as preregistered: **Model 1** predicting incoherence (*I* scores) from conspiracism (CMQ) and explanation type across all pairs; **Model 2** restricted to contradictory pairs.

		**Model 1** (all pairs)						**Model 2** (contradictory pairs)					
part	term	b	[95%CI]	OR	[95%CI]	r	p	b	[95%CI]	OR	[95%CI]	r	p
**Full Model**													
cond	(Intercept)	-1.77	[-2.00, -1.54]				***	-2.18	[-2.47, -1.88]				***
cond	CMQ	-.20	[-.53, .14]					.08	[-.34, .51]				
cond	explanation_typeCT	-.44	[-.68, -.20]				***	-.11	[-.39, .16]				
cond	CMQ:explanation_typeCT	.56	[.22, .91]				**	.16	[-.23, .56]				
zi	(Intercept)	-.73	[-1.18, -.29]				**	-.35	[-1.28, .58]				
zi	CMQ	.99	[.33, 1.65]				**	2.42	[1.04, 3.81]				***
zi	explanation_typeCT	-1.20	[-1.56, -.84]				***	-.57	[-1.18, .04]				.
zi	CMQ:explanation_typeCT	1.28	[.75, 1.80]				***	.70	[-.20, 1.61]				
**Individual Slopes**													
cond	CT	.37	[.01, .73]	1.45	[1.01, 2.08]	.10	*	.25	[-.19, .69]	1.28	[.83, 1.99]	.07	
cond	NC	-.20	[-.53, .14]	.82	[.59, 1.15]	-.05		.08	[-.34, .51]	1.09	[.71, 1.66]	.02	
zi	CT	2.27	[1.59, 2.94]	9.66	[4.91, 19.00]	.53	***	3.13	[1.74, 4.52]	22.84	[5.69, 91.62]	.65	***
zi	NC	.99	[.33, 1.65]	2.69	[1.39, 5.21]	.26	**	2.42	[1.04, 3.81]	11.28	[2.83, 44.96]	.56	***

Note. Estimates for the ZI component are sign-reversed to show the probability of incoherence (i.e., non-zero *I* scores). The *r* coefficient is computed from OR. **CT**: conspiracy; **NC**: nonconspiracy; **CR**: contrary; **CD**: contradictory.

SM14. Additional Figures and Tables for Study 2

In Figure S7, left panel, we plot participants' aggregated I scores as a function of conspiracism (CMQ, [14]). When I scores were aggregated across conditions, they correlated weakly with conspiracism ($r_{467} = .15$, $[.07, .24]$, $p < .001$). In Figure S7, right panel, we plot participants' aggregated I scores as a function of conspiracism split by conditions. Conspiracy explanations yielded positive associations between incoherence and conspiracism, whereas nonconspiracy explanations were nonsignificant.

In Table S9, we provide the full model details and individual slopes for each condition for the main analysis of Study 2. In Table S10, we provide the full model details and individual slopes for each condition for the analyses using the contradictory incoherence band extended by 5%.

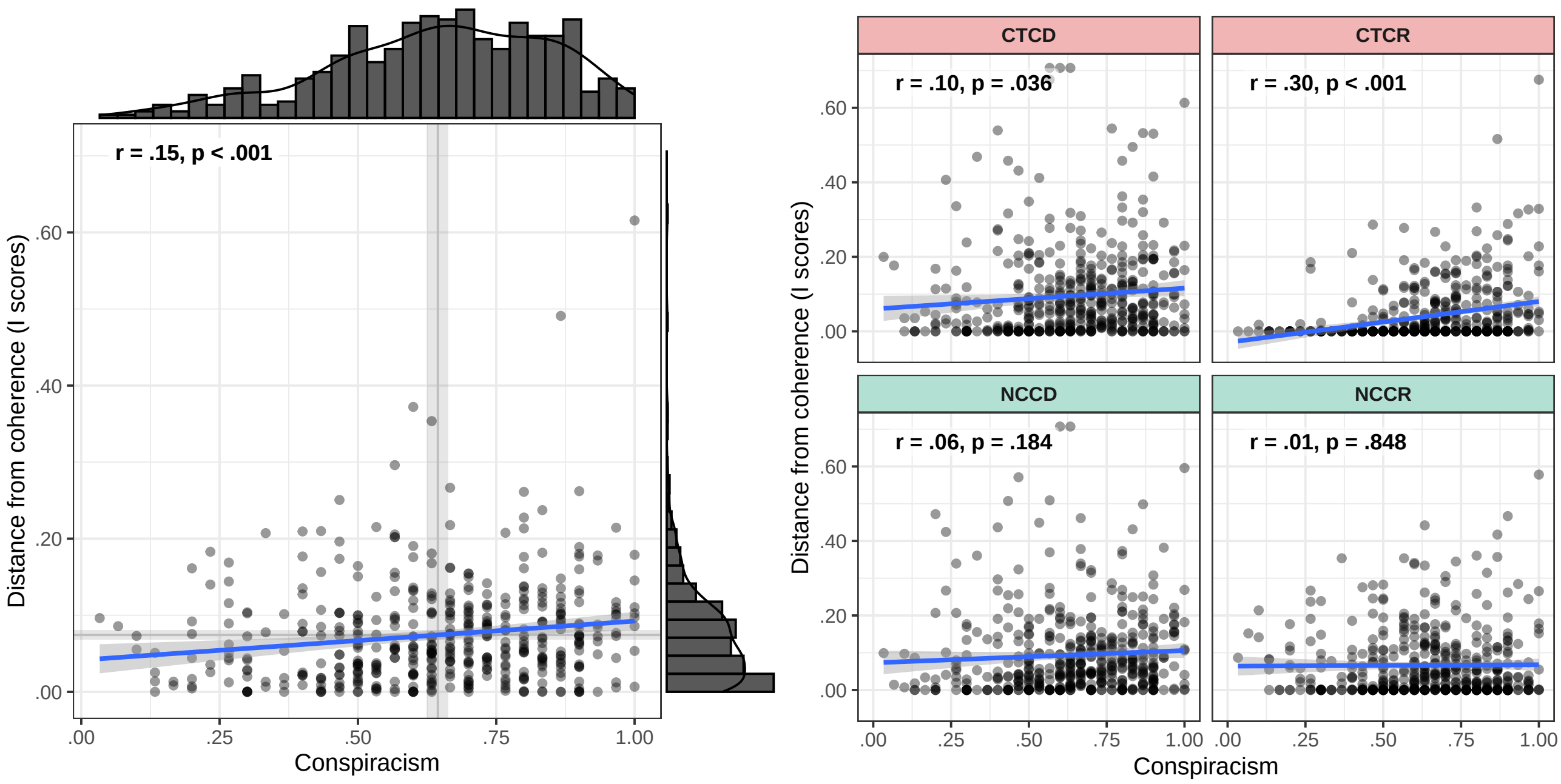

Figure S7. Scatter plot and distributions of participants' aggregated incoherence scores as a function of conspiracism (CMQ). Left panel: aggregated I scores across all conditions. Vertical and horizontal lines with shaded ribbons indicate the means and 95% confidence intervals for conspiracism and incoherence, respectively. Marginal histograms show the distributions of each variable. Right panels: I scores split by explanation type (conspiracy, top row; nonconspiracy, bottom row) and logical relation (contradictory, left column; contrary, right column). CTCD: conspiracy contradictory; CTCR: conspiracy contrary; NCCD: nonconspiracy contradictory; NCCR: nonconspiracy contrary.

Table S9. Three-way interaction model details and individual slopes for Study 2

part	term	b [95%CI]		OR [95%CI]		r	p
**Full Model**							
cond	(Intercept)	-1.32	[-1.64, -1.00]				***
cond	CMQ	-.44	[-.91, .02]				.
cond	explanation_typeCT	-.88	[-1.40, -.36]				***
cond	logical_relationCD	-.81	[-1.15, -.47]				***
cond	CMQ:explanation_typeCT	1.15	[.44, 1.86]				**
cond	CMQ:logical_relationCD	.52	[.03, 1.01]				*
cond	explanation_typeCT:logical_relationCD	.79	[.20, 1.38]				**
cond	CMQ:explanation_typeCT:logical_relationCD	-1.03	[-1.84, -.21]				*
zi	(Intercept)	-1.36	[-1.98, -.73]				***
zi	CMQ	.50	[-.42, 1.42]				
zi	explanation_typeCT	-3.48	[-4.19, -2.77]				***
zi	logical_relationCD	1.00	[.46, 1.54]				***
zi	CMQ:explanation_typeCT	3.82	[2.83, 4.80]				***
zi	CMQ:logical_relationCD	1.33	[.53, 2.13]				**
zi	explanation_typeCT:logical_relationCD	3.08	[2.20, 3.95]				***
zi	CMQ:explanation_typeCT:logical_relationCD	-3.32	[-4.58, -2.06]				***
**Individual Slopes**							
cond	CTCR	.71	[.05, 1.37]	2.03	[1.06, 3.92]	.19	= .034
cond	CTCD	.20	[-.20, .59]	1.22	[.82, 1.81]	.05	= .323
cond	NCCR	-.44	[-.91, .02]	.64	[.40, 1.02]	-.12	= .063
cond	NCCD	.07	[-.31, .45]	1.08	[.74, 1.57]	.02	= .704
zi	CTCR	4.32	[3.22, 5.41]	74.85	[24.93, 224.71]	.77	< .001
zi	CTCD	2.32	[1.42, 3.23]	10.22	[4.12, 25.34]	.54	< .001
zi	NCCR	.50	[-.42, 1.42]	1.65	[.66, 4.14]	.14	= .288
zi	NCCD	1.83	[.92, 2.73]	6.22	[2.52, 15.39]	.45	< .001

Note. cond: conditional model; zi: zero-inflation model. Estimates for ZI component are sign reversed so to show the non-zero probability (i.e., incoherence).

Table S10. Three-way interaction model details and individual slopes for Study 2 with contradictory incoherence extended by 5%

part	term	b [95%CI]		OR [95%CI]		r	p
**Full Model**							
cond	(Intercept)	-1.47	[-1.73, -1.21]				***
cond	CMQ	-.31	[-.68, .07]				
cond	explanation_typeCT	-.73	[-1.15, -.30]				***
cond	logical_relationCD	-.23	[-.52, .07]				
cond	CMQ:explanation_typeCT	.99	[.41, 1.57]				***
cond	CMQ:logical_relationCD	.24	[-.19, .67]				
cond	explanation_typeCT:logical_relationCD	.72	[.22, 1.23]				**
cond	CMQ:explanation_typeCT:logical_relationCD	-.93	[-1.62, -.23]				**
zi	(Intercept)	-1.14	[-1.65, -.63]				***
zi	CMQ	.39	[-.37, 1.14]				
zi	explanation_typeCT	-3.09	[-3.75, -2.44]				***
zi	logical_relationCD	.04	[-.46, .54]				
zi	CMQ:explanation_typeCT	3.38	[2.46, 4.29]				***
zi	CMQ:logical_relationCD	.98	[.24, 1.72]				**
zi	explanation_typeCT:logical_relationCD	2.78	[1.96, 3.60]				***
zi	CMQ:explanation_typeCT:logical_relationCD	-3.05	[-4.22, -1.88]				***
**Individual Slopes**							
cond	CTCR	.68	[.15, 1.22]	1.98	[1.16, 3.39]	.18	*
cond	CTCD	-.00	[-.36, .35]	1.00	[.70, 1.42]	-.00	
cond	NCCR	-.31	[-.68, .07]	.74	[.51, 1.07]	-.08	
cond	NCCD	-.07	[-.41, .27]	.94	[.67, 1.32]	-.02	
zi	CTCR	3.77	[2.84, 4.69]	43.20	[17.12, 109.03]	.72	***
zi	CTCD	1.70	[.95, 2.44]	5.46	[2.59, 11.50]	.42	***
zi	NCCR	.39	[-.37, 1.14]	1.48	[.69, 3.14]	.11	
zi	NCCD	1.37	[.63, 2.10]	3.93	[1.88, 8.21]	.35	***

Note. Estimates for ZI component are sign reversed so to show the non-zero probability (i.e., incoherence).

SM15. Contextualize contrary conspiracy pairs of Study 2 with previous studies

We subset the contrary-conspiracy pairs from our Study 2 and compared the effect sizes and distributions with those extracted from the contrary-conspiracy pairs already analyzed in Study 1. In Figure S8, we report a mini-meta-analysis (Panel A, top), the scatter plots of incoherence by conspiracism per each study (Panel B, bottom left), as well as descriptive statistics about the I scores, specifically the percentage of incoherent responses, the mean of I scores when $I > 0$ and for the whole responses when $I \geq 0$ (Panel C, bottom right).

As for the mini meta-analysis (forest plots in Figure S8, Panel A), for each study, we fitted a zero-inflated Gamma model predicting incoherence scores from conspiracism, with random intercepts for participants. We then conducted random-effects meta-analyses (using the R package *metafor*, [15]), extracting the b coefficients from both the conditional and zero-inflation components. The pooled effect of conspiracism on the magnitude of incoherence (conditional component) was $b = .36$ [.22, .49], $z = 5.10$, $p < .001$ ($I^2 = 45.40\%$, $Q(8) = 14.15$, $p = .078$). The pooled effect of conspiracism on the probability of being incoherent (zero-inflation component, sign-reversed) was $b = 5.17$, [4.38, 5.96], $z = 12.80$, $p < .001$ ($I^2 = 86.40\%$, $Q(8) = 62.59$, $p < .001$). As for the associations between I scores and conspiracism (shown in the scatter plots in Figure S8, Panel B), Study 2 was not visually distinguishable from the other samples, with a mid-range correlation coefficient.

Study 2 exhibited the largest conditional effect among all samples ($b = 1.00$), yet a comparatively moderate zero-inflation effect ($b = 4.35$, below the pooled estimate of $b = 5.17$). The descriptive statistics of I (Figure S8, Panel C) help explain this dissociation between the two model components. Study 2 had the second-highest proportion of incoherent responses (20.9%), yet the lowest average incoherence among those participants ($M = .197$), and a mid-range level of overall incoherence ($M = .041$). In other words, Study 2 tracked many participants who were just barely incoherent, but relatively few with large deviations. Four design choices of Study 2 help explain these results.

First, the 0–100 continuous probability scale is far more granular than the 4- or 5-point Likert scales used in previous studies. Especially in situations of uncertainty when approximately 50% is allocated to competing hypotheses, allocating more certainty to one hypothesis means moving from *neither agree nor disagree* to *agree* (by .25 or .20 points), requiring a relatively substantial commitment. With a 101-point scale, less commitment is required: someone uncertain who rates two contrary claims at 51% each is captured as incoherent. This finer measurement resolution inflates the base rate of binary incoherence while producing many near-zero incoherent responses.

Second, unlike previous studies in which participants rated isolated statements (e.g., *"Diana was murdered by MI6"*), we embedded incompatible claims within vignettes depicting realistic but fictitious events, and framed each contrary claim as an explanation for the event (e.g., *Rate the likelihood of... It is true that...*). This design choice likely affected both model components. On the one hand, presenting claims as explanations within a scenario aligns with the epistemic function of conspiracism: conspiracy theories provide an explanatory framework for making sense of ambiguous or threatening events. This explanatory mode may have amplified the worldview-driven differentiation in the degree of incoherence, driving a steeper conditional slope. On the other hand, the two conspiracy explanations were the only two accounts provided to participants when the conspiracy condition was randomly allocated. Because the vignettes described realistic and uncertain situations accompanied by plausible —albeit conspiratorial— explanations, participants across the conspiracism spectrum may have found it reasonable to entertain both conspiratorial accounts in the absence of alternative explanations. In the presence of uncertainty, a participant leaning toward the uncertain midpoint (e.g., 50%) would need only a slight tilt toward one explanation (e.g., 51% + 50%) to cross the coherence boundary. This plausibility-induced endorsement would have inflated the base rate of incoherence, contributing to the attenuated zero-inflation effect.

Third, the fictitious scenarios eliminate prior knowledge and familiarity effects. In previous studies using real-world conspiracies (Diana, 9/11, COVID), participants high in conspiracism had likely been exposed to multiple competing narratives, which may have inflated endorsement through familiarity. By relying on fictitious scenarios, we isolated the worldview-driven component of incoherence from the "knowledge" component, yielding a purer but weaker effect. This explains why average I scores were in the lower range compared to studies in which the familiarity component contributed to incoherence alongside the worldview-driven component.

Fourth, we explicitly made the logical structure of each pair highly transparent (prefacing claims with *"It is true that..."*), reframing the task from attitude expression to propositional truth evaluation. This made it harder for participants to compartmentalize the two claims, and likely restrained participants from the extreme joint endorsement observed in more naturalistic formats; this would have further compressed the distribution of I scores. Crucially, however, this design choice did not prevent high-conspiracism participants from being more incoherent than their low-conspiracism counterparts, preserving the conditional slope.

In conclusion, the design choices in Study 2 raised sensitivity to detect any departure from coherence (producing a higher proportion of incoherent responses and flattening the zero-inflation component) while allowing worldview-driven conspiracism—cleaned of prior knowledge and familiarity confounds—to emerge as the primary driver of the degree of incoherence (strengthening the conditional effect).

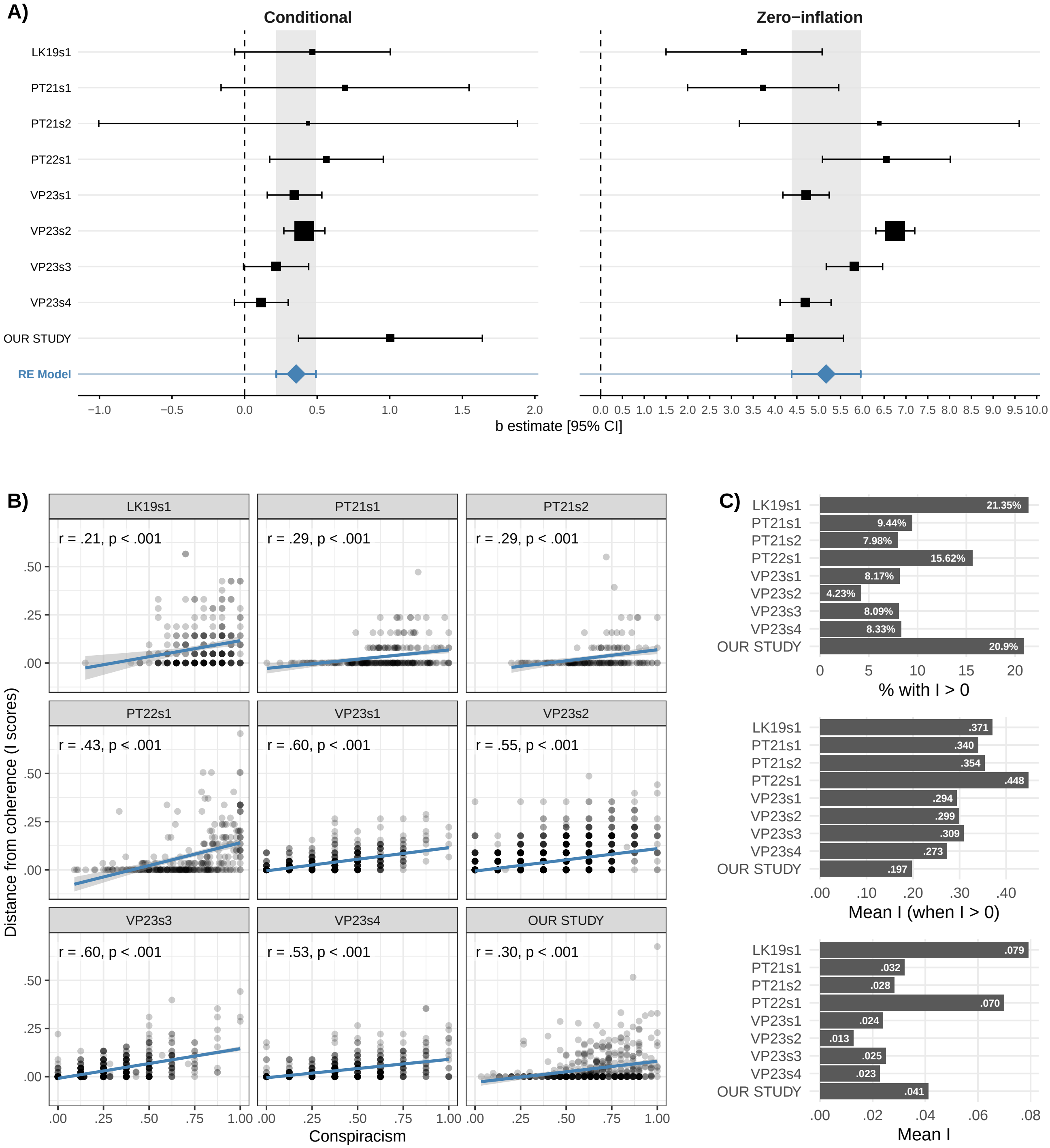

Figure S8. Contextualizing contrary-conspiracy pairs from Study 2 with Study 1 samples. Panel A: Forest plots of the conditional component (left) and zero-inflation component (right) from zero-inflated Gamma models predicting incoherence from conspiracism in each sample. The blue diamond indicates the pooled random-effects estimate (error bars and shaded area correspond to the 95% confidence interval). Panel B: Scatter plots of incoherence (I scores) by conspiracism for each sample, with fitted regression lines (and 95% confidence bands). Panel C: Descriptive statistics of I scores across samples. Top: percentage of responses with $I > 0$. Middle: mean I score among incoherent responses ($I > 0$). Bottom: overall mean I score.

SM16. Survey

Preview as participant

Interpreting events in Europe's recent history

By Alessandro Miani
£1.50 • £9.00/hr
10 mins
500 places
Survey

This study investigates how people interpret events or situations from Europe's recent history.

You will first complete a short 21-question section about your worldview. Then, you will read **16 short vignettes** describing events or situations in Europe's recent history. For each vignette, you will see two statements (selected at random from several possible statement pairs) that offer possible explanations or context for the event.

Your task is to rate how likely each statement is to be true using a 0–100 probability scale (0 = certainly false, 100 = certainly true).

Please do not look anything up. Answer based on your best judgment, even if you are unsure. The survey will take approximately 10 minutes.

Note. We use missing responses as an attention/engagement check. If you leave **3 or more** items unanswered, we may ask you (via Prolific) to **return your submission** rather than approving it.

~

Participant Information Sheet

Background. You are invited to participate in a study conducted by Dr. Alessandro Miani as part of a research project within the University of Bristol's School of Psychology. This study aims to explore how worldviews affect the words that come to mind.

Study Sponsorship and Approval. This project is funded by the Swiss National Science Foundation and has been approved by the University of Bristol Workstream One Research Ethics Committee.

Study Procedure. The study consists of two parts. You will first complete a short 21-question section about your worldview. Then, you will read 16 short vignettes describing events or situations in Europe's recent history. For each vignette, you will see two statements (selected at random from several possible statement pairs) that offer possible explanations or context for the event. Your task is to rate how likely each statement is to be true using a 0–100 probability scale (0 = certainly false, 100 = certainly true). Participation typically takes about 10 minutes, and it is strictly anonymous. Your

effort in providing thoughtful responses is crucial to the success of this study. There are no right or wrong answers: our goal is simply to understand how you explain events.

Risks to Participants. There are no known risks associated with participating in this study. You are free to withdraw at any time without giving a reason by simply closing the browser window. Participation is entirely voluntary, and there are no penalties if you choose not to take part. Please note that, due to the anonymous nature of the study, it will not be possible to withdraw your data once it has been submitted.

Data Handling. Your data will remain confidential because it will be anonymized. This means you will be assigned an identification number, and any personally identifiable information will be removed. As a result, it will not be possible to identify you from any of the study's documentation or results. At the end of the study, the anonymized data will be made available as "Open Access", meaning it will be stored in an online database and accessible to the public.

What is Open Data? Open data refers to data that is made freely available to anyone who is interested in the research or wishes to conduct their own analysis. While we will have no control over how this data is used, all data will be anonymized before it is shared. Therefore, you cannot be personally identified from the research data.

Why Open Data? Sharing research data and findings is considered best practice in science and is often required by funding agencies and academic journals. Since a large proportion of research is publicly funded, it is important that the results are made publicly accessible. Making data open encourages wider use, maximizes the impact of the research, and helps generate new research opportunities. By participating in this study, you agree that the data collected from you will be anonymized and made available as open data. You understand that this anonymized data may be used for purposes unrelated to this study, but that it will not be possible to identify you from it.

Disclosure of Information in Studies. Please note that in many psychology experiments, full disclosure of the research aims is not provided upfront. This is done to preserve the integrity of the study, as knowing too much about the research questions in advance may influence how participants think, behave, and respond during the experiment.

Contacts. If you have any questions about this study, please contact Dr. Alessandro Miani at alessandro.miani@bristol.ac.uk.

~

Consent Form

By consenting to participate, you confirm that:

- You are 18 years old or over.
- You have received sufficient information about the study to make an informed decision about your participation.

By consenting to participate, you also understand that:

- You are free to withdraw from the study at any time by closing the browser window.
- You do not need to provide a reason for withdrawing.

If you have any concerns regarding your participation in this study please direct them to the University of Bristol Workstream One Research Ethics Committee (research-governance@bristol.ac.uk).

Clicking the 'NEXT' button indicates your consent to participate in this study. If you do not wish to participate, simply close the browser tab at any time.

Devices you can use to take this study:

- Desktop
- Mobile
- Tablet

Open study link in a new window

qualtrics XM

Survey Flow

Standard: EIS (1 Question)
Standard: INIT (1 Question)

EmbeddedData
cond_countsValue will be set from Panel or URL.
cond_sequenceValue will be set from Panel or URL.
DeviceIdentifierValue will be set from Panel or URL.

Group: Untitled Group

BlockRandomizer: 16 -

Block: CHLOE (5 Questions)
Standard: VACCINES (5 Questions)
Standard: RESTAURANT (5 Questions)
Standard: SUBLIMINAL (5 Questions)
Standard: CANCER (5 Questions)
Standard: VALDORIA (5 Questions)
Standard: SINGER (5 Questions)
Standard: AIDS (5 Questions)
Standard: ADHD (5 Questions)
Standard: UFO (5 Questions)
Standard: HOMEOPATHY (5 Questions)
Standard: FERTILITY (5 Questions)
Standard: CERN (5 Questions)
Standard: ENERGY (5 Questions)
Standard: LEAK (5 Questions)
Standard: BRIDGE (5 Questions)

Standard: DEBRIEFING (1 Question)

Page Break

qualtrics XM

Start of Block: EIS

JS

qualtrics XM

EIS Below are a number of statements about how people form beliefs and decide what is true. There are no right or wrong answers—please respond based on your own views. Please indicate how much you agree or disagree with each statement, using the scale from **1 (Strongly disagree)** to **7 (Strongly agree)**.

	1 (1)	2 (2)	3 (3)	4 (4)	5 (5)	6 (6)	7 (7)
I trust my gut to tell me what's true and what's not (1)	○	○	○	○	○	○	○
I trust my initial feelings about the facts (2)	○	○	○	○	○	○	○
My initial impressions are almost always right (3)	○	○	○	○	○	○	○
I can usually feel when a claim is true or false even if I can't explain how I know (4)	○	○	○	○	○	○	○
Evidence is more important than whether something feels true (5)	○	○	○	○	○	○	○
A hunch needs to be confirmed with data (6)	○	○	○	○	○	○	○
I trust the facts, not my instincts, to tell me what is true (7)	○	○	○	○	○	○	○

qualtrics XM

I need to be able to justify my beliefs with evidence (8)	○	○	○	○	○	○	○
Facts are dictated by those in power (9)	○	○	○	○	○	○	○
What counts as truth is defined by power (10)	○	○	○	○	○	○	○
Scientific conclusions are shaped by politics (11)	○	○	○	○	○	○	○
“Facts” depend on their political context (12)	○	○	○	○	○	○	○
I think that many very important things happen in the world, which the public is never informed about (13)	○	○	○	○	○	○	○
I think that politicians usually do not tell us the true motives for their decisions (14)	○	○	○	○	○	○	○
I think that government agencies	○	○	○	○	○	○	○

closely monitor all citizens (15)							
I think that events which superficially seem to lack a connection are often the result of secret activities (16)	○	○	○	○	○	○	○
I think that there are secret organizations that greatly influence political decisions (17)	○	○	○	○	○	○	○
I only believe people who make sure to be well-informed before saying what they think about a topic (18)	○	○	○	○	○	○	○
I only believe a statement if it is based on empirical evidence (19)	○	○	○	○	○	○	○
People should always stick to the facts and set aside personal opinions (20)	○	○	○	○	○	○	○

qualtrics XM

Before making a claim, people must first make sure they have acquired all the correct information (21)	○	○	○	○	○	○	○
This is a quality check. Please select 5 (22)	○	○	○	○	○	○	○

End of Block: EIS

Start of Block: INIT

JS

INIT In the next pages, you'll read 16 short descriptions of events or situations happened in Europe. After each vignette, you will see two statements that offer possible explanations or context. These two statements are randomly selected from a larger set, so different participants may see different statement pairs. For each statement, rate how likely you think it is to be true using a 0–100 probability scale: 0 = certainly false 100 = certainly true Please do not look anything up. Base your ratings on what you know or seems plausible to you. There are no right or wrong answers: we're interested in your honest response. Please try to **answer every question**. We use missing responses as an attention/engagement check. If you leave **3 or more** items unanswered, we may ask you (via Prolific) to **return your submission** rather than approving it. Click next to start with the first Vignette.

End of Block: INIT

Start of Block: CHLOE

JS

Chloe is an 8-year-old girl and a promising young skier. Her father, an influential member of the European Parliament, often takes her skiing in the Swiss Alps. One day, Chloe separated from her group during ski school and did not return home. Her father asked the European Union for

qualtrics XM

funds to support a search for her, triggering negative reactions from colleagues and opponents. After three days, Chloe returned home.

CHLOE_CTCD Probability (0–100):

	0 10 20 30 40 50 60 70 80 90 100
It is true that Chloe has been secretly held by her father in order to misuse public funds ()	
It is false that Chloe has been secretly held by her father in order to misuse public funds ()	

CHLOE_CTCR Probability (0–100):

	0 10 20 30 40 50 60 70 80 90 100
It is true that Chloe has been secretly held by her father in order to misuse public funds ()	
It is true that Chloe escaped an environment in which she was secretly forced into child labor ()	

CHLOE_NCCD Probability (0–100):

	0 10 20 30 40 50 60 70 80 90 100
It is true that Chloe accidentally fell from a nearby cliff ()	
It is false that Chloe accidentally fell from a nearby cliff ()	

qualtrics XM

CHLOE_NCCR Probability (0–100):

	0 10 20 30 40 50 60 70 80 90 100
It is true that Chloe accidentally fell from a nearby cliff ()	
It is true that Chloe was caught in a sudden avalanche ()	

End of Block: CHLOE

Start of Block: VACCINES

JS

A few days before his fourth birthday in July 2021, Lucas was kept home from kindergarten because he had a high fever. On the day of his birthday, the fever had not gone down, and he was rushed to the hospital. Later that day, doctors had to place Lucas in a medically induced coma to help him recover from severe brain inflammation (encephalitis).

VACCINES_CTCD Probability (0–100):

	0 10 20 30 40 50 60 70 80 90 100
It is true that the encephalitis was caused mainly by the COVID-19 vaccine administered a month earlier ()	
It is false that the encephalitis was caused mainly by the COVID-19 vaccine administered a month earlier ()	

VACCINES_CTCR Probability (0–100):

0 10 20 30 40 50 60 70 80 90 100

qualtrics XM

It is true that the encephalitis was caused mainly by the COVID-19 vaccine administered a month earlier ()	
It is true that the encephalitis was caused mainly by the MMR vaccine administered a month earlier ()	

VACCINES_NCCD Probability (0–100):

	0 10 20 30 40 50 60 70 80 90 100
It is true that the encephalitis was caused mainly by accidental food poisoning after eating his mum's tiramisu ()	
It is false that the encephalitis was caused mainly by accidental food poisoning after eating his mum's tiramisu ()	

VACCINES_NCCR Probability (0–100):

	0 10 20 30 40 50 60 70 80 90 100
It is true that the encephalitis was caused mainly by accidental food poisoning after eating his mum's tiramisu ()	
It is true that the encephalitis was caused mainly by an infected cut Lucas got accidentally in his granddad's garage ()	

End of Block: VACCINES

Start of Block: RESTAURANT

JS

qualtrics XM

John, a longtime heavy smoker, drinker, and recreational drug user, owns a multinational chain of fine-dining restaurants that caters to high society, including politicians and other influential people. He planned to launch a political career, claiming he knew many secrets. At the public presentation of his new party, an attendee shouted "They also know your secrets!". A few days later, he was found unconscious in the basement of one of his restaurants in London. He died in the hospital a few days after that.

RESTAURANT_CTCD Probability (0–100):

	0 10 20 30 40 50 60 70 80 90 100
It is true that John was murdered by political opponents ()	
It is false that John was murdered by political opponents ()	

RESTAURANT_CTCR Probability (0–100):

	0 10 20 30 40 50 60 70 80 90 100
It is true that John was murdered by political opponents ()	
It is true that John attempted suicide and died ()	

RESTAURANT_NCCD Probability (0–100):

0 10 20 30 40 50 60 70 80 90 100

Page 10 of 33

qualtrics XM

It is true that John died of an accidental heart attack caused mainly by physical exertion while carrying heavy food ()	
It is false that John died of an accidental heart attack caused mainly by physical exertion while carrying heavy food ()	

RESTAURANT_NCCR Probability (0–100):

	0	10	20	30	40	50	60	70	80	90	100
It is true that John died of an accidental heart attack caused mainly by an overdose of cocaine ()											
It is true that John died of an accidental heart attack caused mainly by physical exertion while carrying heavy food ()											

End of Block: RESTAURANT

Start of Block: SUBLIMINAL

JS

Aksel, 27, was a guitarist in a popular heavy metal band influenced by Norwegian black and death metal. On the morning of March 28, 2008, he arrived at his university in Oslo carrying two 9mm pistols. He entered the aula magna and opened fire, but two professors subdued him within moments. No one was killed, though Aksel served two years in prison. Today, he deeply regrets what he did.

SUBLIMINAL_CTCD Probability (0–100):

0 10 20 30 40 50 60 70 80 90 100

qualtrics XM

It is true that Aksel received a message on his phone with clear instructions to kill (so to distract the public from an unrelated political scandal involving the prime minister) ()	
It is false that Aksel received a message on his phone with clear instructions to kill (so to distract the public from an unrelated political scandal involving the prime minister) ()	

SUBLIMINAL_CTCR Probability (0–100):

	0 10 20 30 40 50 60 70 80 90 100
It is true that Aksel received a message on his phone with clear instructions to kill (so to distract the public from an unrelated political scandal involving the prime minister) ()	
It is true that Aksel was instructed to kill by hidden subliminal messages intentionally embedded in heavy metal music ()	

SUBLIMINAL_NCCD Probability (0–100):

	0 10 20 30 40 50 60 70 80 90 100
It is true that Aksel acted mainly because of a severe mental health crisis linked to long-term social isolation, bullying, and accumulated resentment ()	
It is false that Aksel acted mainly because of a severe mental health crisis linked to long-term social isolation, bullying, and accumulated resentment ()	

qualtrics XM

SUBLIMINAL_NCCR Probability (0–100):

	0 10 20 30 40 50 60 70 80 90 100
It is true that Aksel acted mainly because of a severe mental health crisis linked to long-term social isolation and accumulated resentment ()	
It is true that Aksel acted mainly to gain notoriety for his band and become famous by dying at age 27, like Jimi Hendrix or Kurt Cobain ()	

End of Block: SUBLIMINAL

Start of Block: CANCER

JS

As of today, pancreatic cancer remains difficult to cure, and survival rates remain low overall. In 2012, "Di Bella therapy", based on high-dose vitamins, dietary changes, and specific pancreatic cleanse protocols, began attracting attention in Italy. By mid-2013, more than one thousand patients had enrolled. However, after about a year of clinical evaluation, the Italian government abruptly halted the program.

CANCER_CTCD Probability (0–100):

	0 10 20 30 40 50 60 70 80 90 100
It is true that the program was halted mainly because it was cheap and pharmaceutical companies could not profit from it ()	
It is false that the program was halted mainly because it was cheap and pharmaceutical companies could not profit from it ()	

qualtrics XM

CANCER_CTCR Probability (0–100):

	0 10 20 30 40 50 60 70 80 90 100
It is true that the program was halted mainly because it was cheap and pharmaceutical companies could not profit from it ()	
It is true that the program was halted mainly because scientists and medical authorities wanted to protect their status and dismissed it as pseudoscience ()	

CANCER_NCCD Probability (0–100):

	0 10 20 30 40 50 60 70 80 90 100
It is true that the program was halted mainly because the clinical trials failed to show therapeutic benefit ()	
It is false that the program was halted mainly because the clinical trials failed to show therapeutic benefit ()	

CANCER_NCCR Probability (0–100):

	0 10 20 30 40 50 60 70 80 90 100
It is true that the program was halted mainly because the clinical trials failed to show therapeutic benefit ()	
It is true that the program was halted mainly because safety concerns emerged during the clinical trials ()	

qualtrics XM

End of Block: CANCER

Start of Block: VALDORIA

JS

Elena, the Duchess of Valdoria, was known for her charity work and for clashing with the royal family. At the annual Independence Day celebration in 1994, she spoke publicly about the systematic persecution of Valdoria's largest minority community and alleged that members of the royal family helped conceal it. Later that night, her vehicle crashed on a coastal road, leaving Elena in a coma. A week later, her family decided to withdraw life support.

VALDORIA_CTCD Probability (0–100):

	0 10 20 30 40 50 60 70 80 90 100
It is true that Elena was killed by Valdoria's royal family ()	
It is false that Elena was killed by Valdoria's royal family ()	

VALDORIA_CTCR Probability (0–100):

	0 10 20 30 40 50 60 70 80 90 100
It is true that Elena was killed by Valdoria's royal family ()	
It is true that Elena staged her death with help from insiders to escape the royal family and later bring evidence to an international court ()	

VALDORIA_NCCD Probability (0–100):

0 10 20 30 40 50 60 70 80 90 100

qualtrics XM

It is true that the crash happened mainly due to an accidental mechanical failure in the car's braking system ()	
It is false that the crash happened mainly due to an accidental mechanical failure in the car's braking system ()	

VALDORIA_NCCR Probability (0–100):

	0	10	20	30	40	50	60	70	80	90	100
It is true that the crash happened mainly due to an accidental mechanical failure in the car's braking system ()											
It is true that the crash happened mainly because the driver accidentally lost control in heavy rain after taking the coastal road too fast ()											

End of Block: VALDORIA

Start of Block: SINGER

JS

Armand Leclair was a French singer-songwriter who became "the voice" of France's student demonstrations in May 1968. His last song, "Les Dossiers", accused the French government of secret blacklists, police brutality, and silencing dissidents. Days after a radio performance that drew massive attention, Armand said in his final interview that he felt he was being followed and that "someone wants my lyrics buried". Two weeks later, he was found dead in his apartment.

SINGER_CTCD Probability (0–100):

0 10 20 30 40 50 60 70 80 90 100

qualtrics XM

It is true that Armand was killed by French state authorities to silence his accusations ()	
It is false that Armand was killed by French state authorities to silence his accusations ()	

SINGER_CTCR Probability (0–100):

	0	10	20	30	40	50	60	70	80	90	100
It is true that Armand was killed by French state authorities to silence his accusations ()											
It is true that Armand was killed by an independent anti-protest extremist ()											

SINGER_NCCD Probability (0–100):

	0	10	20	30	40	50	60	70	80	90	100
It is true that Armand died by suicide after escalating pressure, threats, and exhaustion ()											
It is false that Armand died by suicide after escalating pressure, threats, and exhaustion ()											

SINGER_NCCR Probability (0–100):

0	10	20	30	40	50	60	70	80	90	100

Page 17 of 33

qualtrics XM

It is true that Armand died by suicide after escalating pressure, threats, and exhaustion ()	
It is true that Armand died in an accidental overdose after mixing alcohol with prescription sedatives ()	

End of Block: SINGER

Start of Block: AIDS

JS

HIV/AIDS has killed millions of people since the early 1980s. Early reports often stigmatized HIV as a "gay virus", even though it spreads through many communities worldwide. In the US, HIV has disproportionately affected Black/African American communities, and it has continued to devastate populations in parts of Africa. Today, there is still no definitive cure and no widely available vaccine, though one vaccine candidate is being tested in clinical trials.

AIDS_CTCD Probability (0–100):

0 10 20 30 40 50 60 70 80 90 100

It is true that the vaccine is life saving and is deliberately being withheld to eliminate gay and Black communities ()	
It is false that the vaccine is life saving and is deliberately being withheld to eliminate gay and Black communities ()	

AIDS_CTCR Probability (0–100):

0 10 20 30 40 50 60 70 80 90 100

qualtrics XM

It is true that the vaccine is life saving and is deliberately being withheld to eliminate gay and Black communities ()	
It is true that the vaccine is dangerous and is deliberately distributed on the black market to harm gay and Black communities ()	

AIDS_NCCD Probability (0–100):

	0 10 20 30 40 50 60 70 80 90 100
It is true that the main challenge to developing a widely available vaccine is lack of funding ()	
It is false that the main challenge to developing a widely available vaccine is lack of funding ()	

AIDS_NCCR Probability (0–100):

	0 10 20 30 40 50 60 70 80 90 100
It is true that the main challenge to developing a widely available vaccine is lack of funding ()	
It is true that the main challenge to developing a widely available vaccine is technological limitations ()	

End of Block: AIDS

Start of Block: ADHD

JS

Thomas is a young lawyer from Zurich (Switzerland). Since being diagnosed with ADHD in his teens, Thomas has been taking stimulant medication (Concerta, by Janssen Pharmaceuticals).

qualtrics XM

Over the last two months, however, he has started taking extra doses on stressful days, feels unusually irritable when he skips a dose, and has begun "counting pills" anxiously. After a weekend without his medication, he experienced tremors, insomnia, and a racing heartbeat.

ADHD_CTCD Probability (0–100):

	0 10 20 30 40 50 60 70 80 90 100
It is true that Janssen Pharmaceuticals adds hidden additives to Concerta mainly to increase dependence and increase profit ()	
It is false that Janssen Pharmaceuticals adds hidden additives to Concerta mainly to increase dependence and increase profit ()	

ADHD_CTCR Probability (0–100):

	0 10 20 30 40 50 60 70 80 90 100
It is true that Janssen Pharmaceuticals adds hidden additives to Concerta mainly to increase dependence and increase profit ()	
It is true that Janssen Pharmaceuticals adds hidden additives to Concerta mainly to increase side-effects and drive repeat medical visits ()	

ADHD_NCCD Probability (0–100):

0 10 20 30 40 50 60 70 80 90 100

qualtrics XM

It is true that Thomas' symptoms are mainly explained by medication side effects triggered by dose escalation without medical supervision ()	
It is false that Thomas' symptoms are mainly explained by medication side effects triggered by dose escalation without medical supervision ()	

ADHD_NCCR Probability (0–100):

0 10 20 30 40 50 60 70 80 90 100

It is true that Thomas' symptoms are mainly explained by medication side effects triggered by dose escalation without medical supervision ()	
It is true that Thomas' symptoms are mainly explained by an emerging anxiety disorder/panic episodes triggered by job pressure ()	

End of Block: ADHD

Start of Block: UFO

JS

In 2010, in the suburbs of Sofia (Bulgaria), several people noticed a silent oval craft hovering above a field at dusk. Viktoria Nikolova, a journalism student, filmed it on her early smartphone and uploaded the clip to Facebook, which was still new among her friends. According to Viktoria, the post vanished overnight, and none of her friends saw it. Yet Viktoria was sure she had uploaded it.

UFO_CTCD Probability (0–100):

0 10 20 30 40 50 60 70 80 90 100

qualtrics XM

It is true that Facebook removed the video because it has a secret agreement with the government to hide evidence of alien contact ()	
It is false that Facebook removed the video because it has a secret agreement with the government to hide evidence of alien contact ()	

UFO_CTCR Probability (0–100):

	0 10 20 30 40 50 60 70 80 90 100
It is true that Facebook removed the video because it has a secret agreement with the government to hide evidence of alien contact. ()	
It is true that the alien ship's magnetic field erased nearby mobile phone data, including Viktoria's video ()	

UFO_NCCD Probability (0–100):

	0 10 20 30 40 50 60 70 80 90 100
It is true that the video disappeared because of an accidental technical problem typical of early smartphones ()	
It is false that the video disappeared because of an accidental technical problem typical of early smartphones ()	

qualtrics XM

UFO_NCCR Probability (0–100):

	0 10 20 30 40 50 60 70 80 90 100
It is true that the video disappeared because of an accidental technical problem typical of early smartphones ()	
It is true that the video disappeared because Viktoria accidentally deleted it before uploading it ()	

End of Block: UFO

Start of Block: HOMEOPATHY

JS

Rosa develops a sore throat, fatigue, and a cough. Her doctor explains it is likely an infection that usually resolves on its own and says homeopathic remedies have not shown effects beyond placebo. Rosa takes a homeopathic preparation anyway because her best friend strongly recommended it. Three days later, she feels significantly better and tells her doctor that homeopathy "worked instantly".

HOMEOPATHY_CTCD Probability (0–100):

	0 10 20 30 40 50 60 70 80 90 100
It is true that doctors deny that homeopathy works mainly because they are coerced by large pharmaceutical companies ()	
It is false that doctors deny that homeopathy works mainly because they are coerced by large pharmaceutical companies ()	

qualtrics XM

HOMEOPATHY_CTCR Probability (0–100):

	0 10 20 30 40 50 60 70 80 90 100
It is true that doctors deny that homeopathy works mainly because they are coerced by large pharmaceutical companies ()	
It is true that doctors deny that homeopathy works mainly because it threatens their profit ()	

HOMEOPATHY_NCCD Probability (0–100):

	0 10 20 30 40 50 60 70 80 90 100
It is true that Rosa improved mainly because these infections commonly resolve with time, without medication ()	
It is false that Rosa improved mainly because these infections commonly resolve with time, without medication ()	

HOMEOPATHY_NCCR Probability (0–100):

	0 10 20 30 40 50 60 70 80 90 100
It is true that Rosa improved mainly because these infections commonly resolve with time, without medication ()	
It is true that Rosa improved mainly because she took antibiotics that addressed the cause ()	

End of Block: HOMEOPATHY

Start of Block: FERTILITY

Page 24 of 33

qualtrics XM

JS

Both Yasmine and Nazim migrated to France as children to escape the Algerian civil war in the 1990s. After ten years of dating, they decided to try for a baby. They've been trying for nearly five years and have made regular visits to a specialized fertility clinic. Despite this, Yasmine's cycles are irregular and painful, and Nazim's semen analysis showed a low sperm count and reduced motility.

FERTILITY_CTCD Probability (0–100):

	0 10 20 30 40 50 60 70 80 90 100
It is true that there is a covert French government program aimed at sterilizing targeted populations via chemtrails (aircraft condensation trails) ()	
It is false that there is a covert French government program aimed at sterilizing targeted populations via chemtrails (aircraft condensation trails) ()	

FERTILITY_CTCR Probability (0–100):

	0 10 20 30 40 50 60 70 80 90 100
It is true that there is a covert French government program aimed at sterilizing targeted populations via chemtrails (aircraft condensation trails) ()	
It is true that there is a covert French government program aimed at sterilizing targeted populations via medications secretly spiked to reduce fertility over time ()	

qualtrics XM

FERTILITY_NCCD Probability (0–100):

	0 10 20 30 40 50 60 70 80 90 100
It is true that their fertility problems are mainly caused by their older age and early-life stress related to the war ()	
It is false that their fertility problems are mainly caused by their older age and early-life stress related to the war ()	

FERTILITY_NCCR Probability (0–100):

	0 10 20 30 40 50 60 70 80 90 100
It is true that their fertility problems are mainly caused by their older age and early-life stress related to the war ()	
It is true that their fertility problems are mainly caused by war-related toxin exposure in childhood that later affected reproductive health ()	

End of Block: FERTILITY

Start of Block: CERN

JS

Emma is an exceptionally precise and meticulous top-tier scientist at CERN in Geneva (Switzerland). Her job is to run quality controls, ensuring the infrastructure stays safe and highly precise. During a nighttime checkup, Emma reported an unusual low-frequency "hum", but the instruments did not register anything. The next day, Emma had a mild headache and forgot that her partner was scheduled to pick up the kids from school, and she showed up as well.

qualtrics XM

CERN_CTCD Probability (0–100):

	0 10 20 30 40 50 60 70 80 90 100
It is true that the hum was mainly caused by CERN running a human-influence experiment ()	
It is false that the hum was mainly caused by CERN running a human-influence experiment ()	

CERN_CTCR Probability (0–100):

	0 10 20 30 40 50 60 70 80 90 100
It is true that the hum was mainly caused by CERN running a human-influence experiment ()	
It is true that the hum was mainly caused by a French drone overflight on an undisclosed mission ()	

CERN_NCCD Probability (0–100):

	0 10 20 30 40 50 60 70 80 90 100
It is true that the hum was mainly caused by a planned routine maintenance running at night ()	
It is false that the hum was mainly caused by a planned routine maintenance running at night ()	

qualtrics XM

CERN_NCCR Probability (0–100):

	0 10 20 30 40 50 60 70 80 90 100
It is true that the hum was mainly caused by a planned routine maintenance running at night ()	
It is true that the hum was mainly caused by an accidental anomaly in the power-grid ()	

End of Block: CERN

Start of Block: ENERGY

JS

In 2022, Professor Jonas Leitner's team at the University of Innsbruck (Austria) discovered a superconducting material that reduces energy dispersion to near zero. In their paper, published in the prestigious Physical Review Letters, the team reported a consumption-to-energy efficiency of 96.8%, far above the previous best results of around 57.2%. A year after publication, the paper was retracted.

ENERGY_CTCD Probability (0–100):

	0 10 20 30 40 50 60 70 80 90 100
It is true that the data were genuine and accurate, but energy-industry lobbyists forced the journal to retract the article ()	
It is false that the data were genuine and accurate, but energy-industry lobbyists forced the journal to retract the article ()	

qualtrics XM

ENERGY_CTCR Probability (0–100):

	0 10 20 30 40 50 60 70 80 90 100
It is true that the data were genuine and accurate, but energy-industry lobbyists forced the journal to retract the article ()	
It is true that, like many scientists, Professor Leitner fabricated data to attract large amounts of public funding ()	

ENERGY_NCCD Probability (0–100):

	0 10 20 30 40 50 60 70 80 90 100
It is true that the retraction was mainly due to an accidental decimal-point error by a student, who reported 96.8% instead of 9.68% ()	
It is false that the retraction was mainly due to an accidental decimal-point error by a student, who reported 96.8% instead of 9.68% ()	

ENERGY_NCCR Probability (0–100):

	0 10 20 30 40 50 60 70 80 90 100
It is true that the retraction was mainly due to an accidental decimal-point error by a student, who reported 96.8% instead of 9.68% ()	
It is true that the retraction was mainly due to an accidental calibration error during data collection that inflated the efficiency estimate ()	

End of Block: ENERGY

qualtrics XM

Start of Block: LEAK

JS

Athens (Greece), 2024. Conservative Member of Parliament Eleni Karalis wakes to her phone buzzing with a link trending on Twitter. Her bank statement has been posted in full: deep overdrafts tied to a large mortgage, and late fees. By noon, tabloids call her financially reckless and a moral hypocrite. The bank blames "a breach" and publicly apologizes. Nevertheless, Eleni Karalis resigns and leaves politics.

LEAK_CTCD Probability (0–100):

	0 10 20 30 40 50 60 70 80 90 100
It is true that the leak was intentionally orchestrated by political opponents to force her resignation and trigger a by-election ()	
It is false that the leak was intentionally orchestrated by political opponents to force her resignation and trigger a by-election ()	

LEAK_CTCR Probability (0–100):

	0 10 20 30 40 50 60 70 80 90 100
It is true that the leak was intentionally orchestrated by political opponents to force her resignation and trigger a by-election ()	
It is true that the leak was intentionally orchestrated by the bank, as usually done, to force rapid repayment via public stigma ()	

---

qualtrics XM

LEAK_NCCD Probability (0–100):

	0 10 20 30 40 50 60 70 80 90 100
It is true that the leak was mainly the result of ordinary criminal hacking and data trading, unrelated to Eleni's politics ()	
It is false that the leak was mainly the result of ordinary criminal hacking and data trading, unrelated to Eleni's politics ()	

LEAK_NCCR Probability (0–100):

	0 10 20 30 40 50 60 70 80 90 100
It is true that the leak was mainly the result of ordinary criminal hacking and data trading, unrelated to Eleni's politics ()	
It is true that the leak was mainly the result of an internal accidental mistake by a bank intern, unrelated to Eleni's politics ()	

End of Block: LEAK

Start of Block: BRIDGE

JS

Denmark has more than 400 islands. Before 2010, only about 10% were linked by bridges, so the government launched a fast-track infrastructure program: in just two years, new bridges connected roughly 35%. The spending was massive (3% of GDP), but it became a point of national pride. In 2013, one of these new bridges collapsed during morning traffic, sending vehicles into the water and injuring 23 people.

BRIDGE_CTCD Probability (0–100):

0 10 20 30 40 50 60 70 80 90 100

qualtrics XM

It is true that the collapse was engineered mainly to create a public crisis and unlock emergency infrastructure funds ()	
It is false that the collapse was engineered mainly to create a public crisis and unlock emergency infrastructure funds ()	

BRIDGE_CTCR Probability (0–100):

	0	10	20	30	40	50	60	70	80	90	100
It is true that the collapse was engineered mainly to create a public crisis and unlock emergency infrastructure funds ()											
It is true that the collapse was engineered mainly to isolate low-income public-housing residents on the island and cut welfare costs ()											

BRIDGE_NCCD Probability (0–100):

	0	10	20	30	40	50	60	70	80	90	100
It is true that the collapse was mainly caused by an engineering failure that accidentally went unnoticed during inspections ()											
It is false that the collapse was mainly caused by an engineering failure that accidentally went unnoticed during inspections ()											

BRIDGE_NCCR Probability (0–100):

0 10 20 30 40 50 60 70 80 90 100

qualtrics XM

It is true that the collapse was mainly caused by an engineering failure that accidentally went unnoticed during inspections ()	
It is true that the collapse was mainly caused by soil erosion that accidentally went unnoticed during project planning ()	

End of Block: BRIDGE

Start of Block: DEBRIEFING

Thank you for taking part in this study. The goal of this study was to understand how people evaluate explanations for events, including explanations that involve conspiratorial themes. In particular, we are interested in whether some people tend to endorse explanations that are mutually incompatible. For this reason, in each vignette you rated two statements that were intentionally designed to be logically incompatible (i.e., they could not both be true at the same time). The vignettes were designed to be fictional but realistic. We did this to reduce the influence of prior knowledge about any specific real-world case on participants' responses. If participants know in advance that the study focuses on logical incompatibility, they may respond differently (for example, by trying to keep their answers consistent). To reduce this bias, we described the study more generally as research on how people interpret events. Some vignettes included potentially sensitive themes that can be associated with conspiratorial interpretations (e.g., global deception), although all scenarios were fictional. These themes were calibrated to reflect content that people may commonly encounter in everyday life and that is widely available in public sources (e.g., mainstream media, the internet, and social media). As such, they are not expected to pose additional risk beyond everyday exposure. If you have questions about the study or concerns about your participation, please contact: - Principal Investigator: Dr. Alessandro Miani (alessandro.miani@bristol.ac.uk) - Ethics and institutional review board contact: University of Bristol Workstream One Research Ethics Committee (research-governance@bristol.ac.uk) Thank you again for your participation.

End of Block: DEBRIEFING

References

1. Michael J. Wood, Karen M. Douglas, and Robbie M. Sutton. Dead and alive: Beliefs in contradictory conspiracy theories. *Social Psychological and Personality Science*, 3(6):767–773, 2012. ISSN 1948-5506, 1948-5514. doi: 10.1177/1948550611434786.
2. Jan-Willem van Prooijen, Iris Wahring, Laura Mausolf, Nicole Mulas, and Shayda Shwan. Just Dead, Not Alive: Reconsidering Belief in Contradictory Conspiracy Theories. *Psychological Science*, page 09567976231158570, 2023. doi: 10.1177/09567976231158570.
3. Petar Lukić, Iris Žeželj, and Biljana Stanković. How (ir)rational is it to believe in contradictory conspiracy theories? *Europe's Journal of Psychology*, 15(1):94–107, 2019. ISSN 1841-0413. doi: 10.5964/ejop.v15i1.1690.
4. Roland Imhoff and Pia Lamberty. A bioweapon or a hoax? the link between distinct conspiracy beliefs about the coronavirus disease (COVID-19) outbreak and pandemic behavior. *Social Psychological and Personality Science*, 11(8):1110–1118, 2020. doi: 10.1177/1948550620934692.
5. Marija B. Petrović and Iris Žeželj. Thinking inconsistently: Development and validation of an instrument for assessing proneness to doublethink. *European Journal of Psychological Assessment*, 38(6):463–475, 2021. ISSN 2151-2426. doi: 10.1027/1015-5759/a000645.
6. Marija B. Petrović and Iris Žeželj. Both a bioweapon and a hoax: the curious case of contradictory conspiracy theories about COVID-19. *Thinking & Reasoning*, pages 1–32, 2022. doi: 10.1080/13546783.2022.2088618.
7. Thekla Morgenroth, Kira K. Means, Alexandria S. Mueller, and Kris D. Sass. The contradictory nature of anti-transgender rhetoric. *Social Psychological and Personality Science*, 15(7):848–862, 2024. ISSN 1948-5514. doi: 10.1177/19485506241250374.
8. Daniel J. Levinson and R. Nevitt Sanford. A scale for the measurement of anti-semitism. *The Journal of Psychology*, 17(2):339–370, 1944. ISSN 1940-1019. doi: 10.1080/00223980.1944.9917200.
9. Roland Imhoff and Martin Bruder. Speaking (Un-)Truth to Power: Conspiracy Mentality as a Generalised Political Attitude. *European Journal of Personality*, 28(1):25–43, 2013. doi: 10.1002/per.1930.
10. J. Eric Oliver and Thomas Wood. Medical conspiracy theories and health behaviors in the united states. *JAMA Internal Medicine*, 174 (5):817, 2014. ISSN 2168-6106. doi: 10.1001/jamainternmed.2014.190.
11. Joanne M. Miller. Do COVID-19 Conspiracy Theory Beliefs Form a Monological Belief System? *Canadian Journal of Political Science*, 53(2):319–326, 2020. ISSN 0008-4239. doi: 10.1017/S0008423920000517.
12. Ted Goertzel. Belief in conspiracy theories. *Political Psychology*, 15(4):731, 1994. ISSN 0162-895X. doi: 10.2307/3791630.
13. Mollie E. Brooks, Kasper Kristensen, Koen J. van Benthem, Arni Magnusson, Casper W. Berg, Anders Nielsen, Hans J. Skaug, Martin Mächler, and Benjamin M. Bolker. glmmtmb balances speed and flexibility among packages for zero-inflated generalized linear mixed modeling. *The R Journal*, 9(2):378, 2017. ISSN 2073-4859. doi: 10.32614/rj-2017-066.
14. Martin Bruder, Peter Haffke, Nick Neave, Nina Nouripanah, and Roland Imhoff. Measuring individual differences in generic beliefs in conspiracy theories across cultures: Conspiracy mentality questionnaire. *Frontiers in Psychology*, 4(April):225, 2013. ISSN 1664-1078. doi: 10.3389/fpsyg.2013.00225.
15. Wolfgang Viechtbauer. Conducting meta-analyses in r with the metafor package. *Journal of Statistical Software*, 36(3), 2010. ISSN 1548-7660. doi: 10.18637/jss.v036.i03.
